# Carbon emissions reductions from Indonesia’s moratorium on forest concessions are cost-effective yet contribute little to Paris pledges

**DOI:** 10.1073/pnas.2102613119

**Published:** 2022-01-24

**Authors:** Ben Groom, Charles Palmer, Lorenzo Sileci

**Affiliations:** ^a^Department of Economics, University of Exeter, Exeter EX4 4PY, United Kingdom;; ^b^Grantham Research Institute on Climate Change and the Environment, London School of Economics and Political Science, London WC2A 2AE, United Kingdom;; ^c^Department of Geography and Environment, London School of Economics and Political Science, London WC2A 2AE, United Kingdom

**Keywords:** carbon price, deforestation, Indonesia, NDC, REDD+

## Abstract

International initiatives for reducing carbon emissions from deforestation and forest degradation (REDD+) could make critical, cost-effective contributions to tropical countries’ nationally determined contributions (NDCs). Norway, a key donor of such initiatives, had a REDD+ partnership with Indonesia, offering results-based payments in exchange for emissions reductions calculated against a historical baseline. Central to this partnership was an area-based moratorium on new oil palm, timber, and logging concessions in primary and peatland forests. We evaluate the effectiveness of the moratorium between 2011 and 2018 by applying a matched triple difference strategy to a unique panel dataset. Treated dryland forest inside moratorium areas retained, at most, an average of 0.65% higher forest cover compared to untreated dryland forest outside the moratorium. By contrast, carbon-rich peatland forest was unaffected by the moratorium. Cumulative avoided dryland deforestation from 2011 until 2018 translates into 67.8 million to 86.9 million tons of emissions reductions, implying an effective carbon price below Norway’s US$5 per ton price. Based on Norway’s price, our estimated cumulative emissions reductions are equivalent to a payment of US$339 million to US$434.5 million. Annually, our estimates suggest a 3 to 4% contribution to Indonesia’s NDC commitment of a 29% emissions reduction by 2030. Despite the Indonesia–Norway partnership ending in 2021, reducing emissions from deforestation remains critical for meeting this commitment. Future area-based REDD+ initiatives could build on the moratorium’s outcomes by reforming its incentives and institutional arrangements, particularly in peatland forest areas.

## Significance

More than a decade after the global adoption of REDD+ as a climate change mitigation strategy, countries have started accessing results-based payments. However, the extent to which payments are actually based on results is unknown, necessitating program evaluations to establish the contribution of REDD+ to the Paris NDCs. We undertake a microeconometric evaluation of one of the most globally significant REDD+ initiatives, Indonesia’s moratorium on forest concessions, in which a payment has been awarded. At the agreed US$5/tCO_2_-eq, the value of our estimated cumulative carbon emissions far exceeds the proposed payment from the donor, Norway. Although cost-effective, the emissions reductions only contribute 3 to 4% of Indonesia’s NDC. This contribution could be increased in new initiatives with better-designed incentives and institutional arrangements.

Deforestation and forest degradation account for ∼10% of global greenhouse gas emissions (1). Recognizing the importance of slowing deforestation in efforts to mitigate global warming, an international framework for Reducing Emissions from Deforestation and Forest Degradation (REDD+) was established in 2007 at the 13th Conference of the Parties (COP) (2). At this COP, Norway’s government announced its International Climate and Forest Initiative, pledging up to $300 million every year toward REDD+. Norway’s funds have been channeled through a number of negotiated, bilateral deals with countries hosting tropical forests. Countries include Brazil, Guyana, Tanzania, and the setting for our study, Indonesia, where, between 2000 and 2010, lowland evergreen forests and peat swamp forests were deforested by 1.2% and 2.2% per year, respectively (3).

Indonesia is one of the world’s largest greenhouse gas (GHG) emitters. Between 2000 and 2016, ∼50% of Indonesia’s annual emissions were generated from deforestation, forest degradation, peatland decomposition, and peat fires, accounting for around a quarter of global emissions from these sources (4, 5). The country’s partnership with Norway, established in 2010, included a pledge of US$1 billion to fund “results-based” REDD+ payments (6). Central to this partnership was a moratorium on the granting of new concession licenses by district governments for the conversion of primary dryland and peatland forest into new palm oil, timber, and logging concessions (7, 8). Such concessions, operating across the archipelago (*SI Appendix*, Fig. S1), have been estimated to be responsible for almost half of Indonesia’s forest loss (9, 10). Implemented in 2011, the moratorium initially covered 69 million hectares of forest across the country (11), most of Indonesia’s forest estate (*SI Appendix*, Fig. S2). Additional restrictions on the conversion of peatland forest, affecting all concession types, were implemented across Indonesia in 2017 (12), and, in late 2018, a new 3-y moratorium on new palm oil concessions was also imposed nationally (13).

In 2017, Indonesia reportedly reduced emissions from deforestation and forest degradation by 11.2 MtCO_2_-eq (14). Norway subsequently announced that it would pay Indonesia US$56.2 million (15) based on a carbon price of US$5/tCO_2_-eq. In this article, we ask whether Norway is getting carbon value for its money, that is, whether this payment is actually based on results. The extent to which the moratorium has had any meaningful impact on deforestation has been the subject of intense debate in Indonesia, particularly after it became permanent in 2019 (14). Although the REDD+ partnership was terminated by Indonesia’s government in 2021 (16), an effective moratorium since 2011 could contribute to meeting Indonesia’s nationally determined contribution (NDC) commitment of reducing GHG emissions by 29% unconditionally (and up to 41% conditionally) by 2030 (4). Indeed, large-scale REDD+ initiatives play a potentially critical role in global climate change mitigation efforts (17, 18), and, more than a decade after the 13th COP, Indonesia is among a number of countries that have begun moving toward REDD+ implementation and accessing results-based payments (19).

Norway’s US$1 billion pledge to Indonesia emphasizes the global role of Norway in the design and funding of international REDD+ strategies (20). This pledge acted as an incentive to Indonesia’s national government to enforce the moratorium. A measurement, reporting, and verification system was developed, and, although district governments had an enforcement role (13), there was little evidence of coordination, or of a plan to share benefits, between the national and district governments (11, 21). Effective coordination might have prevented, or at least influenced, changes in the moratorium’s boundaries due to the redesignation of forestland by district governments (13, 22). That redesignated forestland was often subsequently licensed out to concessionaires has raised concerns about corruption among government officials (13). Long endemic in Indonesia’s forest sector (22–24), corruption exacerbates the country’s weak capacity to monitor and enforce forest regulations, characterized by, for example, limited budgets and personnel (25, 26). In sum, we anticipate little or no impact of the moratorium on deforestation. A best-case scenario from previous research, an ex ante simulation of the moratorium as the counterfactual to actual land uses between 2000 and 2010 (prior to the implementation of the moratorium) and assuming 100% compliance (or effectiveness) (27), indicates a maximum 3.5% reduction in deforestation and a 7.2% reduction in emissions.

While this best-case scenario was based on econometric analysis, the payment for the emissions reduction in 2017 was estimated by comparing, for the whole country, the amount of deforestation observed in 2017 against a historical baseline based on the average annual level of deforestation observed between 2006 and 2016 (28). The use of a historical baseline as a counterfactual provides weak evidence that the moratorium has had a causal effect on REDD+ outcomes, because deforestation in any given year will vary due to stochastic natural processes (e.g., weather and fires) and economic factors (e.g., demand for commodities). Thus, it is highly unlikely that observed deviations from the average deforestation rate can be meaningfully related to the performance of the moratorium, and, instead, such deviations could be overestimated or underestimated by chance.

Our analysis begins with the observation that any measurable policy effect could only have been generated by forest areas covered by the moratorium. Estimating a policy effect requires a comparison of forest areas covered by the moratorium with a counterfactual that mimics what would have happened in those areas had the moratorium not been implemented. The intention of the counterfactual is to ensure that all other (nonmoratorium) factors relevant for determining the economic viability of palm oil and timber production in forest areas (29–31) are the same. Similar to networks of protected areas, moratorium areas were not randomly assigned (32, 33), and the small size of the reductions simulated by ref. 27 imply relatively low returns from forest conversion in these areas. Estimating the impact of the moratorium on deforestation is therefore hampered by preexisting differences in levels and the likely trajectory of deforestation between moratorium and nonmoratorium areas. Furthermore, district governments could continue to issue licenses for new concessions in forestland outside moratorium areas. One response to the moratorium might be for licenses planned for moratorium areas to be granted in nonmoratorium areas instead. Such spatial spillovers (“leakage”), a common concern in forest conservation (e.g., refs. 32–36), also confound estimates of moratorium impact, potentially making it look more successful compared to nonmoratorium areas, when, in fact, activities have just been displaced. By testing for leakage, much can be learned about the processes that govern successful—or poor—performances. None of these confounding effects is specifically accounted for in the estimates of the payment offered by Norway’s government (*SI Appendix*, section 1).

To address confounding factors and isolate the moratorium’s impacts on deforestation and associated emissions, we undertake a program evaluation using quasi-experimental methods: a matched triple difference strategy (37) applied to Global Forest Change data (38) at the 1.2-km by 1.2-km scale between 2004 and 2018 (*Materials and Methods*). A matched difference-in-differences estimator controls for the different levels of forest cover in moratorium and nonmoratorium areas, and removes the deforestation trend in nonmoratorium areas matched by 1.2-km^2^ grid cells. Matching on, for example, proximity to markets and topography means that moratorium and nonmoratorium grid cells have similar probabilities of concession-driven forest loss. The triple difference step removes any remaining deviations in deforestation trends prior to the moratorium commencing in 2011, which otherwise would be attributed to the moratorium (39, 40). The impact that remains once these confounding factors have been addressed can then be attributed to the moratorium. In principle, only estimates that have taken seriously the nonrandom assignment of the moratorium should inform the Norwegian government’s results-based payments for emissions reductions. We test the robustness of our estimates in several ways (*Materials and Methods*). In particular, the potential for leakage as a response to the moratorium is tested with a regression discontinuity analysis applied to the boundaries of moratorium areas each year between 2005 and 2018. Thus, we estimate the differences in deforestation rates between each side of the moratorium’s boundaries. By comparing these differences from before to after the start of the moratorium in 2011, our analysis provides suggestive evidence for or against the presence of spillovers.

After evaluating the robustness of model estimates, we convert the moratorium’s impacts on forest cover change into carbon dioxide equivalents for comparison with estimates generated by the Indonesia–Norway partnership. On the basis of our program evaluation approach, we conclude that Norway’s government would be getting carbon value for money, but the emissions reductions generated by the moratorium contribute relatively little to Indonesia’s NDC commitments.

## Results

### Forest Cover Trends in Indonesia.

**Fig. 1.**
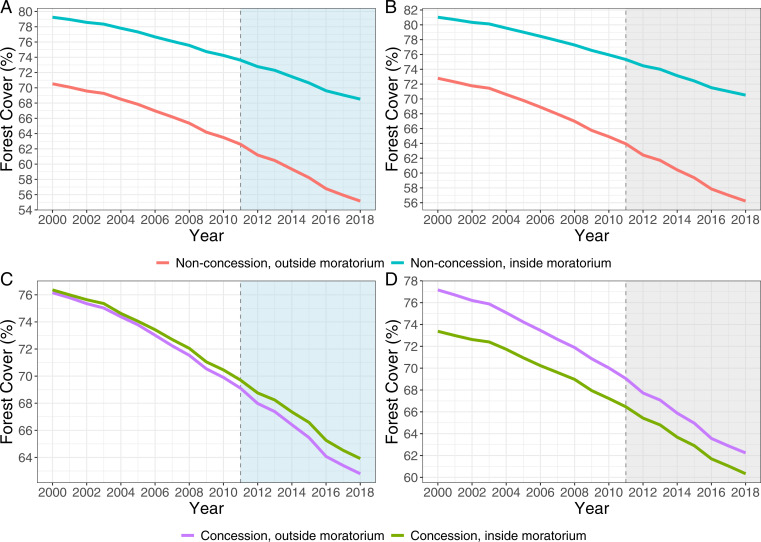
Forest cover trends inside and outside the moratorium, 2000–2018: nonconcession dryland grid cells (*A*), nonconcession peatland grid cells (*B*), concession dryland grid cells (*C*), and concession peatland grid cells (*D*). Shaded areas denote treatment period. Grid cells in *A* and *B* also exclude forest in protected areas.

Our outcome variable is “forest cover,” either dryland or peatland, in hectares. Concessions established within the moratorium’s 2011 boundaries prior to the start of the moratorium were legally allowed to continue operating, business as usual (BAU), after 2011. Thus, in Fig. 1, we distinguish between forest cover trends observed in forest areas located outside (Fig. 1 *A* and *B*) and inside (Fig. 1 *C* and *D*) concessions. Overall, the proportion of forest cover has declined, both inside and outside the moratorium’s boundaries, by ∼10 to 15 percentage points between 2000 and 2018. By comparing trends inside the moratorium to those outside, we observe that the rate of decline differs.

The extent of forest cover outside concessions is, on average, higher inside the moratorium compared to outside, and a steeper decline in forest cover is observed outside the moratorium compared to inside (Fig. 1 *A* and *B*). These trends suggest that the moratorium’s impacts from 2011 onward can only realistically stem from differences in the negative trends in forest cover between moratorium and nonmoratorium areas. A similar pattern of decline is also observed in the trends for concessions. The extent of dryland forest cover is almost the same when we compare concessions inside the moratorium to those outside (Fig. 1*C*). By contrast, the extent of peatland forest cover is higher in concessions outside the moratorium compared to concessions inside (Fig. 1*D*). Unaffected by the moratorium in principle, we use concessions as a placebo (falsification) test in our empirical analysis (*Materials and Methods*).

### National-Level Effects of the Moratorium.

**Fig. 2.**
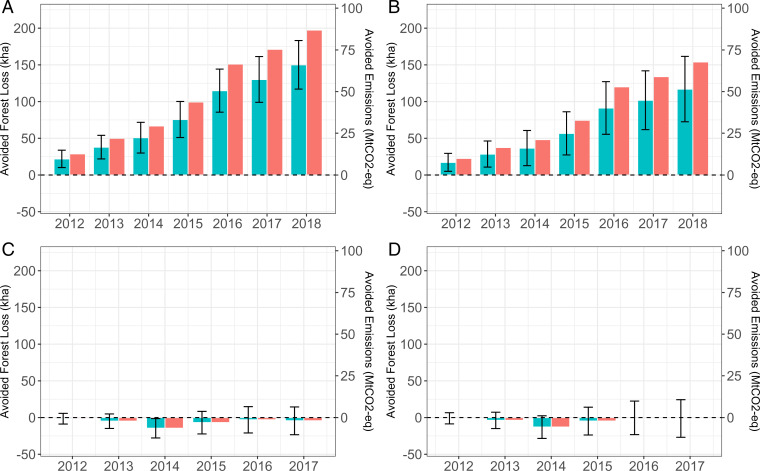
Cumulative avoided forest loss (thousand hectares) and avoided carbon dioxide emissions (MtCO_2_-eq): dryland forest DD, 2012–2018 (*A*); dryland forest DDD, 2012–2018 (*B*); peatland forest DD, 2012–2017 (*C*); and peatland DDD, 2012–2017 (*D*). The blue columns and left-hand *y* axis in each panel show the quantity of avoided forest loss, while the red columns and right-hand *y* axis show the quantity of carbon emissions avoided. All quantities are aggregated up to the level of the whole moratorium. Error bars denote the 95% CI.

Cumulative avoided forest loss and carbon emissions are estimated separately for dryland forest (Fig. 2 *A* and *B*) and peatland forest (Fig. 2 *C* and *D*), based on estimates of the average treatment effect on the treated (ATT). In the spirit of ref. 32, two estimators are presented to show the range of plausible results: the nonparametric difference-in-difference (DD: upper-bound estimate) and triple-difference (DDD: lower-bound estimate) approaches. We prefer the DDD estimator because it has a correction for nonparallel trends implied by the partial failure of the DD estimator of our placebo tests (placebo treatments prior to 2011), suggesting nonparallel trends even after matching. The differences in estimates shown in Fig. 2 demonstrate the importance of examining the parallel trends assumption. Further placebo tests using concessions provide further support for the DDD approach (*Materials and Methods*).

Our ATT estimates are equivalent to the amount of forest loss, in hectares, avoided in each grid cell of 144 ha. The ATT for dryland forest (*SI Appendix*, Tables S1 and S2) ranges between (with P≤0.000 where it is not reported) 0.108 (*P* = 0.006) and 0.137 in 2011–2012; 0.178 (*P* = 0.002) and 0.237 in 2011–2013; 0.229 (*P* = 0.003) and 0.318 in 2011–2014; 0.354 and 0.472 in 2011–2015; 0.571 and 0.718 in 2011–2016; 0.637 and 0.813 in 2011–2017; and 0.732 and 0.938 in 2011–2018. Despite the magnitude of impact increasing steadily since 2011, the moratorium was effective in protecting no more than 1 ha of dryland forest in each grid cell by 2018, or about 0.651% of a cell. Our ATT estimates for peatland forest (*SI Appendix*, Tables S3 and S4) are close to zero and not statistically significant at conventional levels.

The moratorium has had a relatively small cumulative impact in preventing deforestation in dryland forest covered by the moratorium relative to comparable forest areas outside the moratorium (Fig. 2 *A* and *B* and *SI Appendix*, Tables S5 and S6): 17,248 ha to 21,967 ha in 2011–2012; 28,533 ha to 37,972 ha in 2011–2013; 36,672 ha to 50,830 ha in 2011–2014; 56,725 ha to 75,603 ha in 2011–2015; 91,303 ha to 114,901 ha in 2011–2016; 101,851 ha to 130,168 ha in 2011–2017; and 117,053 ha to 150,089 ha in 2011–2018. Put in context, avoided dryland forest loss represents, at most, 0.03% of all land covered by the moratorium in 2012, rising over sevenfold to 0.22% by 2018. Our estimates of avoided dryland forest loss translate into cumulative carbon emission reductions of 10.0 MtCO_2_-eq to 12.7 MtCO_2_-eq in 2011–2012; 16.5 MtCO_2_-eq to 22.0 MtCO_2_-eq in 2011–2013; 21.2 MtCO_2_-eq to 29.4 MtCO_2_-eq in 2011–2014; 32.9 MtCO_2_-eq to 43.8 MtCO_2_-eq in 2011–2015; 52.9 MtCO_2_-eq to 66.6 MtCO_2_-eq in 2011–2016; 59.0 MtCO_2_-eq to 75.4 MtCO_2_-eq in 2011–2017; and 67.8 MtCO_2_-eq to 86.9 MtCO_2_-eq in 2011–2018 (*SI Appendix*, Tables S5 and S6). By contrast, the moratorium had null effects on peatland forest (Fig. 2 *C* and *D* and *SI Appendix*, Tables S7 and S8), implying a high likelihood of few if any carbon emissions savings, including those from peat drainage and peat fires.

Our results in Fig. 2 are checked for their robustness (*Materials and Methods*). First, we trim the sample on the basis of forest cover extent at the grid cell scale (DD and DDD; *SI Appendix*, Tables S9–S12). Second, we apply coarsened exact matching (CEM) models (DDD only; *SI Appendix*, Tables S13 and S14). Third, we apply a wider caliper (0.001) (DDD only; *SI Appendix*, Tables S15 and S16) and 1:2, 1:3, and 1:5 nearest-neighbor matching (DDD only; *SI Appendix*, Tables S17 and S18). Fourth, we estimate the ATT using only observations above an elevation of 1,000 m, followed by all elevations (DDD only; *SI Appendix*, Tables S17 and S18). These four checks are shown for 2011–2017 only, although the results for other years are also consistent with those in Fig. 2. Our estimates of forest loss and carbon emissions avoided above an elevation of 1,000 m are either very low (peatland) or not statistically significant (dryland) (*SI Appendix*, Fig. S3). Finally, using forest cover data based on a tighter definition of forest—“intact primary” forests with no detectable signs of human-caused alteration or fragmentation (41, 42)—we find patterns of avoided forest loss and carbon emissions that are consistent with those in Fig. 2 (*SI Appendix*, Tables S19–S26). Unsurprisingly, estimates of the extent of cumulative avoided dryland forest loss and carbon emissions are lower, around 25 to 35% of our 2018 estimates in Fig. 2 (see also *SI Appendix*, Fig. S4).

### Testing for Leakage.

**Fig. 3.**
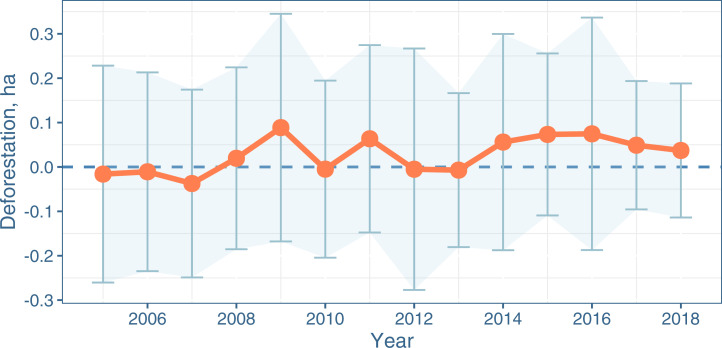
Regression discontinuity LATE [with Calonico et al. (43) bandwidth], 2005–2018. Scatterplots of all the observations within and outside the moratorium’s boundaries are shown in *SI Appendix*, section 4. Error bars denote the 95% CI.

Our estimated effects in Fig. 2 are relatively small, implying a low probability of upward bias due to leakage. We report the results of a regression discontinuity analysis along the moratorium’s boundaries, including the treatment effects at the discontinuity each year, between 2005 and 2018 (Fig. 3). The point estimate of this local average treatment effect (LATE) is always positive after 2013, implying higher deforestation outside the moratorium. Yet, the point estimates are also positive in 2008, 2009, and 2011, and hover around zero for the entire sample period, excluding an upward deviation in trend after the moratorium was implemented. The 95% CIs for the LATE always include zero, thus failing to identify significant leakage from the moratorium to the surrounding areas, either before or after 2011. Our analysis over various bandwidths suggests no evidence of leakage even within some considerable distance from the moratorium (*SI Appendix*, section 4).

### Meeting Indonesia’s NDC Commitments and the Effective Carbon Price.

We put our results into perspective by comparing our estimates of emissions reductions between 2011 and 2017 with Indonesia’s aggregate emissions and its NDC emissions reduction commitments (Table 1). Our estimated average annual emissions reductions, of around 10.4 MtCO_2_-eq to 13.0 MtCO_2_-eq (including our peatland forest estimates and factoring in belowground carbon), at most, comprise only 0.38 to 0.47% of annual aggregate emissions (all sectors), and 0.83 to 1.05% of emissions from the forest sector. The emissions avoided due to the moratorium comprise 10.3 to 12.9% and 3.1 to 3.8% of the 29% (unconditional) NDC target in 2020 and 2030, respectively. These shares fall to 7.8 to 9.8% (2020) and 2.0 to 2.5% (2030) when we consider the 41% (conditional) NDC target.

**Table 1.** The moratorium’s contribution to Indonesia’s NDC commitments and the effective carbon price, 2011–2017

Estimator	DD	DDD
Avoided emissions (aboveground carbon only, MtCO_2_-eq)		
Dryland	75.4	59.0
Peatland	–2.0	–0.6
Total	73.4	58.4
Annual average (2011–2017)	10.5	8.3
Avoided emissions (aboveground and belowground carbon,		
MtCO_2_-eq)		
Total	91.1	72.5
Annual average (2011–2017)	13.0	10.4
Percent of Indonesia’s emissions (aboveground carbon only)		
Percent all emissions (2.2GtCO2/y)	0.47	0.38
Percent forest emissions (1.0GtCO2/y)	1.05	0.83
Percent of Indonesia’s emissions (aboveground and belowground		
carbon)		
Percent all emissions (2.2GtCO2/y)	0.59	0.47
Percent forest emissions (1.0GtCO2/y)	1.30	1.04
Comparison with Indonesia’s NDC 2030 commitments (%)		
Percent unconditional (29%, 2020)	12.9	10.3
Percent conditional (41%, 2020)	9.8	7.8
Percent unconditional (29%, 2030)	3.8	3.1
Percent conditional (41%, 2030)	2.5	2.0
Effective carbon price (US$/tCO_2_)		
With peatland payments (US$56m/total)	$0.6	$0.8
With peatland payments (US$56m/annual average)	$4.3	$5.4
No peatland payments (US$24m/total)	$0.3	$0.3
No peatland payments (US$24m/annual average)	$1.8	$2.3

DD denotes that the results are derived from the ATT estimated using the nonparametric difference-in-difference approach. DDD denotes that the results are derived from the ATT estimated using the nonparametric triple difference approach. Underlying ATT estimates are for 2011–2017; those for peatland are not significantly different from zero. Details of all calculations in the table are in *Materials and Methods*.

Norway agreed to pay Indonesia US$56.2 million for the Indonesia–Norway partnership’s estimate of 11.2 MtCO_2_-eq of avoided emissions in 2017, including emissions from avoided peat fires and peat decomposition. Dividing this payment by our estimated average annual emissions reduction during the 2011–2017 period gives effective carbon price ranges of, respectively, US$4.3/tCO_2_-eq to US$5.4/tCO_2_-eq and US$1.8/ tCO_2_-eq to US$2.3/tCO_2_-eq, with and without the share of the payment for avoided peat fires and decomposition. All of these estimates are within range of Norway’s proposed carbon price, US$5/tCO_2_-eq. Applied to our estimates of cumulative emissions reductions over the entire 2011–2017 period, Norway’s payment has effectively bought emissions reductions at less than US$1/tCO_2_-eq, thus representing value for money from Norway’s perspective. Indeed, from a global perspective, these are very cost-effective emissions reductions.

## Discussion

### Impacts of Indonesia’s Moratorium on Forest Loss and Emissions.

The centerpiece of one of Norway’s pioneering REDD+ partnerships, Indonesia’s moratorium and the associated US$1 billion pledge, represented an ambitious scaling up of tropical forest conservation efforts. We found a relatively small effect of the moratorium in slowing deforestation. Our cumulative estimates to 2018, using a quasi-experimental program evaluation approach, are at the lower bound of estimates in previous research (27). We also found evidence of a positive impact on dryland forest that materialized earlier, between 2012 and 2016 (Fig. 2), a period that has not been assessed for emissions reductions by the Indonesia–Norway partnership. The magnitude of our estimated impacts, and our regression discontinuity results, suggests either that moratorium areas were mostly economically marginal and, compared to matched control areas, unlikely to experience a large effect from the moratorium, or that deforestation has continued largely unchecked by the moratorium. The general secular decline in forest cover in all areas (Fig. 1) suggests the latter explanation is more likely.

What matters for REDD+ is how these positive impacts on forest cover translate into carbon emissions reductions. Our estimates in Table 1 are in line with a projection that the moratorium had the potential to cumulatively reduce emissions by nearly 200 MtCO_2_-eq by 2030 (44), at an estimated annual average of 9.4 MtCO_2_-eq. Note, however, that our impact estimates stem from differences between two declining paths of forest cover over time. While straightforward to measure against a BAU target, this just delays emissions from deforestation. To stop emissions permanently, deforestation needs to be halted, not slowed. With this in mind, our estimates accounted for, at most, around 13% and 4% of Indonesia’s NDC (unconditional) commitment to reduce GHG emissions by 29% in 2020 and 2030, respectively. Our estimates suggest that Indonesia is unlikely to meet this commitment given that most of it (17.2 percentage points, or three-fifths of the 29% target) is supposed to be met via the country’s forest sector (4). Peatland forest loss and peat fires are key contributors to Indonesia’s share of global, forest-based emissions, yet our results suggest that the moratorium has had no meaningful impact on peatland forests. Norway’s proposed payment of US$56.2 million included emissions reductions from avoided peat decomposition and fires in 2017. Our peatland results imply that, viewed purely in terms of performance, this share of the payment could be justifiably withheld.

### Comparing Estimates of Impact.

That our estimates of impact differ from those calculated by the Indonesia–Norway partnership is primarily due to the choice of baseline, or counterfactual, against which impact was measured. For emissions reductions between 2018 and 2020, the Indonesia–Norway partnership planned to adopt a historical baseline similar to the one the partnership used for estimating reductions in 2017 (28). How this baseline is estimated originates from the calculation of Indonesia’s arguably more generous forest reference emission level (FREL). Submitted to the United Nations Framework Convention on Climate Change in 2016, the FREL is based on the average annual deforestation rate between 1990 and 2012 (4, 45). Indonesia’s FREL provided the basis for a proposed US$103.8 million payment from the Green Climate Fund (GCF) for an estimated 20.3 MtCO_2_-eq reduction in carbon emissions between 2014 and 2016 (46).

Although both the Norwegian and GCF payments are supposed to be “results based,” the baselines used to estimate these emissions reductions emerged as a consequence of political negotiations and are subject to precisely the biases that we attempted to eliminate in our program evaluation approach. Thus, they are arguably independent of performance (47); that is, the counterfactuals constructed by the Indonesia–Norway partnership and the GCF could be driven entirely by stochastic shocks and economic factors unrelated to REDD+ efforts. Our causal framework explicitly attempted to balance these biases. On the basis of historical performance, our results suggest that Indonesia could legitimately claim an even larger payment from Norway, up to US$339 million to US$434.5 million on the basis of a US$5/tCO_2_-eq carbon price, for cumulative emissions reductions between 2011 and 2018. Fig. 2 shows a predominantly steady cumulative effect of the moratorium over time, particularly from 2016 onward. The moratorium seems to have had a causal impact on avoided deforestation.

### Beyond the Moratorium.

The partnership underlying the moratorium was unilaterally terminated by Indonesia in 2021, apparently due, in part, to delays in release of the payment by Norway (16). Even though this implies that the moratorium is unlikely to continue, at least not in its current form, large-scale, area-based initiatives, in the form of jurisdictional REDD+ schemes, are likely in the future. For example, the Lowering Emissions by Accelerating Forest finance (LEAF) private–public coalition was established in 2021 to mobilize at least US$1 billion for area-based tropical forest conservation (46). Such initiatives could usefully learn from how the moratorium performed with respect to emissions reductions.

First, the small size of the moratorium’s impact suggests limited compliance. Improving compliance might increase impact, yet patronage linkages, between large-scale industrial plantation companies and local politicians, ensured weak monitoring and enforcement (48). Future REDD+ initiatives could help bolster local monitoring and enforcement capabilities. The ongoing One Map process, to resolve inconsistencies resulting from the use of different data and maps by creating a national standard of land cover and usage, could further help strengthen transparency and improve forest governance (11).

Second, future initiatives could also help incentivize reductions in emissions from deforestation by local forest users not originally targeted by the moratorium, such as smallholders engaged in logging and palm oil production (49, 50), who reportedly contributed one-fifth of nationwide forest loss between 2001 and 2016 (10) The forestland claims of local forest users have been strengthened by the Village Law (Law No. 6/2014) (51), which, combined with the millions of hectares of forest pledged for “social forestry” initiatives, suggests new conservation opportunities (52). These opportunities could be aligned with the goals of future initiatives, although there remains a risk that continued policy layering could exacerbate ambiguity with respect to forest regulations and enforcement (53). Also, given ongoing uncertainties over forest users’ land rights, new initiatives should pay careful attention to representation and recognition notions of justice as a means of legitimizing REDD+ at the local scale (54).

Third, the moratorium lacked formal allocation and distributional mechanisms. Our results are based on the aggregate ATT, but the district-level ATT indicates where the positive effects were likely generated (*SI Appendix*, Fig. S5). Consistent with calls from forest-rich districts for an “ecological fiscal transfer” scheme based on ecological performance (55), payments from future initiatives could be distributed to districts that demonstrate emissions reductions. The Regional Governance Law (Law No. 23/2014), however, shifted control over forests from district to provincial governments and established a greater administrative role for forest management units (56). Thus, both provincial and district governments are likely to play a role in any benefits transfer system, perhaps via intergovernmental fiscal transfers (IFT) (57). Presently focused on timber production revenues, Indonesia’s forest sector IFT could, in theory, be used to transfer REDD+ funds (58).

### Delivering Carbon Value for Money and Meeting the NDC Commitments.

The value of estimated cumulative emissions reductions, even on the basis of a relatively low carbon price of US$5/tCO_2_-eq, comfortably exceeds the amount Norway agreed to pay for emissions reductions in 2017. From the perspective of Norway’s government, and the global community, Norway’s payment could be characterized in terms of abatement costs, that is, the sum that Indonesia’s government is willing to accept (WTA) to reduce emissions from deforestation. However, the global benefit, and in principle the willingness to pay (WTP) for emissions reductions, is the social cost of carbon (SCC). The SCC is estimated to range between approximately US$40/tCO_2_-eq and US$200/tCO_2_-eq (e.g., ref. 59). The question of how the discrepancy between WTP and WTA is shared between donor and recipient countries was resolved by the Indonesia–Norway partnership, moving far closer to the WTA than to the WTP. Thus, Norway, and indeed the global community, would be getting “good value” for emissions reductions in Indonesia. The moratorium appeared to be cost-effective but with a very skewed share of the global surplus transferred to a carbon-rich yet poor country. Although Indonesia accepted the US$5/tCO_2_-eq price offered by Norway, this is arguably an unfair distribution of the surplus, given estimates of the SCC. With the ending of the Indonesia–Norway partnership, Indonesia could negotiate a higher price with, for example, an initiative like LEAF, which is offering a minimum of US$10/ tCO_2_-eq (60).

Our analysis emphasizes that emissions reductions, although cost-effective, still require large transfers, even at low carbon prices. Much steeper emissions reductions are clearly needed to reach the NDC targets, but the cost of these reductions is unlikely to be met by a single country or initiative. Indeed, Indonesia estimates that, to meet its NDC commitments, around US$5.5 billion is required between 2018 and 2030 for the country’s forest sector alone (4). When REDD+ first emerged in the 2000s, there were initial calls for US$10 billion to US$15 billion of funding per year to cut global deforestation by half (61). These funding needs were based on opportunity cost calculations, which will be higher for high-value agricultural commodities such as palm oil. Yet, by the 2010s, pledges to the value of only US$10 billion had been made for REDD+ (62).

It was hoped that a global climate agreement, incorporating a cap-and-trade system, would generate sufficient and sustainable sources of finance for the protection of tropical forest carbon stocks. Given that such a system has yet to materialize, it has fallen upon individual countries to voluntarily finance REDD+ initiatives around the world. Norway’s contribution, to date, exceeds that of all other countries but is insufficient to protect tropical forest carbon stocks at a scale necessary to meaningfully contribute to global climate change mitigation efforts. As the world’s attention moves beyond COP26 in Glasgow, where an ambitious global commitment was made to halt deforestation by 2030, the critical climate role of forests needs to be matched by a global willingness to pay for it.

## Materials and Methods

### Data.

Our outcome variable is forest cover in hectares, with data spanning the period 2000–2018 drawn from the Global Forest Change dataset (38). The 2004–2018 period is selected for matching data in our analysis, while data for the 2000–2004 period are used in separate placebo tests that determine whether there are violations in the parallel trends assumption (see below). The forest cover data, obtained for the whole of Indonesia, are spatially explicit and not defined according to different forest classes. Our units of analysis are grid cells of 1.2 km by 1.2 km (144 ha), which accommodate 1,600 pixels at a resolution of 30 m by 30 m (i.e., Landsat8 pixel size). A scale of 1.2 km by 1.2 km is chosen because it allows for a similar scale across the different sources of data used in our analysis and minimizes the risk of grid cells overlapping treatment and control areas. For each grid cell and for each year of our study period, we count the number of pixels where forest loss is recorded and then convert pixels to hectares by multiplying by 0.09. We account for the precise fraction of a pixel that falls within a grid cell.

Tree cover in the year 2000, the base year of the Global Forest Change dataset (38), is defined as canopy closure for all vegetation taller than 5 m in height, and is encoded as a percentage per pixel, in the range 0 to 100. Forest loss during the period 2001–2018 is defined as a stand-replacement disturbance, or a change from a forest to nonforest state at the pixel scale, encoded as either 0 (no loss) or else a value in the range 1 to 18, representing loss detected in the year 2001–2018, respectively. A pixel is categorized as forested if its canopy cover is greater or equal to 25%, below which a pixel changes its state from forest to nonforest (63). We obtain peat depth data from ref. 64, which are used to subdivide grid cells into peatland and dryland forest types. These two types are analyzed separately due to their different ecological characteristics that are relevant to changes in forest cover and carbon emissions.

Forest areas covered by the moratorium supposedly included all of Indonesia’s primary and peatland forests, and are determined using moratorium shapefiles obtained from ref. 65. Our treatment group includes forest areas within the moratorium boundaries that were established in 2011 (*SI Appendix*, Fig. S2). Since 2011, the moratorium’s boundaries have shifted due to forestland being redesignated by Indonesia’s district governments and dropped out of the moratorium before typically being licensed out to concessionaires (22, 66, 67) (*SI Appendix*, section 1). Although legal, this redesignation of forestland is effectively a behavioral response to the moratorium and, hence, should be included in estimates of impact. Thus, the 2011 moratorium boundaries are assumed constant throughout our treatment periods. Shapefiles for the location of palm oil, timber, and logging concessions established before the start of the moratorium were originally obtained from ref. 68. Digitized by Greenpeace, this was the most comprehensive source of concessions data available.

We compile a vector of control variables and time-invariant characteristics at the grid cell level by combining different sources of georeferenced data: information on altitude, slope, and distance from major roads from the WorldPop repository (69); grid cell–level travel time to major cities (70); and a population trend based on counts for the years 2004 and 2010 (71). Grid cell–level aboveground carbon stock values are estimated by dividing data on aboveground biomass density, from the Global Forest Watch dataset, by 0.5 (based on ref. 72).

The cumulative impacts of the moratorium on forest cover and carbon emissions are estimated each year in the periods 2011–2018 and 2011–2017 for dryland and peatland forest, respectively. The latter is estimated only up until 2017, due to the additional restrictions on peatland forest conversion implemented in 2017 (12).

### Empirical Approach.

The moratorium mandated that district governments stop issuing new concession licenses in forest areas covered by the moratorium. Evaluating the causal impact of the moratorium is complicated by selection bias: Forest areas covered by the moratorium differ in their observable and unobservable characteristics. Any imbalance in these characteristics implies that simply comparing the extent of forest cover in the moratorium and nonmoratorium areas will capture preexisting imbalances in, for example, their suitability for palm oil or timber production, thus confounding the estimate of the treatment effect. Descriptive statistics illustrate the differences between the moratorium and nonmoratorium forest areas (*SI Appendix*, Tables S27–S34). The values of several variables that determine the suitability of grid cells for new concessions differ between moratorium and nonmoratorium areas. For example, in the unmatched data, moratorium grid cells have, on average, a higher elevation and are farther away from roads and cities than nonmoratorium cells, making them less suitable for concessions, other things being equal. It is necessary to address the imbalance of these observable characteristics to estimate the causal effect of the moratorium on forest cover.

We adopt a DD research design to control for observable and unobservable confounding characteristics in the estimation of the treatment effect. Empirical testing leads us to prefer a matched DDD estimator. Via matching moratorium and nonmoratorium cells on the basis of their observable characteristics, we argue that the confounding effect of unobservable characteristics is also controlled for, thus generating an unbiased estimate of the treatment effect (73–75). Prior to estimation, we adjust the moratorium and nonmoratorium samples by excluding cells which are unlikely to become concessions for agronomic or jurisdictional reasons. We first exclude cells which are part of the Indonesian protected area network, both within and outside the moratorium, as conversion in these cells is already strictly prohibited. Second, we focus on unconverted dryland and peatland forest outside of concessions. We then remove all cells outside of concessions with an elevation of 1,000 m or more above sea level. The likelihood of these cells being a realistic proposition for a concession in either moratorium or nonmoratorium areas is close to zero because, above 1,000 m, land is unsuitable for palm oil cultivation (76) and for *Acacia mangium*, the main tree species employed for the production of wood pulp and paper (77). These adjustments represent our first attempt to balance the sample in terms of the likelihood of concessions being granted. The resulting dataset has 567,634 cells, of which there are 160,012 treated observations (28.2%). Next, we deploy a matching procedure to match individual grid cells in moratorium areas with counterfactual grid cells in nonmoratorium areas, and vice versa. Our main results use propensity score, one-to-one caliper matching for this purpose. After matching, we retain 152,118 treated cells (7,894 dropped cells), and 198,794 control cells, for a total of 358,806 cells. The dropped cells represent a reduction of 2.2% (4.9% of the treated group) due to the exclusion of imprecise (outside of the caliper) matches. This matched dataset is used for both parametric and nonparametric estimators, to ensure balanced characteristics between the treatment and control groups and to facilitate easier comparisons between estimators (73). When analyzing the moratorium’s impact on different forest types (dryland, peatland), the propensity score is estimated separately, and different matched samples arise.

Having balanced the sample in this way, the identification of a causal estimate of the moratorium’s impact on forest cover stems from a DD research design. It is well known that, under their identifying assumptions, DD estimators identify the ATT (e.g., ref. 78, chap. 4), which can be understood as the impact of the moratorium on forest areas covered by the moratorium and hence, is a policy-relevant treatment effect. With the dropping of cells, we are no longer identifying the ATT, although dropping fewer than 5% of treated observations arguably results in a close approximation to the ATT. Where *d_i_* indicates whether an individual grid cell *i* is in the treatment group (= 1) or not (= 0), and $Y_{1iT_{1}}$ and $Y_{0iT_{1}}$ are, respectively, the potential outcomes (forest cover) in the treated (= 1) and untreated (= 0) states for grid cell *i* at posttreatment time *T*_1_, the ATT is defined as

[1] $$\mathrm{ATT}=E\left[Y_{1iT_{1}}-Y_{0iT_{1}}|d_{i}=1\right].$$

Following ref. 78 (p. 101), if the potential outcomes have the separable form $Y_{\mathrm{kit}}=\mu_{\mathrm{kit}}+\lambda_{i}+u_{\mathrm{kit}}$ for treatment states *k* = 0, 1, and individual, grid-level fixed effect *λ_i_*, the ATT is identified by a DD estimator of the form

[2] $$DD=E\left[\Delta_{T_{1},T_{0}}Y_{it}|d_{i}=1\right]-E\left[\Delta_{T_{1},T_{0}}Y_{it}|d_{i}=0\right],$$

where *Y_it_* is the observed data and $\Delta_{T_{1},T_{0}}$ is the change operator between the pretreatment period *T*_0_ and *T*_1_ (see also *SI Appendix*, section 4). The DD estimator controls for individual fixed effects *λ_i_* by taking differences at the individual level. A necessary condition to identify the ATT in this way is the parallel trends assumption,

[3] $$E[\Delta_{T_{1},T_{0}}u_{1it}|d_{i}=1]-E\left[\Delta_{T_{1},T_{0}}u_{0it}|d_{i}=0\right]=0,$$

meaning that the unobservable characteristics determining forest cover must be identical in expectation; otherwise, they will confound the estimate of impact.

The DD estimator in Eq. **2** can be estimated parametrically or nonparametrically (e.g., matching). The parametric DD estimator we use takes the following form, and is estimated using a fixed-effects estimator:

[4] $$Y_{it}=\alpha +\sum_{s=\tau }^{T}\beta_{1s}D_{\mathrm{sit}}+\sum_{k=2}^{n}\beta_{k}X_{it}+\lambda_{i}+\theta_{t}+\varepsilon_{it},$$

where *Y_it_* is forest cover in (nonconcession) grid cell *i* in year *t*, *D_it_* is the time-varying moratorium treatment indicator, and *X_kit_* are *n* potential time-varying control variables. The $\beta_{1s}$ coefficients represent the DD estimates of ATT for each posttreatment year *s* between 2012 and 2018. This basic model controls for time-invariant characteristics via the individual, grid-level fixed effects, *λ_i_*, and time fixed effects, *θ_t_*, which capture shocks common to all grid cells, such as weather shocks.

We select among a number of different parametric and nonparametric (matched) DD and DDD estimators through a four-step process of model selection. In the first step, we estimate parametric DD models of the form described in Eq. **4**, including district-by-year trends, pretreatment forest cover-by-year interactions, and both clustered (at the district level) and Conley SEs (this accounts for spatial autocorrelation using the “fixest” package in R), but no further control variables (*X_kit_*), before comparing these estimates to a propensity score, one-to-one caliper, matched DD estimator (*SI Appendix*, Table S35). Clustering and Conley SEs do not affect the results. To rule out the possibility that the balance in the sample of observable characteristics between moratorium and nonmoratorium areas causes differences between the parametric and nonparametric estimates, we use the same matched sample for the parametric DD estimation as is used for matched DD estimators (see ref. 73). We then undertake sensitivity analysis on the nonparametric estimators by relaxing the precision of the matching procedure in two ways: 1) widening the caliper and 2) sampling matches without replacement. The sensitivity analysis suggests that the matching estimates are sensitive only to extreme reductions in precision of the matching (no replacement or no caliper) (*SI Appendix*, Table S35). Extreme sensitivity of the parametric estimator to the inclusion of district-by-year trends suggests that heterogeneity across grid cells is a potentially important confounding factor. Matching estimators deal more flexibly with heterogeneity, and, in matched DD estimators, this can include heterogeneous trends (79). There is also empirical evidence to show that the parallel trends assumption is more likely to hold with matched DD rather than parametric DD (e.g., ref. 80). To account for heterogeneous trends, our matching procedure matches moratorium and nonmoratorium grid cells very precisely, and explicitly, on pretreatment trends. Given the sensitivity of the parametric estimator and the fact that matching estimators are better equipped to deal with heterogeneity, we opt for a nonparametric DD approach, among which we include propensity score matched DD.

Our central estimates use propensity score, one-to-one caliper matching. The matching variables we use capture important differences between the moratorium and nonmoratorium grid cells, their dynamics, and suitability for future concessions. We use pretreatment values of distance to concessions (palm oil, timber, and logging), distance to roads and cities, population (2005 and 2010), forest cover for each year from 2005 to 2010, elevation, slope, peat depth, and aboveground carbon stock in the year 2000. Matching on pretreatment outcomes (forest cover) and population in more than one pretreatment year (2005–2010 for forest cover, 2005 and 2010 for population) attempts to control for heterogeneous pretreatment trends and levels between moratorium and nonmoratorium areas.

The matched DD estimator takes the following form and estimates $ATT_{DD,T}$ for time horizon *T* using forest cover data $Y_{it}^{M}$ from the moratorium grid cell *i* matched with data $Y_{it}^{j,NM}$ in cell *j* from the nonmoratorium grid cells:

[5] $${\hat{\mathrm{ATT}}}_{DD,T}=\frac{1}{N_{M}}\sum_{i}I_{0}\left[\left[Y_{i,T}^{M}-Y_{i,T_{1}}^{M}\right]-\left[Y_{i,T}^{j,NM}-Y_{i,T_{1}}^{j,NM}\right]\right],$$

where *I*_0_ is an indicator variable that is equal to one if a grid cell *i* in the moratorium has a counterfactual grid cell *j* in the nonmoratorium area whose propensity scores *p_i_* and *p_j_* fall within the caliper,

$$|p_{i}-p_{j}|<\epsilon ,$$

where *ϵ* is a predetermined distance in propensity score space. The caliper defines the set of one-to-one matches from the nonmoratorium area, C(i), such that j∈C(i). Therefore, $I_{0}=I\left(\min|p_{i}-p_{j}|:|p_{i}-p_{j}|<\epsilon \right)$. Our large sample allows us to choose a very precise caliper (0.0001) without substantially reducing the sample size.

In the second step, we subject this matched DD estimator to two separate placebo (falsification) tests in time, both of which model a placebo moratorium implemented in 2004. Placebo test 1 retains the matches from the real pretreatment phase (2005–2010), while placebo test 2 reestimates the propensity scores for the pre–placebo treatment phase (2000–2004) (*SI Appendix*, Tables S36–S39 and Fig. S6). In some cases, the null hypothesis is rejected for the matched DD estimator, suggesting a failure of the parallel trends assumption. For this reason, in the third step, we use a matched DDD estimator inspired by refs. 37 and 40, which applies a correction for nonparallel trends. The DDD estimator takes the following form:

[6] $$\begin{array}{l} {\hat{\mathrm{ATT}}}_{\mathrm{DDD},T}=\;\frac{1}{N_{M}}\sum_{i}I_{0}\{\left[\left[Y_{i,T}^{M}-Y_{i,T_{1}}^{M}\right]-\left[Y_{i,T}^{j,NM}-Y_{i,T_{1}}^{j,NM}\right]\right]\\ -\left[\left[Y_{i,T_{1}\prime }^{M}-Y_{i,T_{0}}^{M}\right]-\left[Y_{i,T_{1}\prime }^{j,NM}-Y_{i,T_{0}}^{j,NM}\right]\right]\left(\frac{T-T_{1}}{T_{1}\prime -T_{0}}\right)\}. \end{array}$$

The second line reflects the correction for the nonparallel pretreatment trends between *T*_0_ and $T_{1}\prime$ with a correction $\left(T-T_{1}/T_{1}\prime -T_{0}\right)$ to adjust the trend correction for potentially different pretreatment and posttreatment time horizons. The matched DD results come from the estimator in Eq. **5**, and the matched DDD results come from the estimator in Eq. **6**. Finally, we subject the DDD estimator to a placebo in time test of the parallel trends assumption, placebo test 3 (*SI Appendix*, Table S40). The years used to estimate Eqs. **5** and **6** are $T_{0}=2004,\;T_{1}\prime =2010,\;T_{1}=2011$ and T= endpoint year. For the placebo tests, the placebo treatment year is 2005, and the pretreatment and posttreatment periods considered are, respectively, 2000–2004 and 2005–2010, with sensitivity using 2005–2011. In each case, we use the Matching routine in R (81).

Finally, in step four, we undertake a spatial placebo test to evaluate the robustness of the matched DDD estimator. The spatial placebo test uses the DDD estimator in Eq. **6** and applies it to concession moratorium and concession nonmoratorium grid cells for dryland and peatland forest. In theory, the moratorium has no effect on forest cover in concession grid cells because conversion is still allowed on concessions in moratorium areas that were granted premoratorium. This observation forms the basis of the null hypothesis, with the alternative hypothesis that moratorium and nonmoratorium concession forest cover is evolving in different ways, hence falsifying the parallel trends assumption. Results of the spatial placebo test suggest support for the null hypothesis (*SI Appendix*, Tables S41 and S42).

The resulting, preferred estimator is the propensity score, one-to-one caliper matched DDD estimator. Covariate balance tables are reported for all specifications (*SI Appendix*, Tables S43–S54). SEs are calculated according to the consistent Abadie–Imbens procedure for matching (82).

We undertake several robustness checks on our estimates in Fig. 2. These estimates are based on untrimmed samples, so we first obtain estimates of the ATT for a sample with a stricter definition of forest cover at the grid cell level: 30% (or more) and 60% (or more) forested pixels in a grid cell, in 2005. These samples are obtained by estimating the mean forest cover of 2005 pixels in the grid cells. Second, we conduct a robustness analysis on alternative approaches to matching which do not rely on the propensity score, namely CEM (83, 84). CEM addresses the possibility that propensity score matching may introduce biases due to the way in which it reduces the dimensionality of the matching problem to matching on a single dimension: the propensity score (e.g., ref. 85). We undertake CEM using the same matching variables as before. As with propensity score matching, sample sizes are also sensitive to choices of matching variables, the coarseness of matching, and other implementation decisions in CEM. For this reason, following refs. 84 and 86, we undertake four separate CEM routines in which the control variables are used either in the exact matching algorithm or as control variables in a covariate adjustment step after matching has occurred (84). The DDD CEM covariate-adjusted estimate of ATT over the period of the moratorium from period *T*_1_ and *T* is the estimate of *β*_1_ in the following:

[7] $$\Delta_{T_{1},T}^{\mathrm{DDD}}Y_{i}=\alpha +\beta_{1}d_{i}+\sum_{k=2}^{n}\beta_{k}X_{ki}+\epsilon_{i}.$$

The *X_ki_* are the pretreatment control variables that provide covariate adjustment, which we exclude from the matching algorithm. $\Delta_{T_{1},T}^{\mathrm{DDD}}Y_{i}$ is the individual-level matched triple-difference (corrected for nonparallel trends) in forest cover that is constructed by the CEM matching algorithm. We use the cem routine in R to undertake the matching, and the att routine in R to obtain this covariate-adjusted estimate of the ATT.

Third, we conduct sensitivity analyses on the assumptions used in the central nonparametric DDD propensity score matching estimates. These analyses focus on 1) caliper width choices, where calipers of 0.01 and 0.001 are used in one-to-one nearest neighbor matching, and 2) *k* > 1 nearest-neighbors analysis, in which we test the sensitivity of our results to matching on *k* = 2, 3, 5 nearest neighbors instead of one to one. Fourth, we test the sensitivity of the sample being limited to grid cells below an elevation of 1,000 m. We estimate separate ATT for 1) cells at all elevations and 2) cells only found above 1,000 m (covariate balancing reported in *SI Appendix*, Tables S55–S58). Finally, we replicate the analysis in Fig. 2 using a tighter definition of forest, specifically, “intact primary” forest, with no detectable signs of human-caused alteration or fragmentation (41, 42) (covariate balancing reported in *SI Appendix*, Tables S59 and S60).

A final identification assumption of the DD and DDD estimators is the stable unit treatment value assumption: The treatment should not cause leakage to untreated forest areas. Such leakage/spillover effects are a common confounder in the evaluation of area-based policies (e.g., refs. 32, 33, 87, and 88). If protection via the moratorium induces the displacement of forest clearing to outside the moratorium’s boundaries, deforestation rates inside and outside these boundaries are contemporaneously affected in opposite directions, resulting in treatment effects of a higher magnitude than the “true” effects. To check for leakage, we use a regression discontinuity design (RDD) (43, 89) with a sharp cutoff at the moratorium’s boundaries. RDD estimates the LATE of the moratorium in the proximity of its boundaries with nonmoratorium land.

We specify separate linear polynomials on both sides of the boundary cutoff following (90), and estimate treatment effects via ordinary least squares regressions with robust SEs clustered at the district administrative level. Our preferred results are obtained via separate linear polynomials and optimal bandwidth selection (through the ref. 43 method). We also examine an alternative bandwidth selection algorithm (91), additional fixed bandwidths of 5, 10, and 20 km from the moratorium’s boundaries, and specifications using separate quadratic polynomials of distance (*SI Appendix*, section 4 and Figs. S7–S13). Given the spatial configuration of the moratorium (*SI Appendix*, Fig. S2), our bandwidths cover a wide extent of nonmoratorium cells in which leakage could feasibly occur.

### Estimating Carbon Emissions.

We obtain grid cell–level CO_2_-eq estimates by multiplying the average carbon stock (in tons of carbon per hectare) per grid cell by our ATT estimates of avoided deforestation (hectares). These results are then extrapolated to the total number of moratorium grid cells in the sample, and converted to tons of carbon equivalent, tCO_2_-eq, by applying a conversion factor of 3.67 (92). Belowground carbon stocks in Table 1 are approximated by multiplying aboveground carbon by 1.24 following ref. 27.

In Table 1, total emissions of 2.2 GtCO_2_-eq per y are based on the projected BAU emissions for all sectors excluding Land Use, Land-Use Change and Forestry (LULUCF) plus estimated annual emissions from LULUCF (1.0 GtCO_2_-eq per y) according to ref. 4 and reported by Climate Action Tracker. The estimate of 1.0 GtCO_2_-eq per y is an annual average over 2001–2018. Indonesia’s NDC commitments involve an emissions reduction path which implies specific reductions against the BAU in 2020: an unconditional (without overseas assistance) 29% reduction in emissions compared to projected BAU emissions in 2030, and a 41% conditional (with overseas assistance) emissions reduction.

We measure our estimated avoided deforestation against the 2030 commitment’s implied emissions reduction for 2020. The BAU emissions (non-LULUCF) in 2020 are projected to be 1.2 GtCO_2_-eq. The conditional (unconditional) emissions reduction in 2020 represents an 8% (11%) reduction against 2020 BAU emissions. The 2030 29% conditional (unconditional) NDC implies a target of 1.12 (1.09) GtCO_2_-eq in 2020, a reduction of 100 (132.9) MtCO_2_-eq. The impact of the moratorium is calculated as a percentage of the 100 (132.9) MtCO_2_-eq reduction. BAU (non-LULUCF) and unconditional (conditional) pledged emissions in 2030 are 2.2 GtCO_2_-eq and 1.8 (1.6) GtCO_2_-eq, implying a reduction of 328 (527) MtCO_2_-eq in 2030. Our estimated moratorium impact is calculated as a percentage of these figures.

Norway’s agreed payment of US$56.2 million includes emissions reductions from peatland forest in 2017. Without this, the payment falls to US$37 million net of the 35% “set-aside factor” (*SI Appendix*, section 1).

## Supplementary Material

Supplementary File

## Data Availability

All replication materials, including processed datasets, regression routines, and R scripts used to generate our results, have been deposited in Harvard Dataverse (https://doi.org/10.7910/DVN/0EUW82). Previously published data were used for this work (38, 64, 65, 69–72, 93).
