# Supplementary material for: Carbon emissions reductions from Indonesia’s moratorium on forest concessions are cost-effective yet contribute little to Paris pledges
Source: Proc Natl Acad Sci U S A. 2022 Jan 24;119(5):e2102613119. doi: 10.1073/pnas.2102613119 (PMC8812685; doi:10.1073/pnas.2102613119)
Supplement: Supplementary File [file pnas.2102613119.sapp.pdf]

PNAS
www.pnas.org

Supplementary Information for

Carbon emissions reductions from Indonesia's Moratorium on forest concessions are cost-effective yet contribute little to Paris pledges

Ben Groom, Charles Palmer and Lorenzo Sileci

Charles Palmer.
E-mail: c.palmer1@lse.ac.uk

This PDF file includes:

Supplementary text
Figs. S1 to S13
Tables S1 to S60
SI References

Supporting Information Text

1. Background to the Moratorium

The Indonesia-Norway REDD+ partnership. On 26 May 2010, a Letter of Intent (LoI) was signed by the governments of Indonesia and Norway, establishing the Indonesia-Norway REDD+ partnership. As a part of this partnership, Norway pledged to provide US$1 billion to Indonesia in exchange for verified reductions in GHG emissions from deforestation. Three phases were specified in the LoI. The first aimed to establish the necessary institutions and capacity, the second to transform managerial systems while verified emission reductions were to be delivered in the third. Based on the Lol, Indonesia was supposed to be on track to receive its first result-based payment in 2014 but the transition to phase three was delayed due to various political and institutional changes in Indonesia (1).

To operationalise the three phases, various REDD+ Task Forces were appointed between 2010 and 2013 by the then President of Indonesia, Susilo Bambang Yudhoyono, alongside a ministerial-level REDD+ Management Agency (BP REDD+) that was created in 2013. In 2015, President Joko Widodo, who was elected in 2014, dissolved BP REDD+ and a new Ministry of Environment and Forestry (KLHK) was formed from the Ministry of Environment and the Ministry of Forestry. This new ministry absorbed the National Council on Climate Change (DNPI) and BP REDD+. Thus, the responsibility for delivering GHG emission reductions fell to KLHK, the capacity of which has been supported through cooperation with Kemitraan (the Partnership for Governance Reform in Indonesia) and the World Resources Institute Indonesia (WRI).

Further delaying Indonesia's transition into phase three was the long time that was needed to create an integrated measurement, reporting and verification (MRV) system sufficient to account for progress in reducing emissions from deforestation (2). As of 2018, there was no MRV system in place. A key reason for the long gestation of this system was the context and purpose of national MRV capacity, which has evolved since the LoI was signed in 2010. Specifically, a divergence arose between the commitment to economy-wide emission reductions in line with national policy, coordinated by the national planning agency (Bappenas), and emissions reductions commitments as part of Indonesia's Nationally Determined Contribution (NDC). The latter is led by KLHK but is coordinated with other agencies and sub-national entities that regulate or impact forest and peatland. In addition, there were and continue to be uncertainties regarding forest definitions, boundaries and baselines.

Similar to the MRV system, the Indonesia-Norway partnership has had to create and develop other, critical institutional arrangements off the back of relatively weak, pre-existing arrangements. The most-recent review of the partnership, published in 2018 (2), makes clear that forest law enforcement remains patchy at best. The partnership has been financing various capacity-building initiatives in KLHK. For example, it has been developing capacity to detect networks of individuals, holding companies and licensees who may have violated the Moratorium, to track prosecutions, recover assets and detect corruption. The partnership has also been involved in efforts to strengthen civil society capacity in the use of high technology and fieldwork to support independent monitoring and reporting on forests. To manage financial flows linked to REDD+, including the first payment announced in 2019 for emissions reductions in 2017 (see below), the Indonesian Environmental Estate Fund (BPDLH) has been established, overseen by the Ministry of Finance.

As of 2018, there has been relatively little progress on the implementation of sub-national REDD+ strategies and the institutional arrangements of the Indonesia-Norway REDD+ partnership remained highly centralised. Yet, at the local level there are various planning and mapping initiatives in, for example, Central, West and North Kalimantan, Aceh, South Sumatra and Riau. Central Kalimantan has a jurisdictional REDD+ initiative. There is, however, a lack of coordination of these sub-national efforts and little clarity on how REDD+ funds can be used in support of such efforts, as well as investment in the capacity of sub-national governments to, e.g. enforce the Moratorium.

In 2021, the Indonesian government formally and unexpectedly terminated its REDD+ partnership with Norway (3). One reason cited is a delay in the delivery of the payment from Norway, although there are also reports of disagreements between the two countries regarding the conditions required by Norway for the payment to be disbursed, e.g. (4).

The 2011 Moratorium on palm oil, timber and logging concessions. The centrepiece of the Indonesia-Norway REDD+ partnership was a Moratorium on new palm oil, timber and logging concession licences, instigated in 2011. Such licences were issued by district governments, the third administrative tier (after provincial governments), and conferred certain rights to firms. Palm oil concessions refer to areas where industrial-scale oil palm plantations are established. A logging concession refers to a forest area for managing and extracting timber, and are distinct from timber concessions, where plantation forests are established for the production of pulp and paper. After the timber has been extracted from logging concessions, many of these concessions are re-designated as palm oil or timber concessions (5). Fig. S1 shows the district-level distribution of all pre-2011 concessions, by share of the total area of the country's land area designated as concessions before 2011.

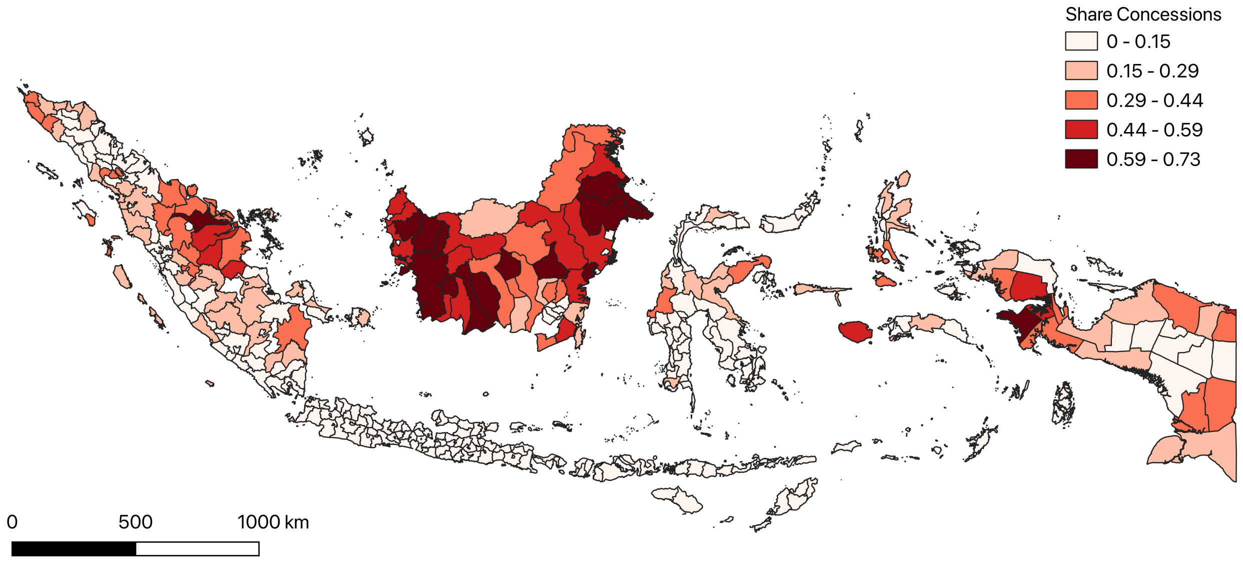

Fig. S1. Share of pre-2011 palm oil, timber and logging concessions (combined) by district.

Around 3% of land designated as concessions pre-2011 is located in forestland within the Moratorium's 2011 boundaries, which are shown in Fig. S2. Covering a substantial proportion of Indonesia's primary forest estate, the 2011 Moratorium was renewed every two years before being made permanent in 2019 (Presidential Instruction No. 10/2011, extended under No. 6/2013, then No 8/2015, then No. 6/2017 regarding a Moratorium on the Granting of New Licences and Improvement of Natural Primary Forest and Peatland Governance). The Moratorium boundaries established in 2011 have been periodically updated since. Initially covering 69 million hectares, the total area under the Moratorium has fluctuated since 2011, declining to about 64 million ha before rising to 66 million by 2018. These fluctuations were due to new spatial mapping inputs and surveys that changed the classification or designation of land (2).

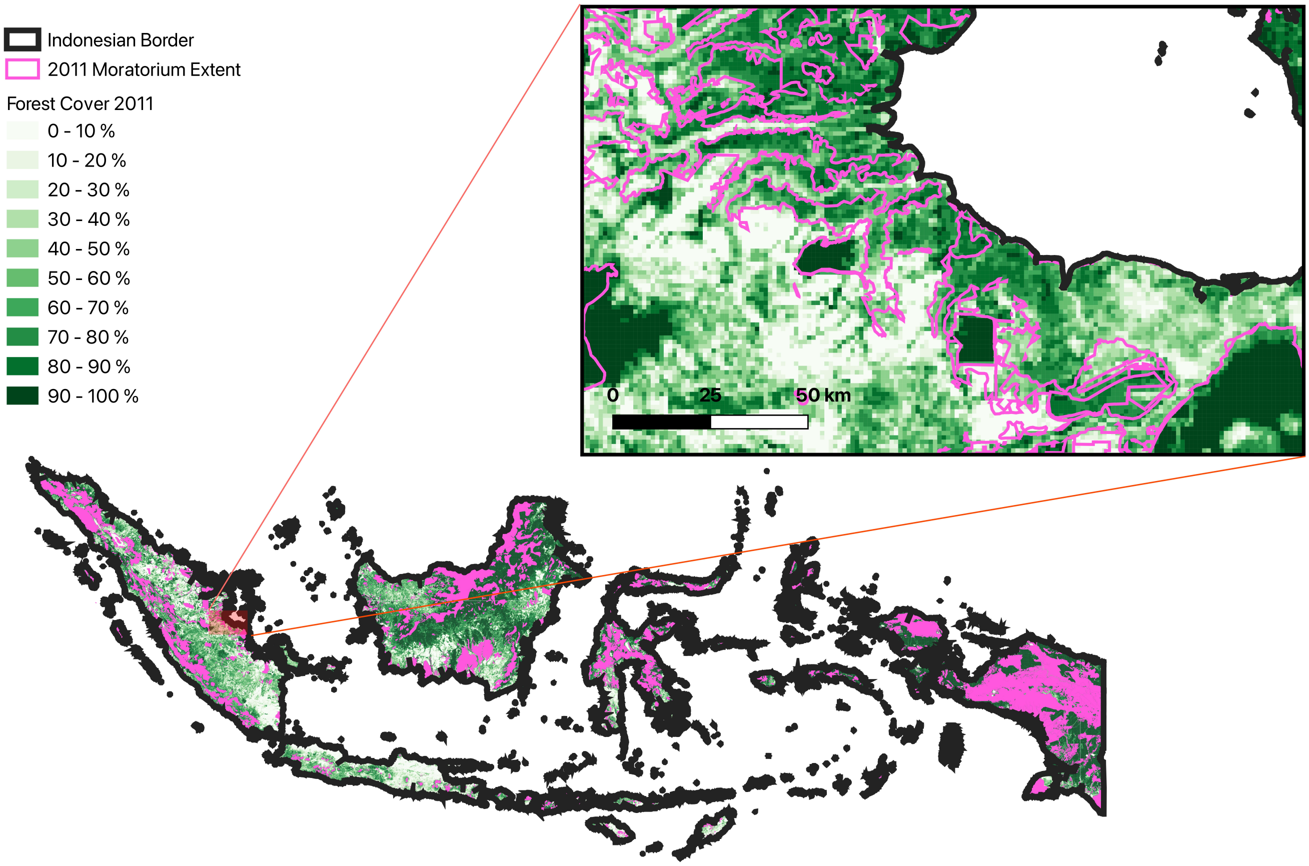

Fig. S2. Extent of Moratorium in Indonesia, 2011. The 2011 Moratorium boundaries denote the treatment areas for our empirical analysis, and are assumed constant throughout our treatment periods. Sources: Authors' elaboration on forest cover data (6). Moratorium shapefile from (7).

Some of the fluctuations in the Moratorium's boundaries were likely to have occurred due to changes in land classification that removed forestland from Moratorium areas to the benefit of concessionaires seeking new concession licences. Although the Moratorium is administered by the national government, the Moratorium map is revised by district governments, which then notify the national government of any changes (8). Once approved, areas of forest protected by the Moratorium are, in effect, dropped from the map. In particular, it has been documented that forestland has been re-designated, by district governments, from 'official forest' to 'non-institutionally recognized' forest, or land with forest cover that is designated as land 'for other use' (*Areal Penggunaan Lain*, APL) (9, 10). Areas of forest re-designated as APL have been shown to be highly susceptible to higher deforestation and degradation pressures (11), because designation as APL land makes it easier to obtain concession licenses in such areas (10).

According to the FAO, the total amount of APL forest land was around 8.5-8.6 million ha in 2009 and 2012 (12). It is difficult to precisely assess the extent to which the stock of APL forest land changed due to the addition of newly-designated APL land net of APL land being re-designated as new concessions, although Greenpeace has reported that, on the basis of an analysis of satellite imagery, up to 4.5 million ha of forest have been removed from the 2011 Moratorium map since 2011, of which at least 1.6 million ha was subsequently licenced out to concessionaires (8).

Concession area data, collected by Greenpeace circa 2010 and reported in (13), suggest that logging, timber and palm oil concessions accounted for approximately 24, 11 and 12 million ha of Indonesia's land, respectively. When land is allocated to a concessionaire, it is important to distinguish between the area of land designated as a concession and the area of land that has been cleared and, in the context of palm oil and timber concessions, planted. Indonesian government data, reported by Forest Watch Indonesia to the European Union in 2015 (5), suggests that total timber concession areas increased from around 8-9 million ha in 2009-10 to around 10 million ha in 2011-13, of which 50% was planted. Palm oil concession areas also increased, from around 8 million ha in 2009-10 to 9-10 million ha in 2011-12, before apparently more than doubling to over 22 million ha by 2018 (14). How much of this area was planted was unreported. Forest clearance in concessions has been well-documented, accounting for around half of Indonesia's aggregate forest loss (13, 15).

In our empirical analysis, we retain the 2011 Moratorium boundaries for estimation of the policy treatment effect and ignore subsequent changes to these boundaries because of evidence that the Moratorium may have driven incentives to re-designate land protected by the Moratorium into APL land before being re-designated as concessions. Although not technically breaking the rules of the Moratorium, we argue that this behaviour was not in the spirit of the Moratorium and indeed occurred in response to the Moratorium. Thus, any cells containing forestland within these 2011 boundaries that dropped out of the Moratorium map after 2011 are retained in our treatment group.

Our treatment period, on the other hand, reflects changes in the sectors affected by the Moratorium. Forest fires during the El Niño of 2015 damaged around 2.6 million ha of forest, releasing a billion tonnes of carbon dioxide equivalent (16). This led to new and enhanced regulations on peatland, a new Peatland Restoration Agency (BRG) and increased policy priority for fire prevention, and the expansion of the Moratorium to peatland forest to the whole country (17). All three types of concession were similarly affected. Thus, the treatment effect of the Moratorium on peatland forest, based on the 2011 boundaries, is estimated for all periods between 2011 and 2017 while the effect on dryland forest is also estimated for the period 2011-18. In September 2018, a new three-year Moratorium targeting new palm oil plantations across the whole country began but this is not considered in our analysis due to coming into effect at the end of our study period.

How the Indonesia-Norway partnership estimated forest-based emissions reductions in 2017. In 2017, Indonesia reportedly reduced emissions from deforestation and forest degradation by 11.2 MtCO_2-eq, the first reduction since the start of the Moratorium (1). Norway subsequently announced that it would pay Indonesia US$ 56.2 million. In this sub-section, we summarise how these emissions reductions were estimated, drawing on a report produced by Indonesia's Ministry of Environment and Forestry (18). This report was submitted to Norway's government and provides the evidence base for estimating payments from Norway's government to Indonesia's government in the event of reductions of GHG emissions from deforestation and forest degradation, for the period 2017-2020.

The reference emission level adopted as a baseline for estimating changes in emissions (in tons of carbon dioxide equivalent per year) and hence, Indonesia's REDD+ performance under the framework of Norway-Indonesia Partnership, is the Result Based Payment (RBP) baseline. To be updated periodically every five years, the RBP baseline has a different reference period (2006-2016) from Indonesia's Forest Reference Emission Level (FREL) that was submitted to the UNFCCC in 2016 (1990-2012).

The RBP baseline covers all "natural forests" in Indonesia, defined as 'a land area of more than 6.25 hectares with trees higher than 5 meters at maturity and a canopy cover of more than 30 percent'. These forests are classified according to land cover maps produced by the National Forest Monitoring System (NFMS) and used by the Ministry of Environment and Forestry. There are six natural forest classes that are used to estimate the RBP: primary dryland forest, secondary dryland forest, primary swamp forest, secondary swamp forest, primary mangrove forest, and secondary mangrove forest. Map data for the years 2006, 2009, 2011, 2012, 2013, 2014, 2015 and 2016 are used to analyse historical land cover changes and calculate changes in emissions. Forest data were sourced from Landsat 5 Thematic Mapper (TM) and Landsat 7 Enhanced Thematic Mapper Plus (ETM+).

Not all natural forests in Indonesia are included in the estimation of the RSB (or the FREL) and although it is clear that land-use designation is a critical factor determining which forest areas are included or excluded, there is little information on precisely how designation has affected these estimates. In 2016, a forest estate of 95.2 million ha was designated according to "permanent forest" (87.7 million ha) and "forest area designated for conversion to other land uses" (7.5 million ha; APL - see above) (19). Permanent forest includes "protection forest" (24.0 million ha), "conservation/fully protected forest and

recreation forests" (17.4 million ha), and "production forest" (46.2 million ha). The last, the biggest forest category by area, contains forests designated for the exploitation of timber and other forest products. According to Indonesia's FREL submission to the UNFCCC in 2016, up 20 million ha of permanent forest (specifically that which, by law, is slated for conversion) and APL forest is excluded from the estimation of Indonesia's FREL (20). Further inference is not possible due to the focus of estimates of the RSB and the FREL on the levels of deforestation rather than on levels of forest cover.

For both the RSB and FREL, annual forest cover change was estimated by overlaying land cover maps of two subsequent periods. Deforestation is defined as the change of natural forests from a forest class to a non-forest class at a given location in the period 2006-2016, while degradation occurs when a change from a primary to a secondary forest class is identified. Calculation of the RBP baseline used emission factors for each forest class and are identical to those used in Indonesia's first FREL. These emission factors were estimated using data primarily collected by the National Forest Inventory (NFI). Average annual emissions from deforestation and forest degradation in the period 2006-2016 were estimated to be 236.9 $MtCO_2$-eq/yr and 41.6 $MtCO_2$-eq/yr, respectively. Actual emissions in 2017 were estimated to be 228.3 $MtCO_2$-eq/yr and 42.7 $MtCO_2$-eq/yr from deforestation and forest degradation, respectively.

Against the 2006-2016 RBP baseline, Indonesia reduced emissions in 2017 by 7.4 $MtCO_2$-eq/yr: 8.6 $MtCO_2$-eq/yr from avoided deforestation (3.6% below the baseline) net of an increase in emissions from forest degradation, 1.2 $MtCO_2$-eq/yr (2.9% above the baseline). Uncertainties related to the forest data and emissions factors used were calculated following IPCC guidelines (2006). To account for statistical uncertainty, the risk of carbon reversals and leakage, a "set aside factor" of 35% was applied to the estimates of emissions reductions, giving a net reduction of 4.8 $MtCO_2$-eq/yr.

Also estimated were emissions from changes in peatland (swamp) forest, both from peat decomposition and peat fires. The former was defined as the changing process of peat form as a result of a decline in water levels caused by deforestation and forest degradation, and land utilization. When peat is drained and exposed, it oxidises thus generating carbon dioxide. Emission factors for peat decomposition also followed IPCC guidelines, based on the assumption that all utilized areas are drained. The calculation of emissions from peat decomposition was otherwise identical to the method used for calculating emissions from deforestation and forest degradation but with the inclusion of 'inherited' emissions from peat decomposition. In 2017, 256.7 $MtCO_2$-eq/yr was emitted from the decomposition of peat, which when compared to the RBP baseline of 260.6 $MtCO_2$-eq/yr, generated an emissions reduction of 3.9 $MtCO_2$-eq/yr.

Emissions from peat fires were estimated using data on burn scar areas. Fire incidence in peatland was 13,555 hectares in 2017. Combined with estimates of the mass of fuel available for combustion, to generate emission factors for burned peat, this amounted to 12.5 $MtCO_2$-eq/yr. Against an annual emission baseline of 249 $MtCO_2$-eq/yr between 2006 and 2016, there was an estimated emission reduction of 236.4 $MtCO_2$-eq/yr, in 2017. In contrast to estimates of emissions from deforestation and forest degradation, these estimates of peat fire emissions were subject to considerably more uncertainty, which was not possible to quantify.

Perhaps due to this uncertainty, Indonesia's government initially excluded emissions from peat fires in its calculations. However, Norway's government factored in peat fires, coming up with a total emissions reduction estimate of 11.2 $MtCO_2$-eq/yr for 2017 (1). Multiplied by a carbon price of US$5 per ton, set by Norway, this generated an estimated payment of $US 56.2 million to be paid by Norway to Indonesia for emissions reductions from deforestation and forest degradation in 2017.

2. Main and Additional Results

ATT results: year-on-year. The ATT estimates in the following tables are our main results, which are used to derive estimates of avoided forest cover loss and carbon emissions reductions.

Table S1. DD dryland forest ATT

Endpoint	ATT	AI SE	t-stat	p-value	Treated	Matched	Dropped (treated)
**2012**	0.137***	0.0380	3.641	0.000	160012	152118	7894
**2013**	0.237***	0.0510	4.633	0.000	160012	152118	7894
**2014**	0.318***	0.0670	4.762	0.000	160012	152118	7894
**2015**	0.472***	0.0780	6.037	0.000	160012	152118	7894
**2016**	0.718***	0.0940	7.674	0.000	160012	152118	7894
**2017**	0.813***	0.0990	8.186	0.000	160012	152118	7894
**2018**	0.938***	0.1050	8.892	0.000	160012	152118	7894

Notes: Abadie-Imbens (21) standard errors.

Maximum caliper width: 0.0001. 1-to-1 matching with replacement, keeping ties.

$^{*}\ p < 0.1$, $^{**}\ p < 0.05$, $^{***}\ p < 0.01$.

Table S2. DDD dryland forest ATT

Endpoint	ATT	AI SE	t-stat	p-value	Treated	Matched	Dropped (treated)
**2012**	0.108***	0.0390	2.761	0.006	160012	152118	7894
**2013**	0.178***	0.0570	3.146	0.002	160012	152118	7894
**2014**	0.229***	0.0760	2.998	0.003	160012	152118	7894
**2015**	0.354***	0.0940	3.780	0.000	160012	152118	7894
**2016**	0.571***	0.1140	4.991	0.000	160012	152118	7894
**2017**	0.637***	0.1280	4.984	0.000	160012	152118	7894
**2018**	0.732***	0.1420	5.150	0.000	160012	152118	7894

Notes: Abadie-Imbens (21) standard errors.

Maximum caliper width: 0.0001. 1-to-1 matching with replacement, keeping ties.

* $p < 0.1$, ** $p < 0.05$, *** $p < 0.01$.

Table S3. DD peatland forest ATT

Endpoint	ATT	AI SE	t-stat	p-value	Treated	Matched	Dropped (treated)
**2012**	-0.016	0.0390	-0.414	0.679	95154	91024	4130
**2013**	-0.052	0.0530	-0.979	0.328	95154	91024	4130
**2014**	-0.153**	0.0710	-2.163	0.031	95154	91024	4130
**2015**	-0.073	0.0820	-0.883	0.377	95154	91024	4130
**2016**	-0.032	0.0960	-0.333	0.739	95154	91024	4130
**2017**	-0.047	0.1010	-0.461	0.645	95154	91024	4130

Notes: Abadie-Imbens (21) standard errors.
Maximum caliper width: 0.0001. 1-to-1 matching with replacement, keeping ties.
$^{*}\ p < 0.1$, $^{**}\ p < 0.05$, $^{***}\ p < 0.01$.

Table S4. DDD peatland forest ATT

Endpoint	ATT	AI SE	t-stat	p-value	Treated	Matched	Dropped (treated)
**2012**	-0.011	0.0410	-0.260	0.795	95154	91024	4130
**2013**	-0.041	0.0600	-0.680	0.496	95154	91024	4130
**2014**	-0.136*	0.0830	-1.651	0.099	95154	91024	4130
**2015**	-0.05	0.1010	-0.497	0.619	95154	91024	4130
**2016**	-0.004	0.1220	-0.033	0.974	95154	91024	4130
**2017**	-0.013	0.1370	-0.096	0.923	95154	91024	4130

Notes: Abadie-Imbens (21) standard errors.
Maximum caliper width: 0.0001. 1-to-1 matching with replacement, keeping ties.
$^{*}\ p < 0.1$, $^{**}\ p < 0.05$, $^{***}\ p < 0.01$.

Estimated avoided forest cover loss and carbon emissions reductions. The estimates of avoided forest cover loss and carbon emissions reductions in the following tables can be seen graphically in Fig. 2.

Table S5. DD dryland forest avoided forest cover loss and carbon emissions reductions

Endpoint	Emissions reduction (MtCO_2-eq)	Avoided forest loss ('000 ha)
2012	12.723	21.967
2013	21.993	37.972
2014	29.441	50.830
2015	43.789	75.603
2016	66.550	114.901
2017	75.393	130.168
2018	86.931	150.089

Table S6. DDD dryland forest avoided forest cover loss and carbon emissions reductions

Endpoint	Emissions reduction (MtCO_2-eq)	Avoided forest loss ('000 ha)
2012	9.990	17.248
2013	16.526	28.533
2014	21.240	36.672
2015	32.855	56.725
2016	52.883	91.303
2017	58.992	101.851
2018	67.797	117.053

Table S7. DD peatland forest avoided forest cover loss and carbon emissions reductions

Endpoint	Emissions reduction ($MtCO_2$-eq)	Avoided forest loss ('000 ha)
2012	-0.677	-1.541
2013	-2.165	-4.929
2014	-6.403	-14.578
2015	-3.041	-6.924
2016	-1.336	-3.042
2017	-1.954	-4.449

Table S8. DDD peatland forest avoided forest cover loss and carbon emissions reductions

Endpoint	Emissions reduction ($MtCO_2$-eq)	Avoided forest loss ('000 ha)
2012	-0.443	-1.009
2013	-1.698	-3.866
2014	-5.703	-12.983
2015	-2.107	-4.798
2016	-0.169	-0.384
2017	-0.553	-1.259

Robustness checks (2017 endpoint). The following tables show the results from testing the robustness of our main results.

Table S9. DD dryland forest trimmed sample results (2017 endpoint)

	ATT	AI SE	t-stat	p-value	Treated	Matched	Dropped (treated)
**Untrimmed**	0.813***	0.0990	8.186	0.000	160012	152118	7894
**30%**	0.659***	0.1090	6.055	0.000	144501	136472	8029
**60%**	0.79***	0.1210	6.558	0.000	127375	119284	8091

Notes: Abadie-Imbens (21) standard errors.
Maximum caliper width: 0.0001. 1-to-1 matching with replacement, keeping ties.
$^{*}\ p < 0.1$, $^{**}\ p < 0.05$, $^{***}\ p < 0.01$.

Table S10. DDD dryland forest trimmed sample results (2017 endpoint)

	ATT	AI SE	t-stat	p-value	Treated	Matched	Dropped (treated)
**Untrimmed**	0.637***	0.1280	4.984	0.000	160012	152118	7894
**30%**	0.443***	0.1390	3.193	0.001	144501	136472	8029
**60%**	0.593***	0.1500	3.958	0.000	127375	119284	8091

Notes: Abadie-Imbens (21) standard errors.
Maximum caliper width: 0.0001. 1-to-1 matching with replacement, keeping ties.
$^{*}\ p < 0.1$, $^{**}\ p < 0.05$, $^{***}\ p < 0.01$.

Table S11. DD peatland forest trimmed sample results (2017 endpoint)

	ATT	AI SE	t-stat	p-value	Treated	Matched	Dropped (treated)
**Untrimmed**	-0.047	0.1010	-0.461	0.645	95154	91024	4130
**30%**	-0.097	0.1110	-0.876	0.381	86897	82832	4065
**60%**	-0.186	0.1220	-1.532	0.126	77777	73554	4223

Notes: Abadie-Imbens (21) standard errors.
Maximum caliper width: 0.0001. 1-to-1 matching with replacement, keeping ties.
$^{*}\ p < 0.1$, $^{**}\ p < 0.05$, $^{***}\ p < 0.01$.

Table S12. DDD peatland forest trimmed sample results (2017 endpoint)

	ATT	AI SE	t-stat	p-value	Treated	Matched	Dropped (treated)
**Untrimmed**	-0.013	0.1370	-0.096	0.923	95154	91024	4130
**30%**	0.025	0.1480	0.169	0.866	86897	82832	4065
**60%**	-0.121	0.1580	-0.768	0.442	77777	73554	4223

Notes: Abadie-Imbens (21) standard errors.
Maximum caliper width: 0.0001. 1-to-1 matching with replacement, keeping ties.
$^{*}\ p < 0.1$, $^{**}\ p < 0.05$, $^{***}\ p < 0.01$.

Table S13. Coarsened Exact Matching dryland forest results (2017 endpoint)

	SATT	S.E.	t-stat	p-value	Treated	Matched	Dropped (treated)
**Model 1**	0.4868***	0.06	8.12	0.000	160012	125458	34554
**Model 2**	0.4786***	0.06	7.56	0.000	160012	141334	18678
**Model 3**	0.4664***	0.06	7.38	0.000	160012	141362	18650
**Model 4**	0.5221***	0.06	8.27	0.000	160012	141362	18650

Notes: $^{*}p < 0.05, ^{**}p < 0.01, ^{***}p < 0.001.$

Model 1: 2005-2010 forest cover, slope, elevation and distance from roads included in the matching algorithm.
Distance from roads uses manually defined bins in lieu of the automated ones.
Distance from palm oil, timber and logging concessions are included in the linear outcome model.

Model 2: 2005-2010 forest cover, slope, elevation, 2005 and 2010 population included in the matching algorithm.
Distance from roads and carbon stocks are included in the linear outcome model.

Model 3: 2005-2010 forest cover, slope, elevation and distance from cities included in the matching algorithm.
Distance from palm oil, timber and logging concessions, distance from roads, carbon stocks and
2000, 2005 and 2010 population are included in the linear outcome model.

Model 4: 2005-2010 forest cover, slope, and elevation included in the matching algorithm.
Distance from roads, carbon stocks, and 2000, 2005 and 2010 population
are included in the linear outcome model.

Table S14. Coarsened Exact Matching peatland forest results (2017 endpoint)

	SATT	S.E.	t-stat	p-value	Treated	Matched	Dropped (treated)
**Model 1**	-0.012	0.08	-0.15	0.884	95154	71378	23776
**Model 2**	0.2843***	0.08	3.62	0.000	95154	78322	16832
**Model 3**	-0.2327*	0.09	-2.50	0.012	95154	56523	38631
**Model 4**	0.1185	0.08	1.50	0.135	95154	80365	14789

Notes: $^{*}p < 0.05, ^{**}p < 0.01, ^{***}p < 0.001.$

Model 1: 2005-2010 forest cover, slope, elevation, 2005 and 2010 population. Moreover
Distance from roads, AGC and distance from palm oil use manually defined bins in lieu of the automated ones.

Model 2: 2005-2010 forest cover, slope, elevation, 2005 and 2010 population. Moreover
Distance from roads and AGC use manually defined bins in lieu of the automated ones.

Model 3: 2005-2010 forest cover, slope, elevation, 2005 and 2010 population. Moreover
Distance from roads and AGC use manually defined bins in lieu of the automated ones.
Distance from palm oil, timber and logging use manually defined bins in lieu of the automated ones.

Model 4: 2005-2010 forest cover, slope, elevation and distance from roads included in the matching algorithm.
Distance from palm oil, timber and logging concessions are included in the linear outcome model.
Distance from roads uses manually defined bins in lieu of the automated ones.

Table S15. DDD dryland forest ATT: caliper = 0.001

Endpoint	ATT	AI SE	t-stat	p-value	Treated	Matched	Dropped (treated)
**2012**	0.115**	0.0450	2.568	0.010	160012	159975	37
**2013**	0.176***	0.0650	2.724	0.006	160012	159975	37
**2014**	0.230***	0.0870	2.652	0.008	160012	159975	37
**2015**	0.373***	0.1070	3.492	0.000	160012	159975	37
**2016**	0.583***	0.1300	4.489	0.000	160012	159975	37
**2017**	0.646***	0.1450	4.454	0.000	160012	159975	37
**2018**	0.749***	0.1610	4.649	0.000	160012	159975	37

Notes: Abadie-Imbens (21) standard errors.

Maximum caliper width: 0.001. 1-to-1 matching with replacement, keeping ties.

* $p < 0.1$, ** $p < 0.05$, *** $p < .01$.

Table S16. DDD peatland forest ATT: caliper = 0.001

Endpoint	ATT	AI SE	t-stat	p-value	Treated	Matched	Dropped (treated)
**2012**	-0.008	0.0430	-0.194	0.846	95154	95004	150
**2013**	-0.035	0.0640	-0.550	0.582	95154	95004	150
**2014**	-0.121	0.0880	-1.374	0.170	95154	95004	150
**2015**	-0.047	0.1080	-0.440	0.660	95154	95004	150
**2016**	-0.013	0.1300	-0.100	0.920	95154	95004	150
**2017**	-0.021	0.1460	-0.147	0.883	95154	95004	150

Notes: Abadie-Imbens (21) standard errors.

Maximum caliper width: 0.001. 1-to-1 matching with replacement, keeping ties.

* $p < 0.1$, ** $p < 0.05$, *** $p < 0.01$.

Table S17. DDD dryland: robustness (2017 endpoint)

	ATT	AI SE	t-stat	p-value	Treated	Matched	Dropped (treated)
**1:2 Matching**	0.553***	0.1020	5.446	0.000	160012	138635	21377
**1:3 Matching**	0.54***	0.0850	6.346	0.000	160012	123432	36580
**1:5 Matching**	0.555***	0.0640	8.663	0.000	160012	95821	64191
**Above 1000 m**	0.223	0.1870	1.192	0.233	67693	49782	17911
**All elevation**	0.611***	0.1210	5.060	0.000	227705	224112	3593

Notes: Abadie-Imbens (21) standard errors.
Maximum caliper width: 0.0001. 1-to-1 matching with replacement, keeping ties.
* $p < 0.1$, ** $p < 0.05$, *** $p < 0.01$.

Table S18. DDD peatland: robustness (2017 endpoint)

	ATT	AI SE	t-stat	p-value	Treated	Matched	Dropped (treated)
**1:2 Matching**	-0.022	0.1160	-0.191	0.849	95154	82953	12201
**1:3 Matching**	-0.052	0.1010	-0.509	0.611	95154	71764	23390
**1:5 Matching**	-0.176**	0.0800	-2.200	0.028	95154	49637	45517
**Above 1000 m**	1.696***	0.1690	10.044	0.000	1937	108	1829
**All elevation**	-0.165	0.1350	-1.216	0.224	97091	93210	3881

Notes: Abadie-Imbens (21) standard errors.
Maximum caliper width: 0.0001. 1-to-1 matching with replacement, keeping ties.
* $p < 0.1$, ** $p < 0.05$, *** $p < 0.01$.

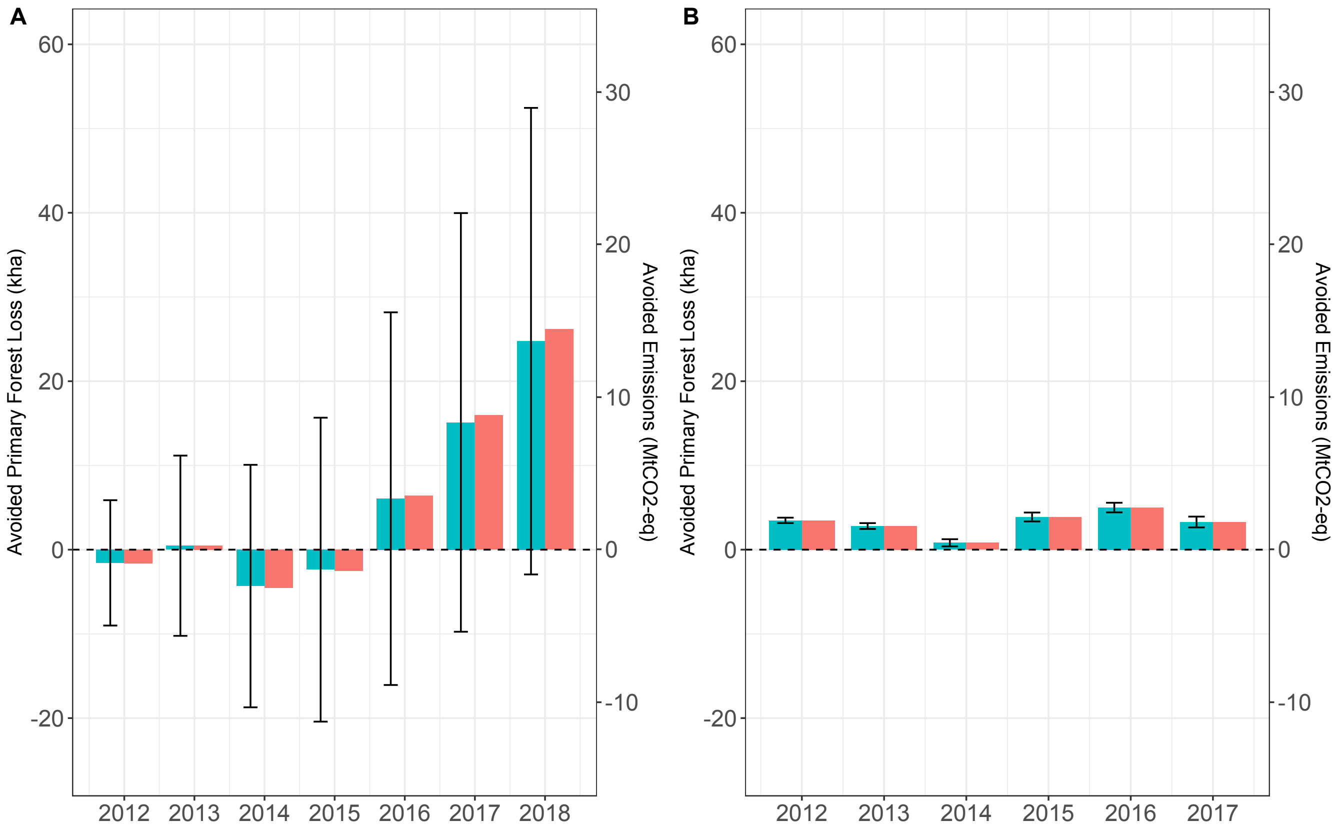

Fig. S3. Cumulative avoided forest loss ('000 ha) and avoided carbon dioxide emissions (MtCO_2-eq): Dryland forest > 1,000m DDD, 2012-2018 (A); Peatland > 1,000m DDD, 2012-2017 (B). The blue columns and left-hand y-axis in each panel shows the quantity of avoided forest loss while the red columns and right-hand y-axis shows the quantity of carbon emissions avoided. All quantities are aggregated up to the level of the whole Moratorium. Error bars denote the 95% CI.

“Intact primary” forest (22, 23) results. The following tables show the results from using a tighter definition of "forest" determined by extent of forest intactness and contiguity (22, 23).

Table S19. DD dryland “intact primary” forest (22, 23) ATT

Endpoint	ATT	AI SE	t-stat	p-value	Treated	Matched	Dropped (treated)
**2012**	-0.018	0.0250	-0.743	0.457	160012	152252	7760
**2013**	0.043	0.0360	1.194	0.232	160012	152252	7760
**2014**	0.08*	0.0480	1.667	0.095	160012	152252	7760
**2015**	0.172***	0.0560	3.070	0.002	160012	152252	7760
**2016**	0.285***	0.0670	4.270	0.000	160012	152252	7760
**2017**	0.306***	0.0700	4.370	0.000	160012	152252	7760
**2018**	0.325***	0.0730	4.434	0.000	160012	152252	7760

Notes: Abadie-Imbens (21) standard errors.
Maximum caliper width: 0.0001. 1-to-1 matching with replacement, keeping ties.
$^{*}\ p < 0.1$, $^{**}\ p < 0.05$, $^{***}\ p < 0.01$.

Table S20. DDD dryland “intact primary” forest (22, 23) ATT

Endpoint	ATT	AI SE	t-stat	p-value	Treated	Matched	Dropped (treated)
**2012**	-0.039	0.0260	-1.505	0.132	160012	152252	7760
**2013**	0.003	0.0390	0.067	0.947	160012	152252	7760
**2014**	0.019	0.0540	0.359	0.719	160012	152252	7760
**2015**	0.091	0.0650	1.407	0.159	160012	152252	7760
**2016**	0.184**	0.0780	2.343	0.019	160012	152252	7760
**2017**	0.185**	0.0860	2.138	0.033	160012	152252	7760
**2018**	0.184*	0.0950	1.936	0.053	160012	152252	7760

Notes: Abadie-Imbens (21) standard errors.
Maximum caliper width: 0.0001. 1-to-1 matching with replacement, keeping ties.
$^{*}\ p < 0.1$, $^{**}\ p < 0.05$, $^{***}\ p < 0.01$.

Table S21. DD peatland “intact primary” forest (22, 23) ATT

Endpoint	ATT	AI SE	t-stat	p-value	Treated	Matched	Dropped (treated)
**2012**	0.007	0.0280	0.265	0.791	95154	91302	3852
**2013**	-0.038	0.0390	-0.970	0.332	95154	91302	3852
**2014**	-0.132**	0.0530	-2.508	0.012	95154	91302	3852
**2015**	-0.104*	0.0610	-1.703	0.089	95154	91302	3852
**2016**	-0.104	0.0710	-1.459	0.145	95154	91302	3852
**2017**	-0.094	0.0740	-1.274	0.203	95154	91302	3852

Notes: Abadie-Imbens (21) standard errors.
Maximum caliper width: 0.0001. 1-to-1 matching with replacement, keeping ties.
$^{*}\ p < 0.1$, $^{**}\ p < 0.05$, $^{***}\ p < 0.01$.

Table S22. DDD peatland "intact primary" forest (22, 23) ATT

Endpoint	ATT	AI SE	t-stat	p-value	Treated	Matched	Dropped (treated)
**2012**	0.002	0.0290	0.078	0.938	95154	91302	3852
**2013**	-0.048	0.0430	-1.116	0.264	95154	91302	3852
**2014**	-0.148**	0.0600	-2.474	0.013	95154	91302	3852
**2015**	-0.124*	0.0720	-1.714	0.087	95154	91302	3852
**2016**	-0.129	0.0870	-1.486	0.137	95154	91302	3852
**2017**	-0.125	0.0970	-1.297	0.195	95154	91302	3852

Notes: Abadie-Imbens (21) standard errors.

Maximum caliper width: 0.0001. 1-to-1 matching with replacement, keeping ties.

$^{*}\ p < 0.1$, $^{**}\ p < 0.05$, $^{***}\ p < 0.01$.

Table S23. DD dryland "intact primary" forest (22, 23) avoided forest cover loss and carbon emissions reductions

Endpoint	Emissions reduction ($MtCO_2$-eq)	Avoided forest loss ('000 ha)
2012	-1.711	-2.955
2013	3.987	6.883
2014	7.397	12.771
2015	15.956	27.549
2016	26.398	45.577
2017	28.347	48.942
2018	30.120	52.003

Table S24. DDD dryland "intact primary" forest (22, 23) avoided forest cover loss and carbon emissions reductions

Endpoint	Emissions reduction ($MtCO_2$-eq)	Avoided forest loss ('000 ha)
2012	-3.58	-6.186
2013	0.243	0.42
2014	1.782	3.076
2015	8.47	14.623
2016	17.04	29.42
2017	17.11	29.554
2018	17.01	29.383

Table S25. DD peatland "intact primary" forest (22, 23) avoided forest cover loss and carbon emissions reductions

Endpoint	Emissions reduction ($MtCO_2$-eq)	Avoided forest loss ('000 ha)
2012	0.310	0.706
2013	-1.569	-3.572
2014	-5.537	-12.607
2015	-4.328	-9.853
2016	-4.326	-9.848
2017	-3.942	-8.975

Table S26. DDD peatland "intact primary" forest (22, 23) avoided forest cover loss and carbon emissions reductions

Endpoint	Emissions reduction ($MtCO_2$-eq)	Avoided forest loss ('000 ha)
2012	0.095	0.216
2013	-2.00	-4.553
2014	-6.183	-14.078
2015	-5.189	-11.814
2016	-5.402	-12.299
2017	-5.234	-11.917

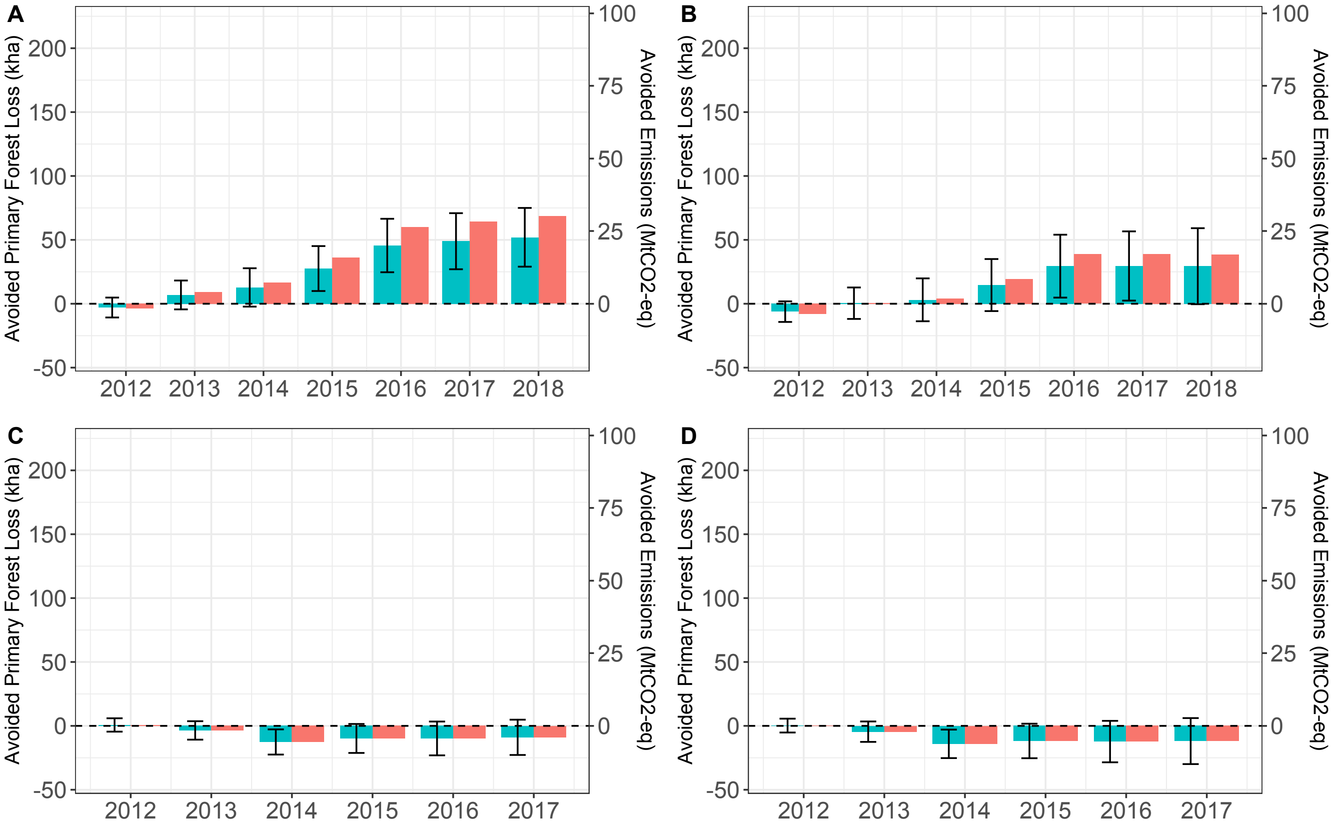

Fig. S4. Cumulative avoided "intact primary" forest (22, 23) loss ('000 ha) and avoided carbon dioxide emissions ($MtCO_2$-eq): dryland forest DD, 2012-2018 (A); dryland forest DDD, 2012-2018 (B); peatland forest DD, 2012-2017 (C); peatland DDD, 2012-2017 (D). The blue columns and left-hand y-axis in each panel shows the quantity of avoided forest loss while the red columns and right-hand y-axis shows the quantity of carbon emissions avoided. All quantities are aggregated up to the level of the whole Moratorium. Error bars denote the 95% CI.

Spatially heterogeneous effects of the Moratorium, 2011-17. Fig. S5 shows the heterogeneous effects of the Moratorium on dryland forest cover using district-level ATT generated from the aggregate ATT. Relatively large positive effects (shaded dark green) are revealed in the mountainous regions of Sumatra, districts in East and South Kalimantan, as well as parts of Java and Papua. Some of these districts are also the location of high shares of the total area of Indonesia's land designated as concessions pre-2011 (Fig. S1) but this is not a consistent pattern across the country. For example, there is little or no effect in the lowlands of Sumatra, nor in parts of Central Kalimantan, where many palm oil and timber concessions are located.

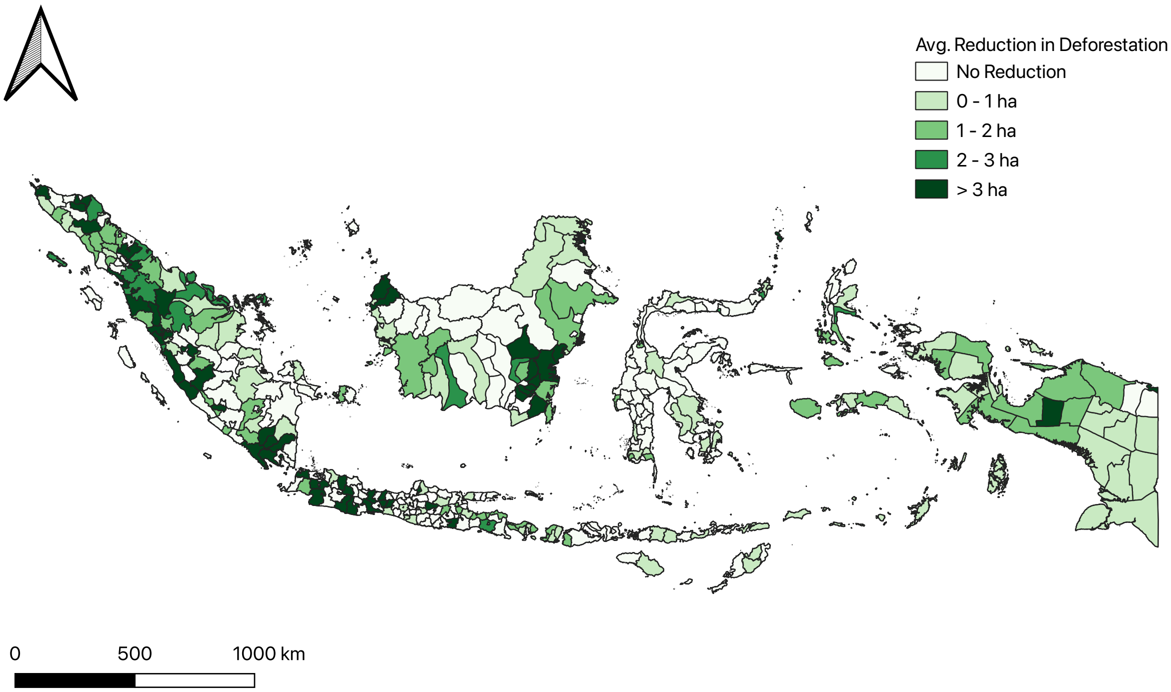

Fig. S5. Spatially heterogeneous effects of the Moratorium on dryland and peatland forests, 2011-17

3. Summary Statistics

Table S27. Dryland forest non-concession grid cells, Moratorium

Statistic	Mean	S.D.	Min	Max	N_{obs}
**AGC**	157.819	38.253	0.500	239.517	160012
**Distance cities**	1588.080	1340.752	2.000	6682.944	160012
**Distance logging**	87.257	172.887	0.00003	903.985	160012
**Distance palm oil**	102.348	141.246	0.00004	679.341	160012
**Distance timber**	123.843	143.885	0.00004	584.680	160012
**Distance roads**	35.148	32.350	0.056	220.476	160012
**Elevation**	470.037	271.519	−0.375	999.991	160012
**Peat depth**	0.000	0.000	0	0	160012
**Slope**	13.141	6.673	0.000	41.013	160012
**Population 2005**	17.298	135.354	0.000	10718.960	160012
**Population 2010**	14.925	126.867	0.000	10194.360	160012
**Forest cover 2005**	109.083	40.862	0.000	142.402	160012
**Forest cover 2011**	103.784	44.237	0.000	142.050	160012
**Forest cover 2016**	98.062	47.467	0.000	142.050	160012
**Forest cover 2017**	97.259	47.890	0.000	141.850	160012
**Forest cover 2018**	96.497	48.281	0.000	141.850	160012

Table S28. Dryland forest non-concession grid cells, non-Moratorium

Statistic	Mean	S.D.	Min	Max	N_{obs}
**AGC**	92.168	52.597	0.500	239.803	407622
**Distance cities**	579.065	809.565	0.000	6155.591	407622
**Distance logging**	230.052	253.851	0.00000	911.465	407622
**Distance palm oil**	167.752	197.904	0.00002	685.338	407622
**Distance timber**	128.035	168.030	0.00003	584.152	407622
**Distance roads**	12.341	21.137	0.000	283.699	407622
**Elevation**	207.618	229.919	−3.000	999.982	407622
**Peat depth**	0.000	0.000	0	0	407622
**Slope**	6.146	5.456	0.000	39.215	407622
**Population 2005**	328.561	1595.480	0	107782	407622
**Population 2010**	328.886	1474.688	0	87358	407622
**Forest cover 2005**	95.494	44.674	0.000	143.305	407622
**Forest cover 2011**	87.948	46.892	0.000	143.215	407622
**Forest cover 2016**	79.604	49.271	0.000	143.215	407622
**Forest cover 2017**	78.393	49.590	0.000	143.215	407622
**Forest cover 2018**	77.283	49.902	0.000	143.215	407622

Table S29. Dryland forest concession grid cells, Moratorium

Statistic	Mean	S.D.	Min	Max	N_{obs}
**AGC**	156.486	37.495	6.432	225.012	8577
**Distance cities**	1534.415	1090.362	17	6389	8577
**Distance logging**	19.875	63.949	0.0001	754.726	8577
**Distance palm oil**	46.345	90.734	0.0005	634.679	8577
**Distance timber**	56.398	98.799	0.00002	535.576	8577
**Distance roads**	31.006	26.855	0.193	157.039	8577
**Elevation**	351.910	251.125	0.873	999.917	8577
**Peat depth**	0.000	0.000	0	0	8577
**Slope**	11.088	6.683	0.078	35.263	8577
**Population 2005**	7.406	50.199	0.000	2385.000	8577
**Population 2010**	6.910	50.805	0.000	2481.000	8577
**Forest cover 2005**	105.033	42.964	0.000	141.074	8577
**Forest cover 2011**	98.834	46.061	0.000	141.074	8577
**Forest cover 2016**	92.489	49.188	0.000	141.074	8577
**Forest cover 2017**	91.435	49.675	0.000	141.074	8577
**Forest cover 2018**	90.567	50.063	0.000	141.074	8577

Table S30. Dryland forest concession grid cells, non-Moratorium

Statistic	Mean	S.D.	Min	Max	N_{obs}
**AGC**	139.703	46.721	0.500	242.199	254107
**Distance cities**	1422.768	1131.570	0.778	6257.167	254107
**Distance logging**	29.753	66.249	0.00002	757.565	254107
**Distance palm oil**	45.676	87.319	0.0001	638.973	254107
**Distance timber**	53.801	108.858	0.0001	568.691	254107
**Distance roads**	30.790	29.945	0.089	184.405	254107
**Elevation**	193.861	202.703	0.507	999.970	254107
**Peat depth**	0.000	0.000	0	0	254107
**Slope**	6.500	5.413	0.000	40.154	254107
**Population 2005**	15.568	131.062	0.000	29963.160	254107
**Population 2010**	15.141	104.916	0.000	12425.760	254107
**Forest cover 2005**	104.498	42.969	0.000	143.123	254107
**Forest cover 2011**	97.803	46.314	0.000	143.123	254107
**Forest cover 2016**	90.610	49.492	0.000	143.123	254107
**Forest cover 2017**	89.656	49.873	0.000	143.123	254107
**Forest cover 2018**	88.787	50.232	0.000	143.123	254107

Table S31. Peatland forest non-concession grid cells, Moratorium

Statistic	Mean	S.D.	Min	Max	N_{obs}
**AGC**	119.680	52.600	0.500	248.805	95154
**Distance cities**	1738.694	1250.135	1.000	6541.667	95154
**Distance logging**	49.646	63.999	0.0001	862.399	95154
**Distance palm oil**	59.646	60.120	0.0001	672.619	95154
**Distance timber**	134.295	143.310	0.0001	585.525	95154
**Distance roads**	54.333	43.582	0.022	231.603	95154
**Elevation**	87.082	162.877	−1.715	999.828	95154
**Peat depth**	5.018	3.314	1	10	95154
**Slope**	3.292	5.535	0.00000	44.659	95154
**Population 2005**	21.879	190.073	0.000	14271.050	95154
**Population 2010**	20.223	199.281	0.000	18457.200	95154
**Forest cover 2005**	111.444	39.854	0.000	143.530	95154
**Forest cover 2011**	106.188	43.791	0.000	143.530	95154
**Forest cover 2016**	100.822	47.414	0.000	143.530	95154
**Forest cover 2017**	100.116	47.855	0.000	143.530	95154
**Forest cover 2018**	99.418	48.270	0.000	143.530	95154

Table S32. Peatland forest non-concession grid cells, non-Moratorium

Statistic	Mean	S.D.	Min	Max	N_{obs}
**AGC**	68.444	48.624	0.500	230.178	191668
**Distance cities**	783.989	987.146	0	6110	191668
**Distance logging**	125.511	170.883	0.00001	909.426	191668
**Distance palm oil**	77.761	130.750	0.0001	669.294	191668
**Distance timber**	89.125	138.527	0.00002	584.481	191668
**Distance roads**	24.147	36.776	−0.096	240.210	191668
**Elevation**	48.089	109.444	−6.848	999.334	191668
**Peat depth**	4.234	2.959	1	10	191668
**Slope**	1.985	3.056	0.000	36.552	191668
**Population 2005**	429.286	2444.776	0.000	106258.800	191668
**Population 2010**	435.098	2318.126	0.000	85667.030	191668
**Forest cover 2005**	98.455	43.815	0.000	142.203	191668
**Forest cover 2011**	89.987	46.968	0.000	141.715	191668
**Forest cover 2016**	81.242	49.863	0.000	141.715	191668
**Forest cover 2017**	80.026	50.222	0.000	141.715	191668
**Forest cover 2018**	78.896	50.573	0.000	141.715	191668

Table S33. Peatland forest concession grid cells, Moratorium

Statistic	Mean	S.D.	Min	Max	N_{obs}
**AGC**	108.714	52.756	0.897	226.424	6930
**Distance ities**	1219.530	984.166	13	5424	6930
**Distance logging**	46.441	53.984	0.0001	271.455	6930
**Distance palm oil**	17.699	36.409	0.0001	409.311	6930
**Distance timber**	63.798	91.195	0.00002	557.789	6930
**Distance roads**	34.717	35.464	0.146	189.795	6930
**Elevation**	54.809	100.569	1.411	890.440	6930
**Peat depth**	5.615	3.460	1.000	10.000	6930
**Slope**	2.586	4.481	0.016	32.867	6930
**Population 2005**	15.282	56.990	0.000	1632.000	6930
**Population 2010**	11.285	46.319	0.000	1945.800	6930
**Forest cover 2005**	100.037	45.993	0.000	141.297	6930
**Forest cover 2011**	93.515	48.552	0.000	141.297	6930
**Forest cover 2016**	86.579	51.578	0.000	141.297	6930
**Forest cover 2017**	85.614	51.923	0.000	141.297	6930
**Forest cover 2018**	84.581	52.327	0.000	141.297	6930

Table S34. Peatland forest concession grid cells, non-Moratorium

Statistic	Mean	S.D.	Min	Max	N_{obs}
**AGC**	112.349	55.941	0.595	240.324	113954
**Distance cities**	1519.586	1199.065	3.167	6291.000	113954
**Distance logging**	39.990	56.940	0.00002	732.571	113954
**Distance palm oil**	32.212	49.361	0.0001	636.106	113954
**Distance timber**	65.691	119.646	0.0003	571.354	113954
**Distance roads**	38.486	34.998	0.034	189.846	113954
**Elevation**	62.648	96.492	0.267	998.759	113954
**Peat depth**	4.837	3.439	1.000	10.000	113954
**Slope**	2.516	3.568	0.000	34.959	113954
**Population 2005**	19.275	99.717	0.000	9538.440	113954
**Population 2010**	17.854	87.030	0.000	9514.040	113954
**Forest cover 2005**	104.618	42.932	0.000	141.937	113954
**Forest cover 2011**	97.172	46.966	0.000	141.686	113954
**Forest cover 2016**	89.343	50.675	0.000	141.322	113954
**Forest cover 2017**	88.366	51.077	0.000	141.322	113954
**Forest cover 2018**	87.461	51.441	0.000	141.322	113954

4. Methods

Summary of empirical approach. The impacts of the 2011 Moratorium on forest cover and carbon emissions are estimated for the period starting in 2011 and ending variously in years 2012-2018. Up until 2017, the estimates are undertaken separately for dryland and peatland forest but for the period 2011-2018 the estimates are for dryland forest only due to the additional restrictions on peatland implemented in 2017 (17). Pre- and post-Moratorium panel data on forest cover spanning the period 2000-2018 are used. Identification of the causal effect stems from a difference-in-differences (DD) research design, which allows for a comparison of the treated (Moratorium) grid cells with untreated, control (non-Moratorium) grid cells, while controlling for pre-existing differences and a secular counterfactual trend in forest cover. A matching approach is taken so that counterfactual cells have the same or similar characteristics as the treated cells. A key identification assumption is that of parallel trends in unobservable cell characteristics between Moratorium and non-Moratorium cells. Although impossible to test directly, we undertake several placebo tests in order to check this assumption. The main analysis uses pre-treatment data from 2004. The placebo tests require data from before the time horizon considered in the main analysis and so also use data from 2000 to 2004. DD can be implemented in a number of ways, and decisions have to be taken about the appropriate DD estimator to deploy. We select among a number of different parametric and non-parametric (matched) DD and triple difference (DDD) estimators through a process of empirical testing of typical DD identification assumptions, and a sensitivity analysis of matching estimators and standard parametric (linear) models. This process of model selection culminates with a preference for a matched triple differences estimator following (24, 25). After outlining the identification problem, the following sub-sections provide: an account of the estimators; our process of model selection via empirical testing of their identification assumptions, including placebo tests; and, the robustness checks applied to our main results, including sensitivity to matching procedures and a test of the Standard Unit Treatment Value Assumption (SUTVA), another identification assumption for DD.

The identification problem. The core empirical problem with evaluating the causal impact of the Moratorium is one of selection bias: the forest areas *treated* by the Moratorium differ in their observable and unobservable characteristics. Any imbalance in these characteristics implies that a direct comparison of forest cover in Moratorium and untreated non-Moratorium areas will capture pre-existing imbalances in, for example, their suitability for palm oil or timber production, level of forest cover or susceptibility to deforestation. These factors would then confound the estimate of the treatment effect. This generic programme evaluation problem, and the associated imbalances in underlying characteristics, is common in the analysis of area-based conservation policies (26–28). Protected areas, for instance, are often established in economically-marginal land with "few profitable alternative uses" (26, 29). Forest areas covered by the Moratorium are similarly distinctive from those not covered by the Moratorium.

Fig. 1 shows the average level of forest cover for grid cells outside concessions and cells within concessions, for both Moratorium and non-Moratorium areas. Areas under the Moratorium start with higher levels of forest cover in 2000, and this remains true throughout the period of observation and for both peatland and dryland forest, and for all concession types (palm oil, logging and timber). In all but one within-concession case, there are clear differences in forest levels between the treated and control cells. Fig. 1 also illustrates forest cover trends over our observation period. The general picture is one of steady decline, with forest cover declining by at least 10 percentage points over the 18-year observation period. These trends are steeper in concession cells than in non-concession cells irrespective of whether forest is part of the Moratorium or not. Tables S27-S34 present descriptive statistics illustrating additional differences between the Moratorium and non-Moratorium forest areas in terms of the key variables that determine the likelihood of forest areas becoming new concessions.

The essence of the DD design is depicted in Fig. 1. We have pre-and post-Moratorium data, and spatially well-defined treatment (Moratorium) and control (non-Moratorium) groups. A simple parametric DD would identify the impact of the Moratorium by estimating the difference in forest cover before and after the Moratorium was implemented in areas covered by the Moratorium, and takes from this the difference in forest cover in the non-Moratorium areas. Under certain assumptions DD approaches identify causal impacts, removing both the pre-existing differences between Moratorium and non-Moratorium areas, which would confound a simple comparison of treatment and control, and the trend in non-Moratorium areas under the assumption that this trend would have continued in the post-implementation period in Moratorium areas. Two key assumptions for the control area trend to act as a counterfactual for the treatment and DD estimates to be interpreted as causal are: (1) the unobservable trends in both Moratorium and non-Moratorium areas are the same on average; and, (2) the Stable Unit Treatment Value Assumption (SUTVA), that is, there are no spillovers ('leakage') from Moratorium areas to non-Moratorium areas which would confound the estimated treatment effect. In what follows we explain how we address these identification issues.

Two further issues are noteworthy for our empirical approach. First, the Moratorium mandates that district governments stop issuing new concession licenses in forest areas covered by the Moratorium. Yet, after 2011 licenses continued to be issued in forest areas that dropped out of the Moratorium determined by boundaries established in 2011. Our measure of impact is based on a treatment group with boundaries that were established in 2011, and we assume that any concession-driven deforestation observed within these boundaries after 2011 violated the Moratorium. Second, our empirical strategy must overcome the fact that after 2011 we do not observe where new oil palm, timber and logging concessions would have located in the absence of the Moratorium, in forest areas covered by the Moratorium. For this reason, the empirical strategy must control for characteristics of grid cells that determine the likelihood becoming a new concession, and ensure that these characteristics are the same or similar between Moratorium and non-Moratorium areas. The hope is that by *balancing* the observable characteristics, the confounding effect of unobservable characteristics will also be balanced or controlled for (30–32).

We take several steps to ensure that the grid cells compared in the Moratorium and non-Moratorium forest areas are similar in terms of their observable characteristics. First, we exclude cells which are part of the Indonesian protected area network, both within and outside the Moratorium, as conversion in these cells is already strictly prohibited. Second, we remove all cells outside of concessions with an elevation of 1,000 metres or more above sea level. The likelihood of these cells being a realistic proposition for a concession in either Moratorium or non-Moratorium areas is close to zero because above 1,000 metres land is unsuitable for palm oil cultivation (33) and for *Acacia mangium*, the main tree species employed for the production of wood pulp and paper (34). Robustness to this choice of sample is discussed below, in the final sub-section. Third, as noted earlier, we undertake separate analyses for dryland and peatland areas. These three steps can be thought of as a type of 'matching' on elevation and land types, whereby Moratorium and non-Moratorium grid cells are constrained to be similar in these dimensions. Lastly, we use a suite of matching approaches to ensure the analysis takes place on a sample whose Moratorium grid cells can be matched to non-Moratorium grid-cells on the basis of a set of more specific grid-level characteristics. The main analysis uses the sample arising from a one-to-one propensity score caliper matching procedure. Other matching procedures are used to check robustness to this particular procedure. In the propensity score estimates, the sample ensures common support in the propensity score (30). The matched dataset for the main analysis is used for parametric and non-parametric estimators. For the analysis of the impact of the Moratorium across forest types (peatland, dryland) and land uses (concession, non-concession), matching approaches are applied separately. For instance, the propensity score is estimated separately for each forest type, and the matched dataset upon which the impact estimated differs from one forest type to another.

Our process of model selection involves a sequence of placebo and falsification tests, which provide insights on the likelihood that the identification assumption of DD, parallel trends, holds. Finally, we test the SUTVA assumption. This process leads us to select a triple differences (DDD) estimator, which provides a correction for non-parallel trends. The estimators and the process of model selection are now explained in detail.

Estimators. We use a number of estimators to estimate the causal impact of the Moratorium. We estimate the Average Treatment on the Treated (ATT_{DD}) using a DD design, using both parametric and a non-parametric propensity score matching estimator.

The observed data on the outcome variable, Y_{it}, for individual grid-cell i at time t, are generated via a switching process governed by the occurrence of the Moratorium, which is indicated by the variable D_{it} :

$$Y_{it} = Y_{0it} + D_{it}\left(Y_{1it} - Y_{0it}\right)$$

where D_{it} is an indicator variable that interacts a variable for pre- and post- treatment periods, r_t, with an indicator for treatment status after the treatment period, $d_i : D_{it} = r_t d_i$ (35, p100-101). Y_{0it} and Y_{1it} are the potential outcomes for grid cell i in the treated state (1) and untreated state (0) at time t. Potential outcomes can be separated into their expected values, μ_{1it} and μ_{0it}, and a mean zero random component, ε_{kit}:

$$Y_{0it} = \mu_{0it} + \varepsilon_{0it}$$

$$Y_{1it} = \mu_{1it} + \varepsilon_{1it}$$

where $E\left[\varepsilon_{0it}\right] = E\left[\varepsilon_{1it}\right] = 0$. Suppose that the ε_{kit} are comprised of an individual fixed effect, λ_i, and a transitory component, u_{kit}: $\varepsilon_{kit} = \lambda_i + u_{kit}$. If the pre-treatment period is T_0 and the post-treatment period is T_1, then the difference-in-differences estimator uses the observed data from the switching equation to identify the Average Treatment on the Treated (ATT_{DD}) defined in terms of the potential outcomes as follows:

$$\begin{aligned}
DD &= E\left[\Delta_{T_1,T_0} Y_{it}|d_i = 1\right] - E\left[\Delta_{T_1,T_0} Y_{it}|d_i = 0\right] && [1]\\
&= \left\{E\left[\Delta_{T_1,T_0}\mu_{1it}|d_i = 1\right] - E\left[\Delta_{T_1,T_0}\mu_{0it}|d_i = 1\right]\right\}\\
&\quad + \left\{E[\Delta_{T_1,T_0} u_{1it}|d_i = 1] - E\left[\Delta_{T_1,T_0} u_{0it}|d_i = 0\right]\right\} && [2]\\
&= E\left[\Delta_{T_1,T_0} Y_{1it}|d_i = 1\right] - E\left[\Delta_{T_1,T_0} Y_{0it}|d_i = 1\right] && [3]\\
&= E\left[Y_{1iT_1} - Y_{0iT_1}|d_i = 1\right] = ATT_{DD} && [4]
\end{aligned}$$

where Eq. 1 uses the observed data, Eq. 2 reflects the potential outcomes, and Eq. 4 is simply a definition. Eq. 2 follows because the individual fixed effects, λ_i, drop out when first differences are taken. Eq. 3 follows under the assumption of parallel trends in unobservables, that is if:

$$E[\Delta_{T_1,T_0} u_{1it}|d_i = 1] - E\left[\Delta_{T_1,T_0} u_{0it}|d_i = 0\right] = 0$$

and Eq. 4 is the definition of the Average Treatment Effect and follows because by definition:

$$E\left[Y_{1iT_0} - Y_{0iT_0}|d_i = 1\right] = 0$$

meaning that potential outcomes pre-treatment are identical for the treated group.

Identification of ATT_{DD} using the DD estimator above does not rely on parametric assumptions, and is in principle a non-parametric estimator. With the assumption of a separable disturbance term: $\varepsilon_{kit} = \lambda_i + u_{kit}$, with individual heterogeneity, λ_i, fixed over time and between potential outcomes, the existence of panel data allows us to take differences within each individual grid cell, i, remove these fixed effects and effectively control for any endogeneity that might be associated with

individual heterogeneity of this type. A parametric fixed effects regression would use a linear model to control for individual heterogeneity in a similar way. We use a range of parametric and non-parametric matching DD estimators to estimate the treatment effect of the Moratorium. The parametric DD estimator is specified as:

$$Y_{it} = \alpha + \beta_1 D_{it} + \sum_{k=2}^{n} \beta_k X_{kit} + \lambda_i + \theta_t + \varepsilon_{it} \tag{5}$$

where Y_{it} is forest cover in (non-concession) grid cell i in year t, D_{it} is the time-varying Moratorium treatment indicator, X_{kit} are n time-varying control variables, which could include including climatic controls (minimum and maximum temperature, precipitation). Other controls, such as slope, elevation, and distance from cities and roads, are unnecessary for this particular estimation strategy because grid-cell fixed effects adjust for time-invariant controls. D_{it} is the "policy" variable taking the value 1 after 2010 for Moratorium areas, otherwise 0 and β_1 is the ATT. This basic model controls for time-invariant characteristics via the individual grid-level fixed effects, λ_i, and time fixed effects, θ_t, which capture shocks common to all grid cells such as weather shocks. The estimate of β_1 in Eq. 5 provides an estimate of ATT that reflects the average of the annual impacts over the post-treatment period (from period T_1 to T). Also interesting is to estimate the impact year-by-year, which requires the following specification (see e.g. 36):

$$Y_{it} = \alpha + \sum_{s=\tau}^{T} \beta_{1s} D_{sit} + \sum_{k=2}^{n} \beta_k X_{it} + \lambda_i + \theta_t + \varepsilon_{it} \tag{6}$$

In Eq. 6, the β_{1s} coefficients represent the DD estimates of ATT for each year s. The coefficients are comparable in interpretation to the estimates from our alternative estimators, which belong to the non-parametric, matched DD family of estimators.

Matching DD methods have some advantages over parametric DD in that they do not rely on parametric assumptions to control for differences in observable characteristics (30) and hence, are more likely to obtain like-for-like comparisons between treated grid cells and untreated ones. Matching estimators allow for arbitrary heterogeneity in the treatment effect compared to parametric approaches where the heterogeneity must be specified explicitly (37). As discussed in the following sub-section, while differencing accounts for unobservable fixed effects in DD estimators, under the identifying assumptions matching ensures that the heterogeneous trend in the transitory component, ϵ_{it}, is balanced between treatment and control groups (see e.g. (24)). Matching methods have been used to evaluate other area-based conservation policies due to these properties (27, 28, 38, 39). One typical matching approach matches on the propensity score: the probability of a unit being in the treated or untreated group. We use caliper one-to-one propensity score matching so that each Moratorium cell is matched with a similar non-Moratorium cell on the basis of the likelihood of being in the Moratorium. While propensity score matching does rely on parametric assumptions in estimating the propensity score, the estimation of the treatment effect is otherwise non-parametric, so we maintain the distinction between parametric and non-parametric (matching) DD approaches in what follows.

Propensity score matching DD conditions the differences in forest cover on the propensity score $P(X)$ to remove bias in levels and trends from confounding variables (24, 30, 40) under the assumption of selection on observables: d_i is independent of (Y_0, Y_1) when outcomes are conditioned on $P(X)$. Suppose that T is our final post treatment period, then together with the parallel trends assumption:

$$\begin{aligned} ATT_{DD,T} &= E_{P(X)}\left[E\left[Y_{1,T} - Y_{1,T_1} | d_i = 1, P\left(X_{T_m}\right)\right] - E\left[Y_{0,T} - Y_{0,T_1} | d_i = 1, P\left(X_{T_m}\right)\right]\right] & [7] \\ &= E\left[Y_{1T} - Y_{0T_1} | d_i = 1\right] & [8] \end{aligned}$$

where T ranges from 2012-2018, reflecting the different time-horizons over which the Moratorium's impact is estimated, $T_1 = 2011$ is the treatment year, and $T_m < T_1$ is the pre-treatment year(s) from which the matching variables' data are taken. To estimate $ATT_{DD,T}$ we use one-to-one caliper propensity score matching on data Y_{it}^{M} from the Moratorium grid cell i, matched with data $Y_{it}^{j,NM}$ in cell j from the non-Moratorium grid-cells:

$$\widehat{ATT}_{DD,T} = \frac{1}{N_M} \sum_{i} I_0 \left[\left[Y_{i,T}^{M} - Y_{i,T_1}^{M}\right] - \left[Y_{i,T}^{j,NM} - Y_{i,T_1}^{j,NM}\right]\right] \tag{9}$$

where I_0 is an indicator variable that is equal to 1 if a grid cell i in the Moratorium has a counterfactual grid cell j in the non-Moratorium area whose propensity scores p_i and p_j fall within the caliper:

$$|p_i - p_j| < \epsilon$$

where ε is a predetermined distance in propensity score space. The caliper defines the set of one-to-one matches from the non-Moratorium area, $C(i)$, such that $j \in C(i)$. Therefore, $I_0 = I\left(\min |p_i - p_j| : |p_i - p_j| < \epsilon\right)$. Our large dataset allows us to overcome the chief concern in the literature on optimal caliper choice: the loss of sample size with increased precision (41).

We use a precise caliper of 0.0001 which leads to a small percentage of observations being dropped where Moratorium cells do not have matches within this caliper. These are relatively low-quality matches and do not appear in $C(i)$. In the process of model selection, described in the following sub-section, we show the results are in any event insensitive to the choice of a wider caliper of 0.01. The remaining matched sample is of size N_M. The average of the counterfactual matched outcome is taken in case of ties in the non-Moratorium group. Our process of model selection suggests that the parallel trends assumption, which underpins the identification of the treatment effect when using the DD estimator, may not hold. Triple Difference estimators (DDD) are argued to have weaker identification assumptions than DD estimators, e.g. (42, p.11), and can be used as a robustness check or corrective measure (25, 42). We use DDD as corrective measure following (24)). Our DDD estimator takes the form:

$$\begin{aligned}\widehat{ATT}_{DDD,T} &= \frac{1}{N_M}\sum_i I_0\left\{\left[\left[Y^M_{i,T}-Y^M_{i,T_1}\right]-\left[Y^{j,NM}_{i,T}-Y^{j,NM}_{i,T_1}\right]\right]\right.\\ &\left.-\left(\left[Y^M_{i,T_1'}-Y^M_{i,T_0}\right]-\left[Y^{j,NM}_{i,T_1'}-Y^{j,NM}_{i,T_0}\right]\right)\left(\frac{T-T_1}{T_1'-T_0}\right)\right\} \qquad [10]\end{aligned}$$

where the second line reflects the correction for the non-parallel pre-treatment trends between T_0 and T_1' with a correction $\left(\frac{T-T_1}{T_1'-T_0}\right)$ to adjust the trend-correction for potentially different pre- and post-treatment time horizons.

The additional adjustment in the DDD estimator identifies the treatment effect in the event that the expected divergence of trends between the Moratorium and non-Moratorium areas is linear in time and the conditioning variables include pre-treatment outcomes including the year immediately prior to the treatment, in our case 2010. To demonstrate, leaving the conditioning on observable characteristics implicit, in a typical DD framework the ATT_{DD} is non-parametrically identified as follows:

$$\begin{aligned}& E\left[\Delta_{T,T_1}Y_{it}|D=1\right]-E\left[\Delta_{T,T_1}Y_{it}|D=0\right]\\ =\;& ATT_{DD,T,T_1}+E\left[\Delta_{T,T_1}u_{1it}|d_i=1\right]-E\left[\Delta_{T,T_1}u_{0it}|d_i=0\right] \qquad [11]\end{aligned}$$

where Y_{it} are the observable data, Δ_{T,T_1} represents the change operator between the period T and treatment period T_1, $ATT_{DD,T,T_1}=E\left[\Delta_{T,T_1}Y_{i1t}-\Delta_{T,T_1}Y_{i0t}\right]$ (and as above Y_{ikt} represent the potential outcomes for treated: $k=1$, and untreated: $k=0$ grid cells), and the last two terms in Eq. (11) are the trends in the unobservable characteristics in the treated and untreated states, which sum to zero if the parallel trends assumption holds.

Placebo tests are necessary to test whether or not the pre-trends are parallel in all cases for the simple DD estimator even when matching on several pre-treatment outcomes. If they are not parallel then this would imply that the DD estimator over the time horizon T_1 to T is potentially contaminated by a diverging trend in unobservables: the difference between the last two terms of Eq. 11. To try and resolve this issue, which could be problematic over long-time horizons, the DDD approach (proposed by (24)) could be adopted. The main identification assumptions are twofold. First, the non-parallel trend is linear: $E\left[\Delta_t u_{1it}|d_i=1\right]-E\left[\Delta_t u_{0it}|d_i=0\right]=\gamma t$. Second, the trend holds for all t and is unaffected by the treatment. With these assumptions, the confounding term in Eq. 11 becomes $\gamma\left(T-T_1\right)$. For pre-treatment time periods T_0 to T_1' the DD estimator in Eq. 11 becomes:

$$\begin{aligned}& E\left[\Delta_{T_1',T_0}Y_{it}|d_i=1\right]-E\left[\Delta_{T_1',T_0}Y_{it}|d_i=0\right]\\ =\;& ATT_{DD,T_1',T_0}+E\left[\Delta_{T_1',T_0}u_{1it}|d_i=1\right]-E\left[\Delta_{T_1',T_0}u_{0it}|d_i=0\right]\\ =\;& \gamma\left(T_1'-T_0\right) \qquad [12]\end{aligned}$$

because $ATT_{DD,T_1',T_0}=0$ in the pre-treatment phase. This expression is estimated using propensity score matching DD in the pre-treatment period for Moratorium and non-Moratorium areas. Having conditioned on pre-treatment outcomes in the matching routine, a trend correction is required for the period T_1 to T, which requires a conversion factor: $\left(\frac{T-T_1}{T_1'-T_0}\right)$ to relate the estimate in Eq. 12 to the required treatment effect in Eq. 10. This is a minor augmentation of the DDD approach proposed in (25). The years used in the main analysis were $T_0=2004$, $T_1'=2010$, $T_1=2011$ and $T=$ endpoint year: 2012-2018. For the placebo tests the placebo treatment year is 2005 and the pre- and post-treatment periods considered are, respectively, 2000-2004 and 2005-2010, with sensitivity using 2005-2011.

Model selection and balance tests. We now explain our empirical approach and the motivation for the choice of estimator. Our *model selection* process has four steps: (1) a comparison of non-parametric and parametric estimators; (2) placebo and falsification tests in time; (3) the response to the placebo tests in time; and, (4) placebo and falsification tests in space, as a final check on the selected estimator for parallel trends and the comparability of treatment and control areas.

Step 1: Parametric versus non-parametric estimators. Although ATT_{DD} is non-parametrically *identified* above, both parametric and non-parametric estimators can be used to *estimate* ATT_{DD}. Each controls for time invariant unobservables and each shares the following identification assumptions: (i) selection on observables; and, (ii) parallel trends in unobservable characteristics. Parametric estimators are typically less well-suited to dealing with individual heterogeneity in estimating treatment effects

(43). In the difference-in-differences framework, the parallel trends assumption is often argued to be more likely to hold when treated units are closely matched to non-treated (43) and experimental-empirical evidence often supports this view (e.g. 44). Furthermore, parametric approaches to conditioning on observable characteristics often place restrictive assumptions on functional forms of the relationship between conditioning variables and outcome variables, which are absent from non-parametric approaches and their estimation of treatment effects. Since these shortcomings can introduce bias in the estimates, we have an *a priori* preference for non-parametric estimates, among which we include propensity score matching. Nevertheless, matching estimators can also be subject to biases of their own (see e.g. (24, 45)). To transparently investigate potential differences between estimators, we undertake some exploratory sensitivity analysis of different estimators. We estimate parametric DD models of the form described in Eq. 5 using a fixed-effects estimator. We compare these estimates to a propensity score, one-to-one caliper, matched DD estimator (NP-DD). To rule out the possibility that the balance in the sample of observable characteristics between Moratorium and non-Moratorium areas causes differences between the parametric and non-parametric estimates, we use the same matched sample for the parametric DD estimation as is used for matched DD estimators (see (30)). We then undertake some basic sensitivity analysis on the non-parametric estimators by relaxing the precision of the matching procedure in two ways: (i) widening the caliper; and, (ii) sampling matches without replacement. We then compare the results with the parametric estimates and draw tentative conclusions.

Results. Table S35 presents the results (the ATT for 2017) of this comparative analysis. The results do not undermine, and rather strengthen, our *a priori* preference for non-parametric matching estimators. The parametric estimators (P-1 to Conley-HAC 4) are based on Eq. 6. Table S35 reports the results of these specifications. P-1 controls for grid-cell (λ_i) and year (θ_t) fixed effects. Model P-2 also controls for district-by-year fixed effects. Standard errors are clustered at the district level. These interaction effects capture heterogeneous trends by district. Models Conley 1 to Conley 4 are based on the same estimating equation as models P-1 and P-2, but employ Conley standard errors with respectively 10, 20, 50 and 100km thresholds for spatial autocorrelation decay. Specifications labelled Conley-HAC 1 to Conley-HAC 4 augment these latter models with Newey-West corrections for heteroskedasticity and temporal autocorrelations (10 lags). Comparing estimators P-1 and P-2, we find that the parametric DD is sensitive to whether or not district-by-year interaction effects are included in the estimation. The estimated ATT for P-1 is 3.76, whereas with district-by-year fixed effects the estimate drops by almost 80% to 0.79. Models Conley 1 to Conley-HAC 4 differ from P-2 exclusively with respect to the standard errors around the point estimates. We find that for thresholds of spatial autocorrelation decay below 20Km, Conley and Conley-HAC standard errors are smaller than those clustered at the district level; these are similar in magnitude to Conley and Conley-HAC standard errors using a 50km radius around observational units, while employing a 100km radius results in larger standard errors and slightly lower statistical significance. Nonetheless, the parametric results remain strongly significant for any standard error specification we deploy. The sensitivity of the ATT's magnitude to the inclusion of district-by-year interactions most likely reflects that concessions, and land uses in general, are licensed and administered at the district level and these administrative processes may differ across districts, causing trends to diverge in a way that is correlated with the location of the Moratorium. This suggests that district-level trends should be controlled for when estimating treatment effects.

Rather than control for this possibility in a parametric model, we use a matching estimator which matches on grid-level characteristics. The matching variables explicitly capture important differences between the Moratorium and non-Moratorium grid cells, their dynamics and suitability for future concessions. We use pre-treatment values of: distance to concessions (palm oil, timber and logging), distance to roads and cities, population (2005 and 2010), forest cover for each year from 2005 to 2010, elevation, slope, peat depth and above-ground carbon stock in the year 2000. With regard to the heterogeneous trends that were shown to be an important factor in the parametric estimator, we control for heterogeneous pre-treatment trends by matching on pre-treatment trends in forest cover and population for several pre-treatment years: 2005-2010 for forest cover, 2005 and 2010 for population. We prefer this approach to the simple inclusion of district-by-year fixed-effects since the approach in principle controls for more sources of heterogeneous trends due to conditioning on other matching variables.

Comparing model P-2 with the propensity score matched result from the main analysis, NP-DD (caliper = 0.0001), we find that the NP-DD estimates are almost identical: NP-DD = 0.81 and P-2 = 0.79. Table S43 shows the extensive list of matching variables that underpin the NP-DD estimate, which help to control for heterogeneity. When the matches are made less precise by obtaining matches without replacement (NP-1), the matching ATT decreases significantly: NP-1 = 0.36. NP-1, however, drops ten times the number of observations (> 70,000) in the process, and so is less favourable. Widening the caliper from 0.0001 to 0.01 (NP-2) drops fewer observations (2 in total), but this has little effect on the estimates: NP-2 = 0.81, as does removing the caliper altogether (NP-3), which is equivalent to nearest neighbour matching: NP-3 = 0.81. With no caliper and no replacement (NP-4), estimates increase towards the potentially problematic parametric estimates that do not control for district-by-year effects: NP-4 = 1.6. From these comparisons we conclude that the matching estimates are sensitive only to extreme reductions in precision of the matching (no replacement or no caliper), and there are some reasons to prefer the NP-DD estimator.

While these comparisons of estimators are exploratory, we prefer the propensity score matching estimator because it allows arbitrary heterogeneity in the treatment effect and closely matches treatment and control cells on the basis of pre-treatment trends, thereby accommodating heterogeneous pre-trends at district or indeed other levels, without imposing any additional structure on the estimation process. It is possible that controlling for district-by-year fixed effects in the parametric model may attenuate the estimate of the treatment effect by controlling for district level post-treatment responses, which would be a legitimate channel for the Moratorium being successful. Furthermore, linear parametric approaches to causal estimation also have been shown to be problematic in some multi-period cases (46). We therefore pursue a variety of non-parametric

matching estimators to estimate the impact of the Moratorium while noting that the appropriate parametric approaches (P-2 to Conley-HAC 4) provide estimates of essentially the same magnitude, albeit slightly lower. We then subject our preferred estimator to rigorous robustness testing of the matching approach algorithm and identification assumptions.

Step 2: Placebo tests in time. Our model selection approach turns to the identification assumptions of the DD estimator, namely the parallel trends assumption. Using the preferred non-parametric matched DD estimates of the treatment effect of the Moratorium for peatland and dryland forest, we attempt to empirically falsify the parallel trends assumption. To do this we undertake two distinct temporal placebo tests. The relevant time periods for the placebo tests can be seen in Fig. S6, which reproduces the trends for non-concession dryland grid cells for the period 2000-2018, from Fig. 1.

The first placebo test, Placebo Test 1, maintains the one-to-one matches between the Moratorium grid cells and the non-Moratorium grid cells that were used in the original DD estimates shown in Fig. 1 of the main text. In this case, matching variables are drawn from the period between T_0 to $T_{1'}$ in Fig. S6. These cells are then subjected to a placebo Moratorium in 2005 rather than the actual Moratorium in 2011. We use the difference in forest cover post- and pre-placebo treatment, respectively, the period between 2000 and T_0, and T_0 to $T_{1'}$, in Fig. S6, and estimate the placebo treatment effect ATT_{DDP}. This tests how successful the strategy of matching on pre-treatment outcomes in 2005-2010 is in ensuring, on average, parallel trends between Moratorium and non-Moratorium grid cells. The null hypothesis is that these pre-treatment controls ensure that the parallel trends assumption cannot be falsified and hence, we ought not to observe a significant effect of the placebo treatment. The alternative hypothesis is that parallel trends assumption can be falsified despite conditioning on outcomes between 2005-2010. Placebo Test 1 is motivated by (24, 25), who showed that matching on pre-treatment outcomes as a means of obtaining parallel pre-trends can sometimes fail and actually introduce bias. We match on several pre-treatment lags of the outcome variable, and so our expectation was that the test would fail to reject the null and hence, fail to falsify the parallel trends assumption.

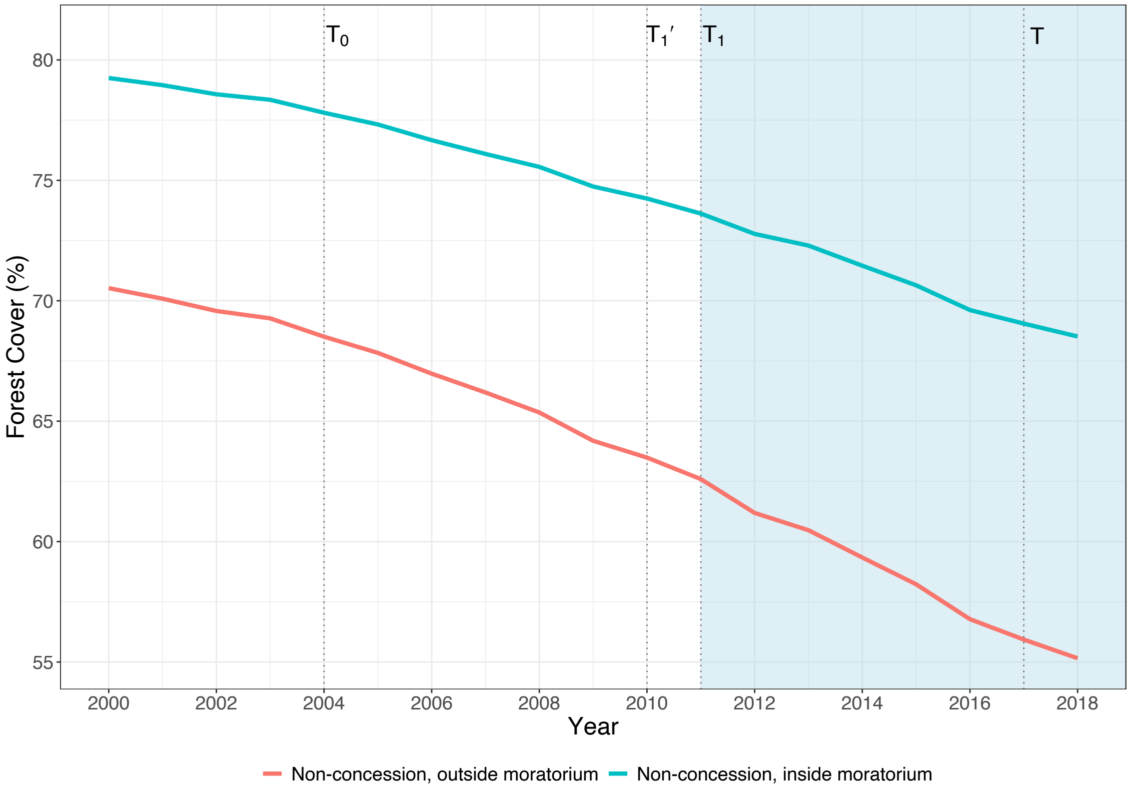

Fig. S6. Forest cover trends inside and outside the Moratorium, 2005-2018: non-concession dryland grid cells.

The second placebo test in time, Placebo Test 2, is a more traditional placebo test which shifts the entire analysis in time. That is, matching takes place on pre-placebo data (2000 to T_0) rather than on pre-(real) treatment data (T_0 to $T_{1'}$) as in Placebo Test 1. Again, the null hypothesis is that the parallel trends assumption of the matching estimator is not falsified. Both placebo tests are undertaken on each forest type. Together, these placebo tests can also be seen in light of the concerns raised by (47) about regression to the mean when matching on pre-treatment outcomes.

Results. The placebo/falsification tests indicate that the parallel trends assumption fails in some cases for the DD estimator. Tables S36-S40 report the results of all temporal placebo tests for dryland and peatland. For dryland areas, Placebo Test 1 yields a positive treatment effect in some cases (See Table S36). For stringency we undertake sensitivity on the definition of forested land, at the grid-cell scale, to be included in the analysis ranging from all land (untrimmed) to 30% forest cover in the base year, 2005, to 60% forest cover in 2005. Our central results use the unrestricted definition. In all cases but the 60% definition, we reject the null of Placebo Test 1. On the other hand, Placebo Test 2 is unable to falsify the parallel trends assumption, with well-defined zero impacts estimated across all three of our definitions of forest cover.

For peatland areas, Placebo Test 1 fails to reject the null hypothesis and falsify the parallel trends assumption, suggesting that matching on pre-treatment outcomes may be sufficient for identification. However, Placebo Test 2 rejects the null, with a statistically significant and negative effect of the placebo in 2005. This suggests that the parallel trends assumption may not hold in general for peatland. The failure of either of the placebo tests for dryland and peatland suggests remedial action is necessary.

Step 3: Response to placebo tests in time. We now argue that NP-DDD estimator shown in Eq. 10, which makes a correction in such cases, is therefore preferred. Rejection of the null hypothesis of the Placebo Test 1 is a sign that controlling for pre-treatment variables may have failed to ensure parallel pre-trends between Moratorium and non-Moratorium grid cells. Trends in unobservables diverge post-placebo and therefore are likely to do so post-treatment in the main analysis, when the real treatment happens in 2011 (T_1). Rejection of Placebo Test 2 has a similar implication: falsification of the parallel trends assumption. While often framed as a *test* of the parallel trends assumption, these tests are only indicative of unobservable phenomena. Failure to reject the null is an encouraging sign, rejection is suggestive of a need for remedial measures.

Following (24, 25), falsification can be ameliorated by a triple differences (DDD) approach that removes the pre-treatment difference in trends from the NP-DD estimate. Formally, this estimator (NP-DDD) is given by Eq. 10, where the last terms in square brackets represents the difference in the pre-trend between Moratorium and non-Moratorium areas between 2005 and 2010 at the matched grid-cell level. In Eq. 12, the assumption of a linear trend in the divergence of parallel trends is assumed, and the effect of this trend is corrected for the time horizon of the treatment effect being estimated (the choice of T in Fig. S6). This specification is an adaptation to the proposal by (25) and its application to deforestation in (24).

Results. In practice, the NP-DDD estimator in Eq. 10 requires estimating and removing an adjusted (for time horizon) version the estimated ATT in the placebo test. For instance, in Table S36 we see that 0.181 of the estimated effect in the main NP-DD estimate (untrimmed) of effect of the Moratorium may well arise because of non-parallel trends. The NP-DDD estimator simply removes this estimated effect, adjusting for the different time-horizon, from the NP-DD estimate for each year T in which it is estimated. Finally, we perform a third placebo test in time, Placebo Test 3, which aims at establishing whether the DDD methodology identifies a positive effect of the Moratorium on dryland cells when it is applied over the 2000-2010 (2011) placebo interval, with placebo treatment set to start in 2005. For the DDD estimator, we fail to reject the null of parallel trends in the cases where DD rejected the null. Our main results are derived using the NP-DDD estimator implemented in this way, and Fig. 2 compares the results from NP-DD and NP-DDD estimators graphically to illustrate the differences.

Step 4: Placebo tests in space. Finally, as a final check of the robustness of the NP-DDD estimator, we undertake another placebo-falsification test. We run spatial placebo tests using untreated cells in the Moratorium (treated) area as the placebo treatment cells. This spatial placebo test exploits a loophole in the Moratorium rules, which allows for continued deforestation in pre-2011 (pre-Moratorium) concession areas irrespective of whether the concessions are in the Moratorium or not. Since grid cells in concessions in the Moratorium area should be unaffected by the Moratorium, they are obvious candidates for Moratorium placebo treatment cells.

The null hypothesis of the spatial placebo test is that the NP-DDD estimator is well-identified: the parallel trends assumption cannot be falsified and there is no impact of the Moratorium on Moratorium concession cells compared to non-Moratorium concession cells. The alternative hypothesis is that there remain some residual unobservable differences between the Moratorium and non-Moratorium areas that confound the estimate of the impact of the Moratorium. This would be the implication of a non-zero estimate of the Moratorium in the spatial placebo test. If the null hypothesis is rejected, it can be interpreted as a falsification of the parallel trends assumption. In principle, rejection of the null can inform a spatial triple differences DDD design to remove any deviation of parallel trends from the central NP-DDD estimate (31, 35, 48, 49). We run the spatial placebo test for all concession types, and also investigate the placebo for individual concession and forest types using the non-parametric (25) NP-DDD matching estimator of the treatment effect in 2011 (T_1). The empirical model can be thought of as Eq. 10, with the exception that the treatment and control data come from the concession cells in the Moratorium and concession cells in the non-Moratorium areas, instead of Moratorium and non-Moratorium non-concession forested areas.

Results. Tables S41-S42 show the results of these spatial placebo tests, all of which apply the NP-DDD estimator. Table S41 shows that for dryland areas, there are no differences in forest cover between Moratorium and non-Moratorium concession cells. Logging concessions show a negative effect, which is only statistically significant at the 10% level. For peatland areas, a significantly positive effect is detected for logging concessions only (Table S42), while for all concessions we fail to reject the null of the placebo test. We take these results to mean that on average, the NP-DDD is not susceptible to differences in trends due to unobserved differences between Moratorium and non-Moratorium areas.

With the propensity score, one-to-one, caliper matching NP-DDD estimator shown to satisfy the spatial placebo test, we undertake some additional checks and robustness analyses. First, we illustrate the success of our propensity score matching procedure at balancing the characteristics between Moratorium and non-Moratorium areas. We then test the robustness of the

results to different types of propensity score matching approaches and a non-propensity score matching procedure, a series of Coarsened Exact Matching estimates. Finally we test the Stable Unit Treatment Value Assumption (SUTVA).

Table S35. Results of model selection (endpoint 2017)

	ATT	AI SE	t-stat	p-value	Treated	Matched	Dropped (treated)
**Main Result**	0.813***	0.0990	8.186	0.000	160012	152118	7894
**NP-1**	0.357***	0.0856	4.170	0.000	160012	88120	71892
**NP-2**	0.81***	0.1130	7.166	0.000	160012	160010	2
**NP-3**	0.81***	0.1130	7.166	0.000	160012	160012	0
**NP-4**	1.58***	0.0629	25.126	0.000	160012	160012	0

	ATT	SE	t-stat	p-value	Treated	Matched	Dropped (treated)
**P-1**	3.760***	0.2509	14.987	0.000	160012	152118	7894
**P-2**	0.7854***	0.2612	3.007	0.003	160012	152118	7894
**Conley 1**	0.7854***	0.1769	4.440	0.000	160012	152118	7894
**Conley 2**	0.7854***	0.2106	3.729	0.000	160012	152118	7894
**Conley 3**	0.7854***	0.2630	2.986	0.003	160012	152118	7894
**Conley 4**	0.7854**	0.3211	2.446	0.014	160012	152118	7894
**Conley-HAC 1**	0.7854***	0.1803	4.356	0.000	160012	152118	7894
**Conley-HAC 2**	0.7854***	0.2135	3.679	0.000	160012	152118	7894
**Conley-HAC 3**	0.7854***	0.2653	2.960	0.003	160012	152118	7894
**Conley-HAC 4**	0.7854**	0.3230	2.432	0.015	160012	152118	7894

Notes: Abadie-Imbens (21) standard errors for all non-parametric specifications
(NP-1) No replacement, caliper width: 0.0001. 1-to-1 matching keeping ties.
(NP-2) Wider caliper width: 0.01. 1-to-1 matching with replacement, keeping ties.
(NP-3) No caliper, 1-to-1 matching with replacement, keeping ties.
(NP-4) No caliper, no replacement, 1-to-1 matching, keeping ties.
(P-1) Parametric DD, on matched sample from NP-1, year fixed effects, SEs clustered at the district level.
(P-2) Parametric DD, on matched sample from NP-1, year and district*year fixed effects, SEs clustered at the district level.
(Conley 1) Same as P-2, Conley SEs with 10km radius for spatial autocorrelation decay.
(Conley 2) Same as P-2, Conley SEs with 20km radius for spatial autocorrelation decay.
(Conley 3) Same as P-2, Conley SEs with 50km radius for spatial autocorrelation decay.
(Conley 4) Same as P-2, Conley SEs with 100km radius for spatial autocorrelation decay.
(Conley-HAC 1) Same as Conley 1, with Newey-West heteroskedasticity and autocovariance (HAC) correction (10 lags).
(Conley-HAC 2) Same as Conley 2, with Newey-West heteroskedasticity and autocovariance (HAC) correction (10 lags).
(Conley-HAC 3) Same as Conley 3, with Newey-West heteroskedasticity and autocovariance (HAC) correction (10 lags)
(Conley-HAC 4) Same as Conley 4, with Newey-West heteroskedasticity and autocovariance (HAC) correction (10 lags).
$^{*}\ p < 0.1$, $^{**}\ p < 0.05$, $^{***}\ p < 0.01$.

Table S36. Placebo test in time 1: DD dryland, 2005-2010 outcome, same matching

	ATT	AI SE	t-stat	p-value	Treated	Matched	Dropped (treated)
**Untrimmed**	0.181**	0.0840	2.158	0.031	160012	152118	7894
**30%**	0.203**	0.0930	2.175	0.030	144501	136472	8029
**60%**	0.149	0.1010	1.468	0.142	127375	119284	8091

Notes: Abadie-Imbens (21) standard errors.
Maximum caliper width: 0.0001. 1-to-1 matching with replacement, keeping ties.
$^{*}\ p < 0.1$, $^{**}\ p < 0.05$, $^{***}\ p < 0.01$.

Table S37. Placebo test in time 2: DD dryland, 2005-2010 outcome, matching up to 2004

	ATT	AI SE	t-stat	p-value	Treated	Matched	Dropped (treated)
**Untrimmed**	-0.048	0.0820	-0.580	0.562	160012	152408	7604
**30%**	-0.038	0.0920	-0.409	0.682	144501	136304	8197
**60%**	-0.064	0.1000	-0.640	0.522	127375	119677	7698

Notes: Abadie-Imbens (21) standard errors.
Maximum caliper width: 0.0001. 1-to-1 matching with replacement, keeping ties.
$^{*}\ p < 0.1$, $^{**}\ p < 0.05$, $^{***}\ p < 0.01$.

Table S38. Placebo test in time 1: DD peatland, 2005-2010 outcome, same matching

	ATT	AI SE	t-stat	p-value	Treated	Matched	Dropped (treated)
**Untrimmed**	0.05	0.0930	0.533	0.594	95154	91024	4130
**30%**	-0.06	0.1010	-0.593	0.553	86897	82832	4065
**60%**	-0.047	0.1090	-0.433	0.665	77777	73554	4223

Notes: Abadie-Imbens (21) standard errors.
Maximum caliper width: 0.0001. 1-to-1 matching with replacement, keeping ties.
$^{*}\ p < 0.1$, $^{**}\ p < 0.05$, $^{***}\ p < 0.01$.

Table S39. Placebo test in time 2: DD peatland, 2005-2010 outcome, matching up to 2004

	ATT	AI SE	t-stat	p-value	Treated	Matched	Dropped (treated)
**Untrimmed**	-0.39***	0.0890	-4.367	0.000	95154	91132	4022
**30%**	-0.486***	0.0970	-4.998	0.000	86897	82852	4045
**60%**	-0.496***	0.1050	-4.741	0.000	77777	73430	4347

Notes: Abadie-Imbens (21) standard errors.
Maximum caliper width: 0.0001. 1-to-1 matching with replacement, keeping ties.
$^{*}\ p < 0.1$, $^{**}\ p < 0.05$, $^{***}\ p < 0.01$.

Table S40. Placebo test in time 3: DDD dryland, placebo treatment in 2005, same matching

Endpoint	ATT	AI SE	t-stat	p-value	Treated	Matched	Dropped (treated)
**2010**	-0.018	0.0920	-0.191	0.848	160012	152118	7894
**2011**	0.049	0.1060	0.465	0.642	160012	152118	7894

Notes: Abadie-Imbens (21) standard errors.
Maximum caliper width: 0.0001. 1-to-1 matching with replacement, keeping ties.
$^{*}\ p < 0.1$, $^{**}\ p < 0.05$, $^{***}\ p < 0.01$.

Table S41. Spatial placebo: Dryland, 2011-2017 outcome

	ATT	AI SE	t-stat	p-value	Treated	Matched	Dropped (treated)
**All concessions**	-0.33	0.3000	-1.099	0.272	8577	7702	875
**Palm Oil**	0.909	0.7750	1.173	0.241	2318	2249	69
**Timber**	0.86	0.9650	0.890	0.373	1183	1176	7
**Logging**	-0.68*	0.4000	-1.697	0.090	5193	5188	5

Notes: Abadie-Imbens (21) standard errors.

Maximum caliper width: 0.0001. 1-to-1 matching with replacement, keeping ties.

* $p < 0.1$, ** $p < 0.05$, *** $p < 0.01$.

Table S42. Spatial placebo: Peatland, 2011-2017 outcome

	ATT	AI SE	t-stat	p-value	Treated	Matched	Dropped (treated)
**All concessions**	0.485	0.4090	1.185	0.236	6930	6604	326
**Palm Oil**	0.156	0.5960	0.262	0.793	4553	4553	0
**Timber**	0.437	1.7480	0.250	0.803	643	640	3
**Logging**	1.067*	0.5490	1.943	0.052	1765	1762	3

Notes: Abadie-Imbens (21) standard errors.

Maximum caliper width: 0.0001. 1-to-1 matching with replacement, keeping ties.

* $p < 0.1$, ** $p < 0.05$, *** $p < 0.01$.

Balance tests for one-to-one caliper matching. The descriptive statistics in Tables S27-S34 show that in the raw data there are large differences between the Moratorium and non-Moratorium areas. Matching variables are selected on the basis of these differences, shown in Table S43-S54 by forest type and land use. For example, Table S43 describes the successful results of the matching procedure for dryland forest areas, and the success in balancing the treatment and control grid cells is similar across all of the remaining matching tables. In Table S43, forest cover in the six years prior to the treatment (2011) in the matched sample has a mean difference of no more than 0.5 ha compared to approximately 15 ha before matching. The standardised difference is no more than 0.55. These small mean differences are also complemented by two comparisons of the distributions of these characteristics: the Kolmogorov Smirnov test and a test of differences in the cumulative distribution function (eCDF). Both reveal highly balanced control variables in the matched sample, with small differences in the CDFs of the variables. Our conclusion is that the matching procedure performs well.

Table S43. Dryland forest, untrimmed, matching up to 2010

	Unmatched					Matched				
	T	C	Diff	KS	eCDF	T	C	Diff	KS	eCDF
**Forest 2005**	109.08	95.49	33.26	0.18	0.11	108.37	108.56	-0.44	0.03	0.02
**Forest 2006**	108.15	94.25	33.54	0.19	0.11	107.42	107.51	-0.22	0.04	0.02
**Forest 2007**	107.33	93.12	33.85	0.19	0.11	106.60	106.66	-0.16	0.03	0.02
**Forest 2008**	106.56	91.92	34.49	0.19	0.11	105.82	105.90	-0.19	0.04	0.02
**Forest 2009**	105.40	90.23	35.12	0.19	0.12	104.64	104.69	-0.11	0.04	0.02
**Forest 2010**	104.68	89.23	35.39	0.19	0.12	103.92	103.92	-0.00	0.04	0.02
**Elevation**	470.04	207.62	96.65	0.47	0.28	457.29	459.78	-0.93	0.07	0.03
**Slope**	13.14	6.15	104.81	0.47	0.29	12.85	13.12	-4.16	0.08	0.03
**Distance roads**	35.15	12.34	70.50	0.42	0.28	33.67	35.51	-5.79	0.09	0.05
**Distance cities**	1588.08	579.06	75.26	0.43	0.26	1512.51	1492.76	1.53	0.14	0.04
**Distance palm oil**	102.35	167.75	-46.31	0.20	0.07	103.94	102.26	1.16	0.06	0.04
**Distance timber**	123.84	128.03	-2.91	0.10	0.05	121.11	103.41	12.39	0.07	0.04
**Distance logging**	87.26	230.05	-82.59	0.32	0.20	90.53	92.81	-1.29	0.06	0.03
**AGC**	157.82	92.17	171.62	0.57	0.33	157.02	155.03	5.11	0.02	0.01
**Population 2005**	17.30	328.56	-229.96	0.49	0.28	18.06	21.38	-2.39	0.07	0.04
**Population 2010**	14.92	328.89	-247.47	0.50	0.29	15.60	18.91	-2.55	0.06	0.04

Notes: 1000 bootstrap samples. T: treated group. C: control group. Diff: absolute standardised difference.
KS: Kolmogorov-Smirnov test statistic. eCDF: mean difference in empirical CDFs.

Table S44. Dryland forest, untrimmed, matching up to 2004

	Unmatched					Matched				
	T	C	Diff	KS	eCDF	T	C	Diff	KS	eCDF
**Forest 2000**	111.83	99.34	31.42	0.17	0.09	111.20	111.46	-0.65	0.03	0.01
**Forest 2001**	111.41	98.71	31.84	0.18	0.10	110.78	110.98	-0.51	0.03	0.01
**Forest 2002**	110.87	97.99	32.19	0.18	0.10	110.23	110.41	-0.46	0.03	0.01
**Forest 2003**	110.55	97.55	32.40	0.18	0.10	109.89	110.05	-0.39	0.03	0.02
**Forest 2004**	109.78	96.46	32.91	0.18	0.11	109.11	109.21	-0.25	0.03	0.02
**Elevation**	470.04	207.62	96.65	0.47	0.28	458.01	460.68	-0.99	0.07	0.03
**Slope**	13.14	6.15	104.81	0.47	0.29	12.86	13.14	-4.22	0.07	0.03
**Distance roads**	35.15	12.34	70.50	0.42	0.28	33.73	35.50	-5.56	0.09	0.05
**Distance cities**	1588.08	579.06	75.26	0.43	0.26	1514.88	1501.71	1.02	0.14	0.04
**Distance palm oil**	102.35	167.75	-46.31	0.20	0.07	103.89	102.83	0.74	0.07	0.04
**Distance timber**	123.84	128.03	-2.91	0.10	0.05	120.99	104.20	11.76	0.07	0.04
**Distance logging**	87.26	230.05	-82.59	0.32	0.20	90.38	93.30	-1.66	0.06	0.04
**AGC**	157.82	92.17	171.62	0.57	0.33	157.04	154.93	5.41	0.02	0.01
**Population 2005**	17.30	328.56	-229.96	0.49	0.28	18.04	21.40	-2.43	0.07	0.04
**Population 2010**	14.92	328.89	-247.47	0.50	0.29	15.58	19.08	-2.70	0.06	0.04

Notes: 1000 bootstrap samples. T: treated group. C: control group. Diff: absolute standardised difference.
KS: Kolmogorov-Smirnov test statistic. eCDF: mean difference in empirical CDFs.

Table S45. Dryland forest, $>$ 30% forest cover in 2005, matching up to 2010

	Unmatched					Matched				
	T	C	Diff	KS	eCDF	T	C	Diff	KS	eCDF
**Forest 2005**	118.92	110.41	29.53	0.17	0.10	118.46	118.82	-1.22	0.03	0.01
**Forest 2006**	117.94	109.01	29.87	0.18	0.10	117.46	117.76	-1.01	0.03	0.01
**Forest 2007**	117.06	107.72	30.24	0.18	0.10	116.57	116.84	-0.86	0.03	0.02
**Forest 2008**	116.22	106.32	31.14	0.18	0.10	115.73	115.99	-0.82	0.03	0.02
**Forest 2009**	114.97	104.38	31.96	0.18	0.11	114.47	114.68	-0.63	0.03	0.02
**Forest 2010**	114.20	103.21	32.28	0.18	0.11	113.68	113.83	-0.44	0.03	0.02
**Elevation**	471.68	208.82	96.79	0.47	0.28	457.97	461.10	-1.16	0.06	0.03
**Slope**	13.12	6.21	103.49	0.46	0.29	12.80	13.07	-4.11	0.07	0.02
**Distance roads**	36.34	12.81	72.16	0.42	0.28	34.81	36.66	-5.76	0.08	0.04
**Distance cities**	1648.19	611.43	76.28	0.43	0.26	1568.10	1552.27	1.20	0.13	0.04
**Distance palm oil**	98.81	161.36	-46.28	0.20	0.07	100.44	98.33	1.53	0.06	0.03
**Distance timber**	127.91	123.30	3.17	0.12	0.05	124.45	106.56	12.44	0.06	0.03
**Distance logging**	81.38	221.39	-84.92	0.31	0.19	84.70	87.03	-1.38	0.06	0.03
**AGC**	159.12	94.00	174.17	0.56	0.33	158.25	156.31	5.08	0.03	0.01
**Population 2005**	16.50	318.27	-221.71	0.48	0.28	17.31	20.49	-2.27	0.07	0.04
**Population 2010**	14.30	319.18	-240.29	0.50	0.29	15.03	18.28	-2.49	0.06	0.04

Notes: 1000 bootstrap samples. T: treated group. C: control group. Diff: absolute standardised difference.
KS: Kolmogorov-Smirnov test statistic. eCDF: mean difference in empirical CDFs.

Table S46. Dryland forest, $>$ 30% forest cover in 2005, matching up to 2004

	Unmatched					Matched				
	T	C	Diff	KS	eCDF	T	C	Diff	KS	eCDF
**Forest 2000**	121.18	113.93	26.19	0.16	0.07	120.79	121.12	-1.20	0.03	0.01
**Forest 2001**	120.81	113.34	26.89	0.16	0.08	120.41	120.71	-1.05	0.03	0.01
**Forest 2002**	120.34	112.64	27.49	0.17	0.09	119.92	120.20	-0.98	0.03	0.01
**Forest 2003**	120.06	112.23	27.82	0.17	0.09	119.63	119.89	-0.91	0.03	0.01
**Forest 2004**	119.47	111.26	28.84	0.17	0.09	119.03	119.23	-0.72	0.03	0.02
**Elevation**	471.68	208.82	96.79	0.47	0.28	457.34	462.50	-1.92	0.06	0.03
**Slope**	13.12	6.21	103.49	0.46	0.29	12.78	13.04	-3.90	0.07	0.02
**Distance roads**	36.34	12.81	72.16	0.42	0.28	34.70	36.79	-6.53	0.08	0.04
**Distance cities**	1648.19	611.43	76.28	0.43	0.26	1566.34	1551.51	1.13	0.13	0.04
**Distance palm oil**	98.81	161.36	-46.28	0.20	0.07	100.47	98.99	1.07	0.06	0.03
**Distance timber**	127.91	123.30	3.17	0.12	0.05	124.30	106.51	12.38	0.06	0.04
**Distance logging**	81.38	221.39	-84.92	0.31	0.19	84.79	87.60	-1.67	0.06	0.03
**AGC**	159.12	94.00	174.17	0.56	0.33	158.25	156.07	5.71	0.03	0.01
**Population 2005**	16.50	318.27	-221.71	0.48	0.28	17.33	20.61	-2.34	0.07	0.04
**Population 2010**	14.30	319.18	-240.29	0.50	0.29	15.04	18.43	-2.60	0.06	0.04

Notes: 1000 bootstrap samples. T: treated group. C: control group. Diff: absolute standardised difference.
KS: Kolmogorov-Smirnov test statistic. eCDF: mean difference in empirical CDFs.

Table S47. Dryland forest, $>$ 60% forest cover in 2005, matching up to 2010

	Unmatched					Matched				
	T	C	Diff	KS	eCDF	T	C	Diff	KS	eCDF
**Forest 2005**	126.61	121.58	25.40	0.16	0.09	126.37	126.41	-0.21	0.03	0.02
**Forest 2006**	125.74	120.27	25.92	0.16	0.09	125.48	125.45	0.12	0.03	0.02
**Forest 2007**	124.90	118.98	26.29	0.16	0.10	124.62	124.58	0.18	0.03	0.02
**Forest 2008**	124.08	117.55	27.39	0.16	0.10	123.78	123.71	0.28	0.03	0.02
**Forest 2009**	122.86	115.57	28.34	0.17	0.10	122.53	122.45	0.28	0.03	0.02
**Forest 2010**	122.09	114.34	28.80	0.17	0.10	121.74	121.64	0.40	0.03	0.02
**Elevation**	473.06	210.05	97.03	0.47	0.28	458.02	464.71	-2.49	0.05	0.02
**Slope**	13.11	6.28	102.41	0.46	0.29	12.76	13.00	-3.64	0.05	0.02
**Distance roads**	37.66	13.45	73.75	0.43	0.28	35.97	38.13	-6.68	0.07	0.04
**Distance cities**	1717.08	656.78	77.15	0.43	0.26	1634.30	1627.18	0.53	0.10	0.03
**Distance palm oil**	96.25	158.63	-47.90	0.20	0.07	97.88	95.02	2.14	0.05	0.02
**Distance timber**	133.23	120.82	8.43	0.13	0.06	129.22	110.48	12.86	0.05	0.02
**Distance logging**	76.03	214.49	-87.45	0.30	0.19	79.47	82.90	-2.11	0.05	0.02
**AGC**	160.50	95.94	176.78	0.56	0.33	159.58	157.33	6.03	0.03	0.01
**Population 2005**	15.77	303.70	-209.51	0.48	0.27	16.67	20.04	-2.37	0.07	0.04
**Population 2010**	13.75	305.33	-228.24	0.49	0.28	14.56	18.06	-2.66	0.06	0.04

Notes: 1000 bootstrap samples. T: treated group. C: control group. Diff: absolute standardised difference.
KS: Kolmogorov-Smirnov test statistic. eCDF: mean difference in empirical CDFs.

Table S48. Dryland forest, $>$ 60% forest cover in 2005, matching up to 2004

	Unmatched					Matched				
	**T**	**C**	**Diff**	**KS**	**eCDF**	**T**	**C**	**Diff**	**KS**	**eCDF**
**Forest 2000**	128.21	124.16	21.27	0.15	0.07	128.01	128.27	-1.38	0.02	0.01
**Forest 2001**	127.93	123.70	22.11	0.15	0.08	127.72	127.96	-1.25	0.02	0.01
**Forest 2002**	127.57	123.17	22.79	0.15	0.08	127.35	127.57	-1.15	0.02	0.01
**Forest 2003**	127.36	122.87	23.17	0.15	0.08	127.14	127.35	-1.08	0.03	0.01
**Forest 2004**	126.95	122.17	24.41	0.15	0.09	126.72	126.90	-0.94	0.03	0.01
**Elevation**	473.06	210.05	97.03	0.47	0.28	458.49	464.89	-2.39	0.05	0.02
**Slope**	13.11	6.28	102.41	0.46	0.29	12.76	13.05	-4.38	0.05	0.02
**Distance roads**	37.66	13.45	73.75	0.43	0.28	36.07	38.10	-6.26	0.07	0.04
**Distance cities**	1717.08	656.78	77.15	0.43	0.26	1639.50	1622.20	1.29	0.10	0.03
**Distance palm oil**	96.25	158.63	-47.90	0.20	0.07	97.79	94.64	2.36	0.05	0.02
**Distance timber**	133.23	120.82	8.43	0.13	0.06	129.49	108.89	14.13	0.05	0.02
**Distance logging**	76.03	214.49	-87.45	0.30	0.19	79.26	81.72	-1.52	0.05	0.02
**AGC**	160.50	95.94	176.78	0.56	0.33	159.64	157.50	5.73	0.03	0.01
**Population 2005**	15.77	303.70	-209.51	0.48	0.27	16.62	19.81	-2.26	0.07	0.04
**Population 2010**	13.75	305.33	-228.24	0.49	0.28	14.51	17.84	-2.53	0.06	0.04

Notes: 1000 bootstrap samples. T: treated group. C: control group. Diff: absolute standardised difference.
KS: Kolmogorov-Smirnov test statistic. eCDF: mean difference in empirical CDFs.

Table S49. Peatland forest, untrimmed, matching up to 2010

	Unmatched					Matched				
	T	C	Diff	KS	eCDF	T	C	Diff	KS	eCDF
**Forest 2005**	111.44	98.45	32.59	0.19	0.11	111.00	110.89	0.28	0.03	0.01
**Forest 2006**	110.66	97.15	33.45	0.19	0.11	110.20	110.09	0.27	0.03	0.02
**Forest 2007**	109.83	95.79	34.25	0.20	0.12	109.35	109.23	0.27	0.03	0.01
**Forest 2008**	108.98	94.38	35.08	0.20	0.12	108.47	108.37	0.25	0.03	0.01
**Forest 2009**	107.95	92.58	36.22	0.20	0.12	107.41	107.28	0.32	0.04	0.02
**Forest 2010**	107.09	91.37	36.48	0.20	0.12	106.53	106.37	0.38	0.04	0.02
**Elevation**	87.08	48.09	23.94	0.19	0.11	83.31	76.20	4.41	0.18	0.10
**Slope**	3.29	1.98	23.62	0.08	0.03	3.20	3.03	3.07	0.19	0.09
**Distance roads**	54.33	24.15	69.26	0.40	0.26	53.10	54.26	-2.67	0.15	0.09
**Distance cities**	1738.69	783.99	76.37	0.41	0.25	1693.34	1743.92	-4.08	0.13	0.04
**Distance palm oil**	59.65	77.76	-30.13	0.17	0.10	59.10	57.01	3.43	0.14	0.08
**Distance timber**	134.29	89.12	31.52	0.24	0.12	127.53	117.72	7.04	0.14	0.08
**Distance logging**	49.65	125.51	-118.54	0.24	0.13	50.68	49.37	2.00	0.12	0.06
**AGC**	119.68	68.44	97.41	0.43	0.26	117.36	118.00	-1.23	0.08	0.02
**Population 2005**	21.88	429.29	-214.34	0.41	0.25	22.79	26.85	-2.09	0.05	0.03
**Population 2010**	20.22	435.10	-208.19	0.42	0.26	21.07	25.34	-2.10	0.06	0.03

Notes: 1000 bootstrap samples. T: treated group. C: control group. Diff: absolute standardised difference.
KS: Kolmogorov-Smirnov test statistic. eCDF: mean difference in empirical CDFs.

Table S50. Peatland forest, untrimmed, matching up to 2004

	Unmatched					Matched				
	T	C	Diff	KS	eCDF	T	C	Diff	KS	eCDF
**Forest 2000**	114.26	102.69	30.14	0.17	0.09	113.86	113.73	0.34	0.05	0.02
**Forest 2001**	113.81	102.00	30.60	0.18	0.10	113.40	113.26	0.35	0.05	0.02
**Forest 2002**	113.30	101.26	31.02	0.18	0.10	112.87	112.73	0.37	0.05	0.02
**Forest 2003**	113.00	100.80	31.32	0.18	0.10	112.57	112.40	0.44	0.06	0.02
**Forest 2004**	112.22	99.60	32.06	0.19	0.11	111.77	111.63	0.36	0.05	0.02
**Elevation**	87.08	48.09	23.94	0.19	0.11	83.50	76.31	4.44	0.18	0.10
**Slope**	3.29	1.98	23.62	0.08	0.03	3.21	3.07	2.43	0.19	0.10
**Distance roads**	54.33	24.15	69.26	0.40	0.26	53.05	54.03	-2.25	0.15	0.09
**Distance cities**	1738.69	783.99	76.37	0.41	0.25	1695.90	1744.58	-3.92	0.13	0.04
**Distance palm oil**	59.65	77.76	-30.13	0.17	0.10	59.12	56.79	3.84	0.13	0.08
**Distance timber**	134.29	89.12	31.52	0.24	0.12	127.75	118.00	6.99	0.14	0.08
**Distance logging**	49.65	125.51	-118.54	0.24	0.13	50.62	49.34	1.97	0.12	0.06
**AGC**	119.68	68.44	97.41	0.43	0.26	117.45	118.25	-1.53	0.08	0.02
**Population 2005**	21.88	429.29	-214.34	0.41	0.25	22.77	26.83	-2.09	0.05	0.03
**Population 2010**	20.22	435.10	-208.19	0.42	0.26	21.04	25.30	-2.09	0.06	0.04

Notes: 1000 bootstrap samples. T: treated group. C: control group. Diff: absolute standardised difference.
KS: Kolmogorov-Smirnov test statistic. eCDF: mean difference in empirical CDFs.

Table S51. Peatland forest, $>$ 30% forest cover in 2005, matching up to 2010

	Unmatched					Matched				
	**T**	**C**	**Diff**	**KS**	**eCDF**	**T**	**C**	**Diff**	**KS**	**eCDF**
**Forest 2005**	120.39	112.14	29.15	0.18	0.10	120.12	119.96	0.55	0.05	0.02
**Forest 2006**	119.58	110.69	30.42	0.18	0.10	119.29	119.19	0.33	0.05	0.02
**Forest 2007**	118.71	109.15	31.58	0.19	0.11	118.39	118.31	0.27	0.05	0.02
**Forest 2008**	117.81	107.55	32.72	0.19	0.11	117.46	117.39	0.25	0.05	0.02
**Forest 2009**	116.72	105.52	34.27	0.19	0.12	116.34	116.25	0.28	0.05	0.02
**Forest 2010**	115.81	104.13	34.54	0.20	0.12	115.40	115.30	0.28	0.05	0.02
**Elevation**	90.29	48.98	24.89	0.20	0.11	86.65	80.08	3.99	0.17	0.10
**Slope**	3.38	2.02	24.20	0.09	0.04	3.29	3.16	2.38	0.19	0.10
**Distance roads**	56.35	25.50	69.90	0.41	0.26	55.09	55.87	-1.78	0.18	0.10
**Distance cities**	1800.73	830.62	77.52	0.41	0.25	1757.17	1798.41	-3.32	0.15	0.04
**Distance palm oil**	61.92	75.10	-22.25	0.19	0.11	61.42	58.80	4.37	0.13	0.07
**Distance timber**	141.70	88.66	36.58	0.26	0.13	134.88	126.11	6.20	0.13	0.08
**Distance logging**	48.35	120.26	-115.38	0.23	0.12	49.35	48.12	1.94	0.11	0.05
**AGC**	122.20	70.04	100.25	0.44	0.26	119.89	120.86	-1.86	0.08	0.02
**Population 2005**	19.57	414.06	-220.31	0.42	0.25	20.44	24.18	-2.04	0.06	0.03
**Population 2010**	18.33	419.06	-210.11	0.42	0.26	19.15	23.11	-2.03	0.06	0.03

Notes: 1000 bootstrap samples. T: treated group. C: control group. Diff: absolute standardised difference.
KS: Kolmogorov-Smirnov test statistic. eCDF: mean difference in empirical CDFs.

Table S52. Peatland forest, $>$ 30% forest cover in 2005, matching up to 2004

	Unmatched					Matched				
	T	C	Diff	KS	eCDF	T	C	Diff	KS	eCDF
**Forest 2000**	122.51	115.81	24.79	0.16	0.08	122.28	122.34	-0.22	0.05	0.02
**Forest 2001**	122.19	115.19	25.78	0.17	0.09	121.95	122.01	-0.22	0.05	0.02
**Forest 2002**	121.78	114.52	26.55	0.17	0.09	121.53	121.59	-0.22	0.06	0.02
**Forest 2003**	121.55	114.11	27.05	0.17	0.09	121.29	121.35	-0.20	0.06	0.02
**Forest 2004**	120.99	113.14	28.18	0.18	0.10	120.71	120.77	-0.20	0.06	0.02
**Elevation**	90.29	48.98	24.89	0.20	0.11	86.33	77.35	5.46	0.16	0.09
**Slope**	3.38	2.02	24.20	0.09	0.04	3.28	3.07	3.77	0.19	0.09
**Distance roads**	56.35	25.50	69.90	0.41	0.26	55.10	56.22	-2.53	0.18	0.10
**Distance cities**	1800.73	830.62	77.52	0.41	0.25	1757.06	1816.16	-4.75	0.15	0.04
**Distance palm oil**	61.92	75.10	-22.25	0.19	0.11	61.45	58.83	4.37	0.13	0.07
**Distance timber**	141.70	88.66	36.58	0.26	0.13	134.73	125.05	6.85	0.13	0.08
**Distance logging**	48.35	120.26	-115.38	0.23	0.12	49.33	48.09	1.95	0.11	0.05
**AGC**	122.20	70.04	100.25	0.44	0.26	119.86	120.56	-1.35	0.08	0.02
**Population 2005**	19.57	414.06	-220.31	0.42	0.25	20.44	24.22	-2.06	0.05	0.03
**Population 2010**	18.33	419.06	-210.11	0.42	0.26	19.14	23.18	-2.07	0.06	0.03

Notes: 1000 bootstrap samples. T: treated group. C: control group. Diff: absolute standardised difference.
KS: Kolmogorov-Smirnov test statistic. eCDF: mean difference in empirical CDFs.

Table S53. Peatland forest, $>$ 60% forest cover in 2005, matching up to 2010

	Unmatched					Matched				
	T	C	Diff	KS	eCDF	T	C	Diff	KS	eCDF
**Forest 2005**	127.17	122.55	22.74	0.16	0.09	127.03	126.93	0.51	0.04	0.02
**Forest 2006**	126.46	121.21	24.59	0.16	0.10	126.30	126.22	0.37	0.05	0.02
**Forest 2007**	125.67	119.68	26.44	0.17	0.10	125.48	125.45	0.13	0.04	0.02
**Forest 2008**	124.83	118.05	28.16	0.17	0.10	124.61	124.60	0.03	0.04	0.02
**Forest 2009**	123.81	116.00	30.34	0.18	0.11	123.55	123.56	-0.03	0.04	0.02
**Forest 2010**	122.93	114.56	30.80	0.18	0.11	122.64	122.58	0.20	0.05	0.02
**Elevation**	92.96	49.41	25.89	0.21	0.12	88.79	82.32	3.88	0.17	0.09
**Slope**	3.44	2.06	24.49	0.09	0.04	3.34	3.21	2.35	0.19	0.09
**Distance roads**	58.53	27.42	69.53	0.41	0.25	57.24	58.04	-1.77	0.18	0.09
**Distance cities**	1873.79	898.77	78.12	0.41	0.25	1829.01	1867.37	-3.09	0.15	0.04
**Distance palm oil**	63.94	73.52	-16.40	0.20	0.12	63.45	60.67	4.71	0.12	0.07
**Distance timber**	150.56	89.51	41.61	0.29	0.15	142.98	133.61	6.55	0.13	0.07
**Distance logging**	46.85	114.19	-111.88	0.22	0.12	47.89	46.62	2.06	0.11	0.05
**AGC**	124.77	72.15	102.93	0.45	0.26	122.32	123.65	-2.60	0.09	0.02
**Population 2005**	17.09	397.29	-229.70	0.41	0.25	17.97	21.50	-2.07	0.07	0.03
**Population 2010**	16.27	399.93	-212.99	0.42	0.26	17.10	21.07	-2.14	0.06	0.03

Notes: 1000 bootstrap samples. T: treated group. C: control group. Diff: absolute standardised difference.
KS: Kolmogorov-Smirnov test statistic. eCDF: mean difference in empirical CDFs.

Table S54. Peatland forest, $>$ 60% forest cover in 2005, matching up to 2004

	Unmatched					Matched				
	T	C	Diff	KS	eCDF	T	C	Diff	KS	eCDF
**Forest 2000**	128.57	125.12	17.59	0.14	0.07	128.43	128.56	-0.63	0.03	0.01
**Forest 2001**	128.34	124.66	18.68	0.14	0.08	128.20	128.32	-0.64	0.03	0.01
**Forest 2002**	128.05	124.16	19.62	0.15	0.08	127.90	128.03	-0.63	0.03	0.01
**Forest 2003**	127.89	123.88	20.18	0.15	0.08	127.74	127.86	-0.59	0.04	0.01
**Forest 2004**	127.54	123.21	21.55	0.15	0.09	127.37	127.50	-0.65	0.04	0.02
**Elevation**	92.96	49.41	25.89	0.21	0.12	88.56	80.98	4.56	0.17	0.09
**Slope**	3.44	2.06	24.49	0.09	0.04	3.33	3.16	3.07	0.19	0.09
**Distance roads**	58.53	27.42	69.53	0.41	0.25	57.21	57.96	-1.67	0.18	0.09
**Distance cities**	1873.79	898.77	78.12	0.41	0.25	1829.60	1870.73	-3.31	0.15	0.04
**Distance palm oil**	63.94	73.52	-16.40	0.20	0.12	63.42	60.84	4.36	0.12	0.07
**Distance timber**	150.56	89.51	41.61	0.29	0.15	142.77	134.00	6.14	0.13	0.07
**Distance logging**	46.85	114.19	-111.88	0.22	0.12	47.96	46.17	2.91	0.11	0.05
**AGC**	124.77	72.15	102.93	0.45	0.26	122.21	123.40	-2.33	0.09	0.02
**Population 2005**	17.09	397.29	-229.70	0.41	0.25	17.99	21.55	-2.09	0.06	0.03
**Population 2010**	16.27	399.93	-212.99	0.42	0.26	17.13	21.08	-2.13	0.06	0.03

Notes: 1000 bootstrap samples. T: treated group. C: control group. Diff: absolute standardised difference.
KS: Kolmogorov-Smirnov test statistic. eCDF: mean difference in empirical CDFs.

Table S55. Dryland forest, cells above 1000m, matching up to 2010

	Unmatched					Matched				
	T	C	Diff	KS	eCDF	T	C	Diff	KS	eCDF
**Forest 2005**	109.30	103.29	14.64	0.10	0.06	109.44	108.79	1.59	0.02	0.01
**Forest 2006**	108.58	102.23	15.32	0.10	0.06	108.70	108.02	1.64	0.02	0.01
**Forest 2007**	108.02	101.31	16.06	0.11	0.06	108.13	107.49	1.54	0.02	0.01
**Forest 2008**	107.43	100.34	16.85	0.11	0.06	107.54	106.90	1.53	0.02	0.01
**Forest 2009**	106.43	98.69	18.11	0.11	0.07	106.52	105.99	1.24	0.02	0.01
**Forest 2010**	105.83	97.69	18.86	0.11	0.07	105.91	105.37	1.26	0.02	0.01
**Elevation**	1634.42	1421.82	36.25	0.21	0.12	1584.76	1644.43	-10.84	0.06	0.02
**Slope**	19.75	13.38	93.64	0.36	0.24	19.41	19.21	3.17	0.03	0.02
**Distance roads**	34.24	19.95	52.28	0.32	0.20	34.08	36.55	-9.12	0.06	0.02
**Distance cities**	1915.67	1120.87	51.68	0.41	0.21	1883.70	2111.55	-15.13	0.12	0.07
**Distance palm oil**	95.77	135.35	-37.14	0.14	0.07	92.14	91.42	0.69	0.07	0.03
**Distance timber**	154.93	147.36	5.04	0.10	0.06	144.37	132.79	7.93	0.07	0.04
**Distance logging**	76.62	157.52	-57.13	0.16	0.08	72.46	65.27	5.25	0.08	0.03
**AGC**	159.03	110.94	167.21	0.49	0.29	159.03	159.07	-0.13	0.04	0.02
**Population 2005**	14.66	180.94	-160.89	0.39	0.27	13.36	15.88	-2.82	0.08	0.04
**Population 2010**	12.61	192.48	-153.10	0.40	0.28	11.50	14.08	-2.43	0.08	0.04

Notes: 1000 bootstrap samples. T: treated group. C: control group. Diff: absolute standardised difference.
KS: Kolmogorov-Smirnov test statistic. eCDF: mean difference in empirical CDFs.

Table S56. Peatland forest, cells above 1000m, matching up to 2010

	Unmatched					Matched				
	T	C	Diff	KS	eCDF	T	C	Diff	KS	eCDF
**Forest 2005**	115.92	118.13	-5.92	0.05	0.02	116.89	116.68	0.55	0.09	0.02
**Forest 2006**	115.65	117.77	-5.67	0.05	0.02	116.68	116.34	0.88	0.10	0.03
**Forest 2007**	115.43	117.44	-5.34	0.05	0.02	116.59	115.67	2.40	0.09	0.02
**Forest 2008**	115.27	117.09	-4.82	0.05	0.02	116.49	114.99	3.89	0.10	0.03
**Forest 2009**	114.96	116.58	-4.29	0.05	0.02	116.04	114.68	3.53	0.09	0.03
**Forest 2010**	114.65	116.27	-4.23	0.04	0.02	115.94	114.43	3.92	0.09	0.03
**Elevation**	1556.01	1568.82	-2.70	0.13	0.05	1505.52	1591.79	-17.44	0.19	0.09
**Slope**	22.45	13.72	99.12	0.37	0.24	20.92	21.39	-5.43	0.18	0.05
**Distance roads**	41.38	33.58	28.68	0.19	0.09	38.52	43.15	-16.53	0.19	0.05
**Distance cities**	2700.91	1808.45	49.44	0.30	0.16	2580.48	3082.23	-31.72	0.24	0.08
**Distance palm oil**	94.65	106.80	-19.80	0.13	0.04	97.98	97.90	0.13	0.19	0.07
**Distance timber**	251.93	246.14	4.68	0.17	0.06	257.39	242.91	11.92	0.16	0.06
**Distance logging**	45.27	71.69	-61.49	0.21	0.12	43.01	53.34	-37.60	0.30	0.14
**AGC**	150.48	109.44	116.33	0.47	0.29	149.33	144.63	12.80	0.22	0.09
**Population 2005**	9.07	88.07	-127.77	0.38	0.25	5.90	7.56	-12.73	0.17	0.07
**Population 2010**	8.90	116.32	-126.65	0.39	0.26	5.84	6.49	-4.78	0.12	0.05

Notes: 1000 bootstrap samples. T: treated group. C: control group. Diff: absolute standardised difference.
KS: Kolmogorov-Smirnov test statistic. eCDF: mean difference in empirical CDFs.

Table S57. Dryland forest, all-elevation, matching up to 2010

	Unmatched					Matched				
	T	C	Diff	KS	eCDF	T	C	Diff	KS	eCDF
**Forest 2005**	109.15	95.90	32.36	0.18	0.11	109.01	109.67	-1.60	0.01	0.01
**Forest 2006**	108.28	94.67	32.83	0.19	0.11	108.14	108.77	-1.53	0.02	0.01
**Forest 2007**	107.53	93.56	33.35	0.19	0.11	107.39	108.00	-1.47	0.02	0.01
**Forest 2008**	106.82	92.36	34.14	0.19	0.11	106.67	107.31	-1.50	0.02	0.01
**Forest 2009**	105.70	90.68	34.91	0.19	0.12	105.55	106.17	-1.46	0.02	0.01
**Forest 2010**	105.02	89.67	35.27	0.19	0.12	104.86	105.46	-1.38	0.02	0.01
**Elevation**	816.19	271.64	82.35	0.50	0.31	804.01	810.41	-0.98	0.06	0.03
**Slope**	15.10	6.53	116.55	0.51	0.32	14.99	15.00	-0.07	0.04	0.01
**Distance roads**	34.88	12.74	71.53	0.43	0.28	34.65	37.11	-7.95	0.07	0.04
**Distance cities**	1685.47	607.63	76.43	0.45	0.26	1668.69	1728.52	-4.27	0.09	0.03
**Distance palm oil**	100.39	166.04	-49.76	0.21	0.07	100.59	100.86	-0.21	0.04	0.02
**Distance timber**	133.08	129.05	2.75	0.12	0.06	132.00	115.03	11.61	0.05	0.02
**Distance logging**	84.10	226.23	-86.52	0.31	0.18	84.68	84.27	0.25	0.04	0.02
**AGC**	158.18	93.16	182.13	0.58	0.33	157.97	155.70	6.34	0.04	0.02
**Population 2005**	16.51	320.78	-240.16	0.47	0.28	16.67	18.72	-1.60	0.07	0.04
**Population 2010**	14.24	321.69	-247.64	0.49	0.29	14.39	16.65	-1.81	0.07	0.04

Notes: 1000 bootstrap samples. T: treated group. C: control group. Diff: absolute standardised difference.
KS: Kolmogorov-Smirnov test statistic. eCDF: mean difference in empirical CDFs.

Table S58. Peatland forest, all-elevation cells, matching up to 2010

	Unmatched					Matched				
	T	C	Diff	KS	eCDF	T	C	Diff	KS	eCDF
**Forest 2005**	111.53	98.58	32.54	0.19	0.11	111.13	111.17	-0.10	0.03	0.01
**Forest 2006**	110.76	97.28	33.41	0.19	0.11	110.34	110.40	-0.16	0.03	0.02
**Forest 2007**	109.94	95.93	34.23	0.20	0.12	109.50	109.55	-0.13	0.03	0.01
**Forest 2008**	109.11	94.53	35.09	0.20	0.12	108.64	108.70	-0.12	0.03	0.01
**Forest 2009**	108.09	92.74	36.25	0.20	0.12	107.60	107.64	-0.08	0.04	0.02
**Forest 2010**	107.24	91.53	36.52	0.20	0.12	106.73	106.73	0.01	0.04	0.02
**Elevation**	116.39	57.84	21.72	0.19	0.11	112.14	101.69	3.92	0.18	0.10
**Slope**	3.67	2.06	25.93	0.10	0.04	3.56	3.31	4.04	0.19	0.09
**Distance roads**	54.08	24.21	68.89	0.40	0.26	52.93	54.08	-2.65	0.15	0.09
**Distance cities**	1757.89	790.56	76.12	0.41	0.25	1713.34	1763.31	-3.98	0.13	0.04
**Distance palm oil**	60.34	77.95	-29.17	0.17	0.10	59.81	57.08	4.48	0.13	0.08
**Distance timber**	136.64	90.13	32.32	0.24	0.12	130.31	120.73	6.83	0.14	0.08
**Distance logging**	49.56	125.17	-118.79	0.24	0.13	50.47	49.44	1.59	0.12	0.06
**AGC**	120.29	68.71	98.29	0.44	0.26	118.15	118.71	-1.07	0.08	0.02
**Population 2005**	21.62	427.10	-215.25	0.41	0.25	22.44	26.42	-2.07	0.05	0.03
**Population 2010**	20.00	433.05	-208.98	0.42	0.26	20.75	24.99	-2.10	0.06	0.03

Notes: 1000 bootstrap samples. T: treated group. C: control group. Diff: absolute standardised difference.
KS: Kolmogorov-Smirnov test statistic. eCDF: mean difference in empirical CDFs.

Table S59. Dryland "intact primary" forest, matching up to 2010

	Unmatched					Matched				
	**T**	**C**	**Diff**	**KS**	**eCDF**	**T**	**C**	**Diff**	**KS**	**eCDF**
**Primary forest 2005**	80.40	52.40	43.99	0.20	0.19	79.00	78.99	0.03	0.04	0.03
**Primary forest 2006**	80.09	52.06	44.02	0.20	0.19	78.68	78.64	0.07	0.04	0.03
**Primary forest 2007**	79.77	51.71	44.05	0.21	0.19	78.37	78.30	0.10	0.04	0.03
**Primary forest 2008**	79.49	51.37	44.14	0.21	0.19	78.08	78.00	0.13	0.03	0.03
**Primary forest 2009**	79.03	50.85	44.19	0.21	0.19	77.63	77.52	0.17	0.03	0.03
**Primary forest 2010**	78.73	50.48	44.28	0.21	0.19	77.32	77.18	0.22	0.03	0.03
**Elevation**	470.04	207.62	96.65	0.47	0.28	457.63	459.76	-0.79	0.07	0.03
**Slope**	13.14	6.15	104.81	0.47	0.29	12.85	13.14	-4.32	0.07	0.02
**Distance roads**	35.15	12.34	70.50	0.42	0.28	33.70	35.46	-5.56	0.09	0.05
**Distance cities**	1588.08	579.06	75.26	0.43	0.26	1512.67	1496.02	1.29	0.13	0.04
**Distance palm oil**	102.35	167.75	-46.31	0.20	0.07	103.96	102.84	0.78	0.07	0.04
**Distance timber**	123.84	128.03	-2.91	0.10	0.05	121.04	103.89	12.01	0.07	0.04
**Distance logging**	87.26	230.05	-82.59	0.32	0.20	90.49	93.82	-1.89	0.06	0.04
**AGC**	157.82	92.17	171.62	0.57	0.33	157.02	154.96	5.31	0.02	0.01
**Population 2005**	17.30	328.56	-229.96	0.49	0.28	18.05	21.23	-2.29	0.07	0.04
**Population 2010**	14.92	328.89	-247.47	0.50	0.29	15.59	18.89	-2.54	0.06	0.04

Notes: 1000 bootstrap samples. T: treated group. C: control group. Diff: absolute standardised difference.
KS: Kolmogorov-Smirnov test statistic. eCDF: mean difference in empirical CDFs.

Table S60. Peatland "intact primary" forest, matching up to 2010

	Unmatched					Matched				
	T	C	Diff	KS	eCDF	T	C	Diff	KS	eCDF
**Primary forest 2005**	87.07	57.25	47.97	0.22	0.20	86.12	85.76	0.59	0.02	0.01
**Primary forest 2006**	86.80	56.86	48.11	0.22	0.20	85.85	85.49	0.57	0.02	0.01
**Primary forest 2007**	86.46	56.36	48.33	0.22	0.20	85.50	85.15	0.57	0.02	0.01
**Primary forest 2008**	86.12	55.91	48.46	0.22	0.20	85.16	84.81	0.56	0.02	0.01
**Primary forest 2009**	85.67	55.32	48.63	0.23	0.20	84.70	84.33	0.60	0.02	0.01
**Primary forest 2010**	85.22	54.79	48.70	0.23	0.20	84.24	83.84	0.65	0.02	0.01
**Elevation**	87.08	48.09	23.94	0.19	0.11	83.72	75.37	5.17	0.18	0.10
**Slope**	3.29	1.98	23.62	0.08	0.03	3.22	3.03	3.44	0.19	0.09
**Distance roads**	54.33	24.15	69.26	0.40	0.26	53.10	54.31	-2.77	0.15	0.09
**Distance cities**	1738.69	783.99	76.37	0.41	0.25	1696.50	1741.37	-3.62	0.13	0.04
**Distance palm oil**	59.65	77.76	-30.13	0.17	0.10	59.18	56.66	4.15	0.13	0.08
**Distance timber**	134.29	89.12	31.52	0.24	0.12	127.92	118.59	6.69	0.14	0.09
**Distance logging**	49.65	125.51	-118.54	0.24	0.13	50.60	49.77	1.27	0.12	0.06
**AGC**	119.68	68.44	97.41	0.43	0.26	117.55	118.10	-1.05	0.08	0.02
**Population 2005**	21.88	429.29	-214.34	0.41	0.25	22.72	26.74	-2.07	0.05	0.03
**Population 2010**	20.22	435.10	-208.19	0.42	0.26	21.00	25.39	-2.16	0.06	0.03

Notes: 1000 bootstrap samples. T: treated group. C: control group. Diff: absolute standardised difference.
KS: Kolmogorov-Smirnov test statistic. eCDF: mean difference in empirical CDFs.

Robustness

Sensitivity to matching procedures. We conduct additional sensitivity analyses on the assumptions used in propensity score matching estimates and a robustness analysis of the central NP-DDD on alternative approaches to matching procedures that do not rely on the propensity score. In part these test the possibility that propensity score matching may introduce biases due to the way in which it reduces the dimensionality of the matching problem to matching on a single dimension: the propensity score (e.g. (45)). On the sensitivity of the propensity score estimates to the parameter choices in implementation, the analyses are: (1) $k > 1$ nearest neighbours analysis in which we test the sensitivity of our results to matching on $k = 2, 3, 5$ nearest neighbours; and, (2) caliper choices, where we test calipers of 0.01 and 0.001 in one-to-one nearest neighbour matching. With regard to alternative, non-propensity score, matching estimators we undertake Coarsened Exact Matching (CEM) (50, 51). The use of CEM means that the sample sizes are slightly more sensitive to particular choices of matching variables and the coarseness of the matching. For this reason we undertake four separate CEM approaches with various degrees of coarseness to understand these sensitivities. We use the STATA 16 cem routine and default coarseness settings unless otherwise stated (See table notes). Tables S13 and S14 shows the results of the sensitivity analyses on dryland and peatland forests. The results show that the main results are not being driven by the choice of the propensity score matching estimator over CEM. Tables S13 and S14 show that the CEM estimates do not differ to any great extent for peatland and dryland forest. Furthermore, Tables S17 and S18 show that the main results are robust to the number of nearest neighbours chosen for 2,3 and 5 neighbours. Finally, our choice of sample removed areas above 1,000 m for agronomic reasons. Tables S17 and S18 also show that this selection of the sample is not driving the results either. Overall, these results are strongly supportive of our estimation approach leading to our main results.

Testing the SUTVA assumption using Regression Discontinuity: 'Leakage'? . A final identification assumption of the DD and DDD estimators is the Stable Unit Treatment Value Assumption (SUTVA): the treatment causes leakage to untreated forest areas via spatial, behavioural or general equilibrium effects, a common confounder in the evaluation of area-based policies (27, 28, 38, 39, 52). If protection via the Moratorium induces the displacement of forest clearing to outside the Moratorium's boundaries, deforestation rates inside and outside these boundaries are contemporaneously affected in opposite directions resulting in treatment effects of a higher magnitude than the "true" effects. To check for leakage, we use a regression discontinuity design (53, 54) with a sharp cut-off at the Moratorium's boundaries. Regression discontinuity estimates the LATE of the Moratorium in the proximity of its boundaries with non-Moratorium land. In particular, we estimate the following equation (following (55)):

$$D_i = \alpha + \beta_1 Moratorium_i + \beta_2 X_i + f(Distance_i) + \epsilon_i$$

where D_i is deforestation (in pixels) in grid cell i; $Moratorium_i$ is a binary indicator representing treatment assignment, X_i is a vector of cross-sectional control variables, namely slope, elevation, distance from cities and roads, and above-ground biomass content, and

$$f(Distance_i) = Moratorium_i * f_{Moratorium}(Distance_i) + \\ + (1 - Moratorium_i) f_{Non-Moratorium}(Distance_i)$$

is a polynomial term which weighs observations with respect to their distance from the Moratorium's boundaries.

We specify separate linear polynomials on both sides of the boundary cut-off (following (56) and (55)), and estimate treatment effects via OLS regressions with robust standard errors clustered at the district level. Our preferred results are obtained via separate linear polynomials and optimal bandwidth selection through the Calonico (54) method. In Fig. S7, we also present results using the Imbens-Kalyanaraman (57) bandwidth selection algorithm (panel B).

In Fig. S8-S12, we visualise the full dataset employed in the spatial regression discontinuity designs, letting the bandwidth size vary according to either the Calonico (54) or the Imbens-Kalyanaraman (57) method. First, we report two plots which relate the forcing variable, distance from the Moratorium border, to forest cover in pixels, for 2004 and 2010 (Fig. S8). We treat 2004 as the "base year" since we estimate regression discontinuity LATEs for 2005-2018. As shown in Fig. S8 (left panel), forest cover is not distributed smoothly around the threshold at the baseline, with Moratorium grid cells discontinuously more forested than the surrounding areas. Fig. S8 (right panel) confirms the trend for 2010, the actual baseline year prior to the implementation of the Moratorium. These descriptive results are a confirmation of the economic marginality of Moratorium grid cells, which tended to be cleared at a slower rate than non-Moratorium grid cells in the immediate vicinity, even before the Moratorium was implemented.

In Fig. S9 and S10, we report the discontinuity in deforestation rates around Moratorium boundaries, restricted to the bandwidth level calculated with our preferred Calonico (54) procedure. Fig. S9 examines pre-treatment years while Fig. S10 visualises data for 2011-2018. Clearly, deforestation is persistently higher outside of Moratorium boundaries, starting from 2005 and up until 2018, with only slight changes in the underlying data distribution. In Fig. S11 and S12, we report analogous regression discontinuity plots using the Imbens-Kalyanaraman (57) optimal bandwidth selection procedure, which yield similar results: deforestation rates are always higher outside the Moratorium, enhancing our confidence in ruling out the occurrence of spatial leakage.

We perform several robustness checks to assess the validity of our regression discontinuity estimates, which rule out the potential role of leakage in our results. In particular, we first run regressions with alternative bandwidths, namely: a 5 km fixed

bandwidth; a 10 km fixed bandwidth; and, a 20 km fixed bandwidth (reported in the top row of Fig. S13). Given the spatial configuration of the Moratorium (Fig. S2), our bandwidths cover a large extent of non-Moratorium cells in which leakage could feasibly occur. One reason why we might not observe a leakage effect is because the Moratorium's boundaries have changed after forest areas dropped out of the Moratorium. This can be ruled out, at least partially, by the application of these different bandwidths.

The sensitivity of our results is tested with respect to alternative polynomial specifications, employing a quadratic function of distance on both sides of the cut-off using: the Imbens-Kalyanaraman (57) optimal bandwidth; a 10 km fixed bandwidth; and, the Calonico (54) optimal bandwidth. These results are reported in the bottom row of Fig. S13. Notably, the 95% confidence interval for the regression discontinuity LATE always includes zero, thereby reassuring us about the stability of our estimates with respect to alternative specifications. We are therefore able to rule out the role of leakage in affecting our main results.

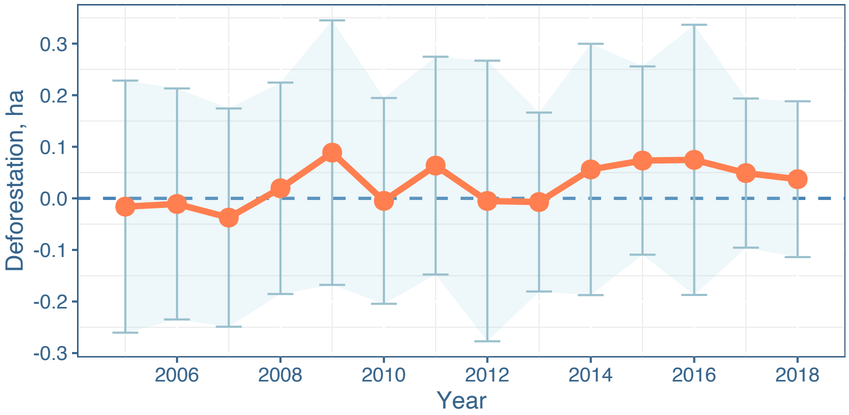

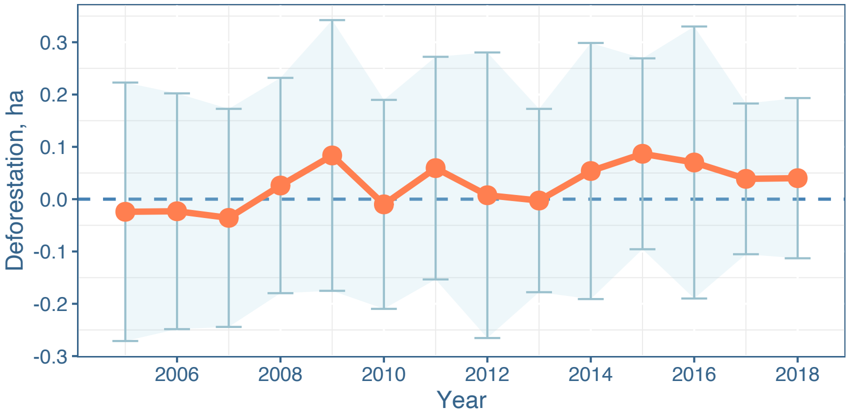

Fig. S7. Regression discontinuity LATE with Calonico (54) bandwidth (A); regression discontinuity LATE with Imbens-Kalyanaraman (57) bandwidth (B).

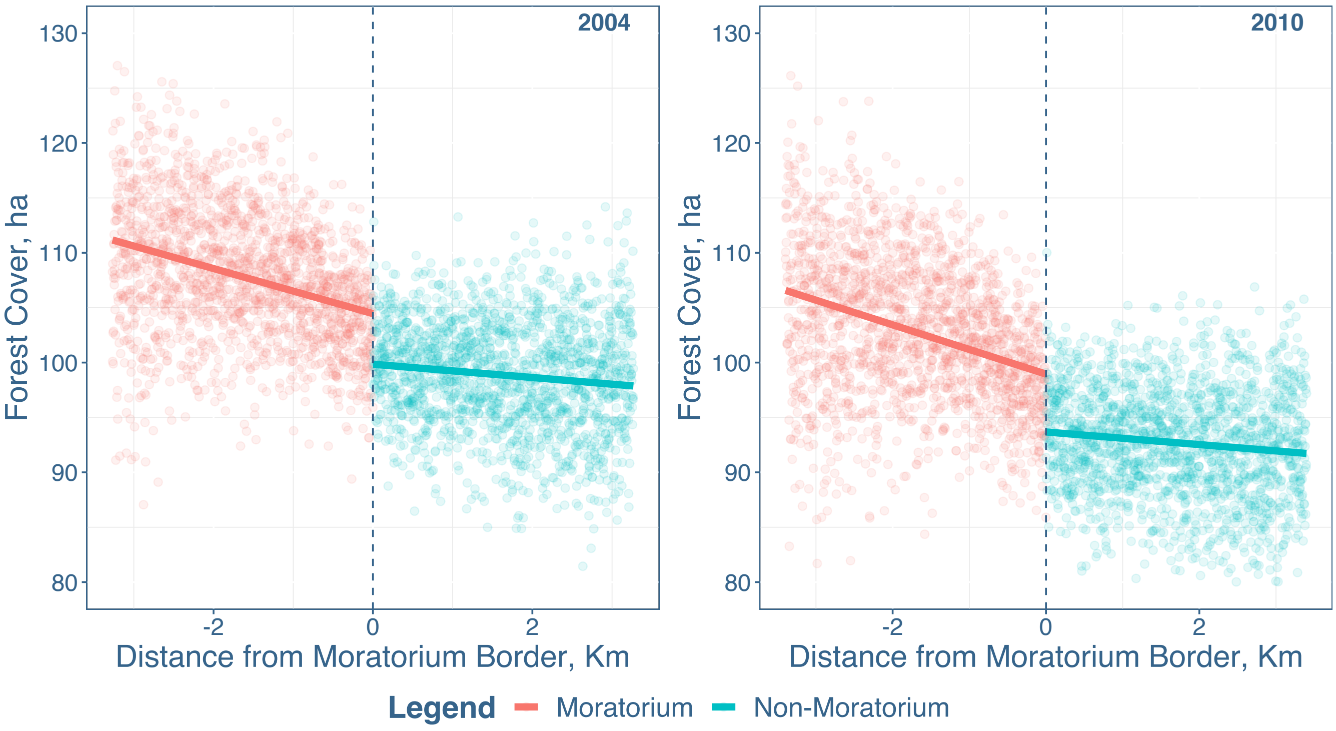

Fig. S8. Forest cover in (A) 2004 and (B) 2010, with respect to distance from Moratorium boundaries

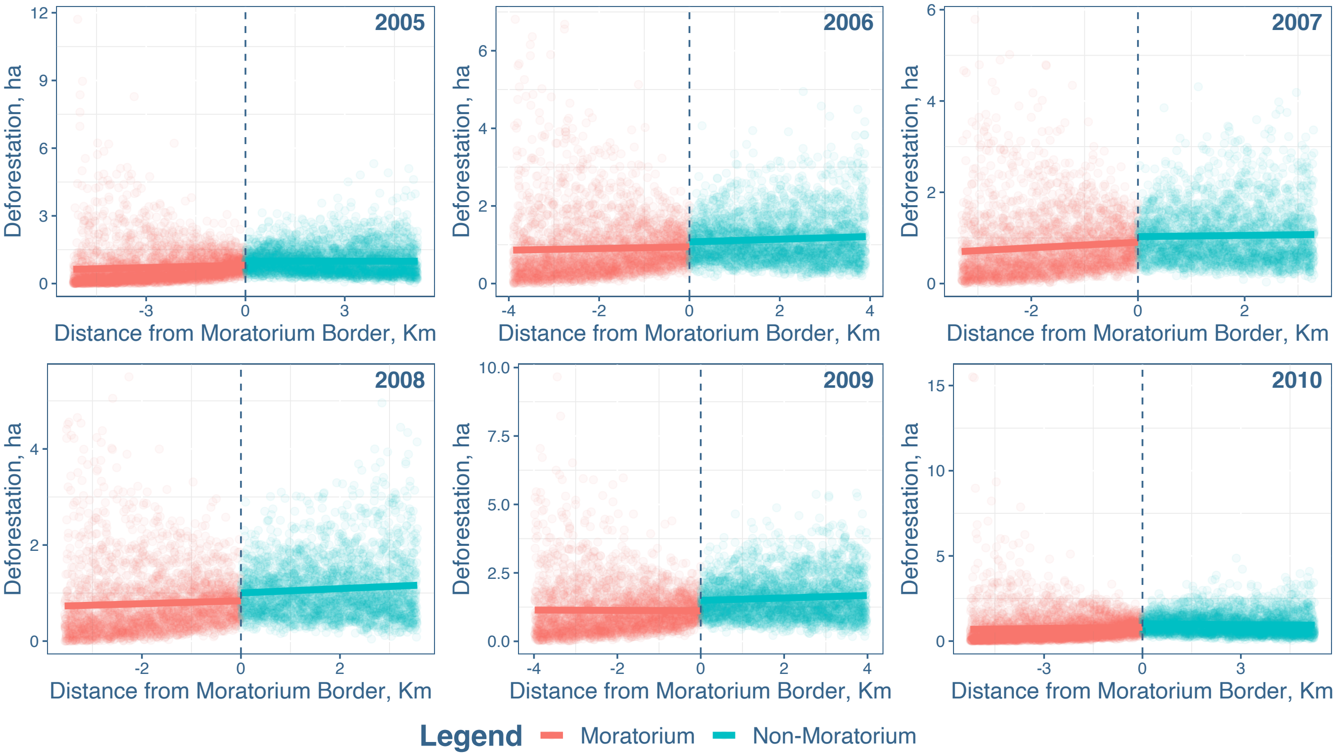

Fig. S9. Deforestation in the proximity of Moratorium boundaries, Calonico (54) bandwidth, pre-treatment years.

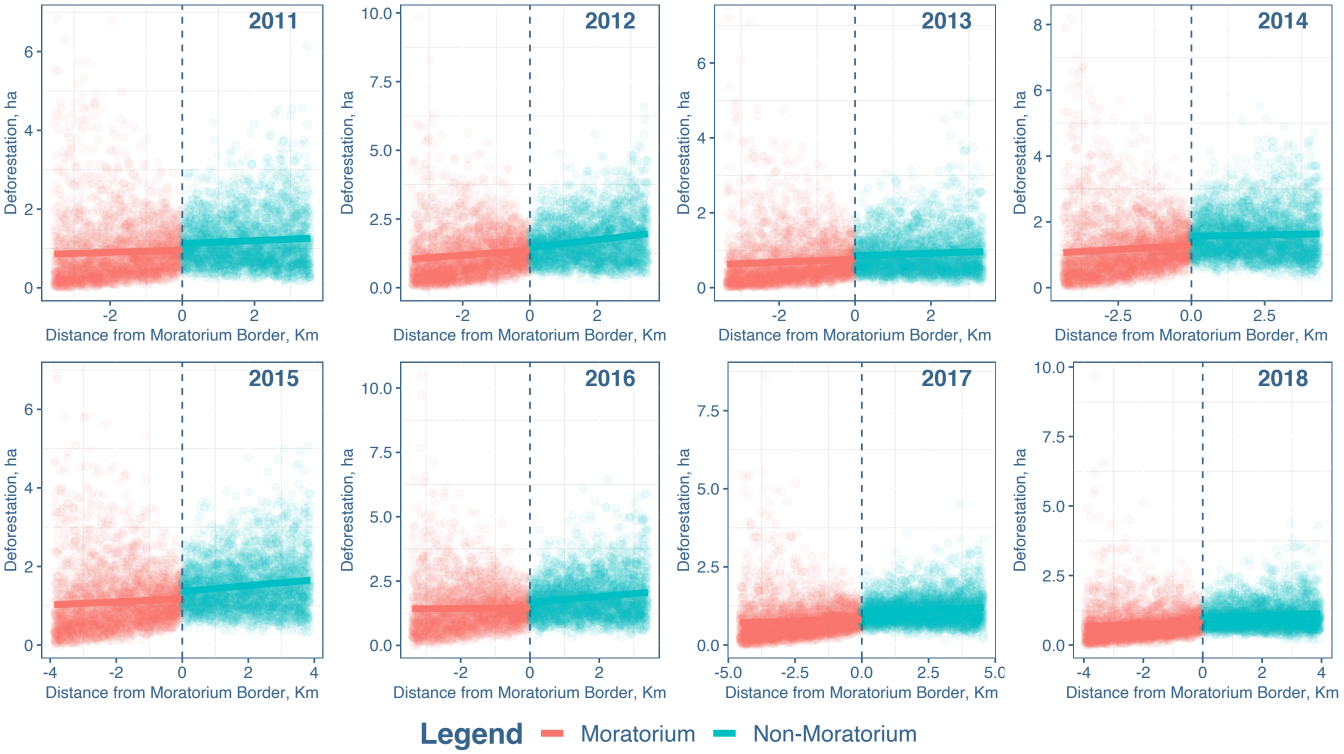

Fig. S10. Deforestation in the proximity of Moratorium boundaries, Calonico (54) bandwidth, post-treatment years.

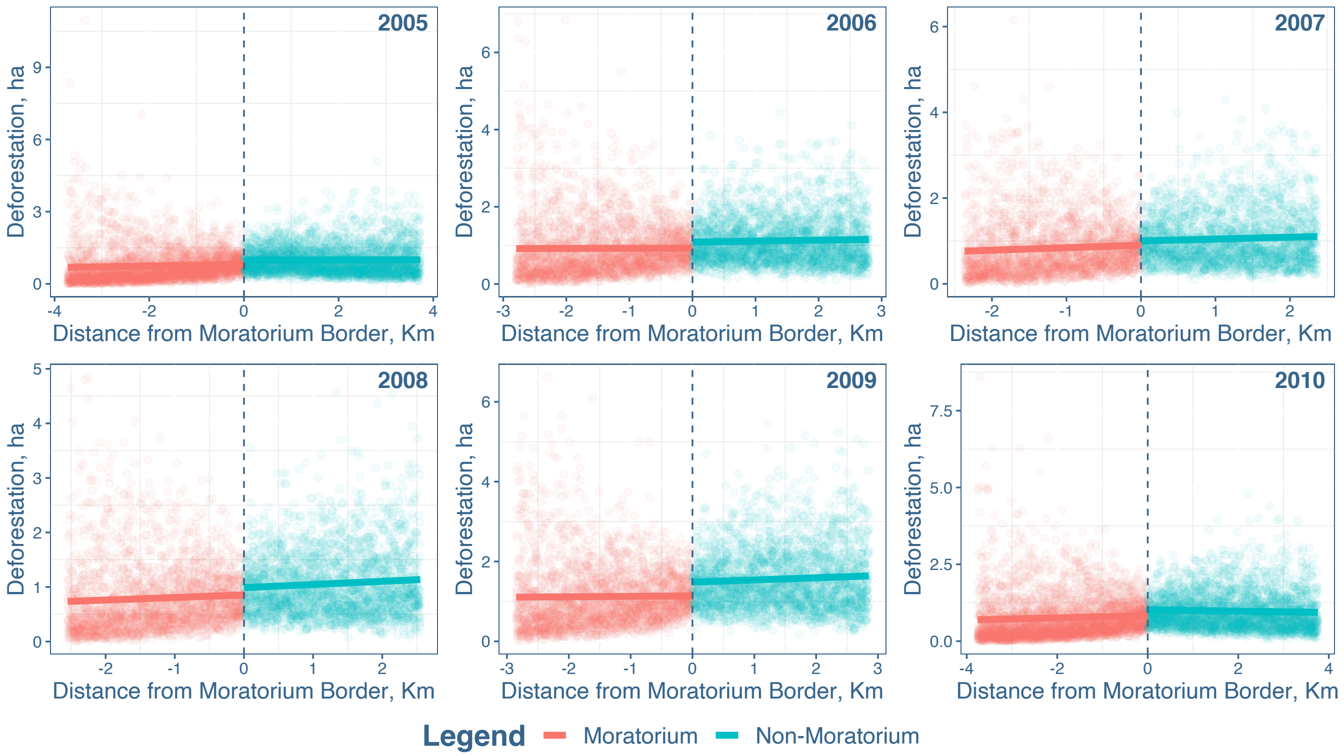

Fig. S11. Deforestation in the proximity of Moratorium boundaries, Imbens-Kalyanaraman(57) bandwidth, pre-treatment years.

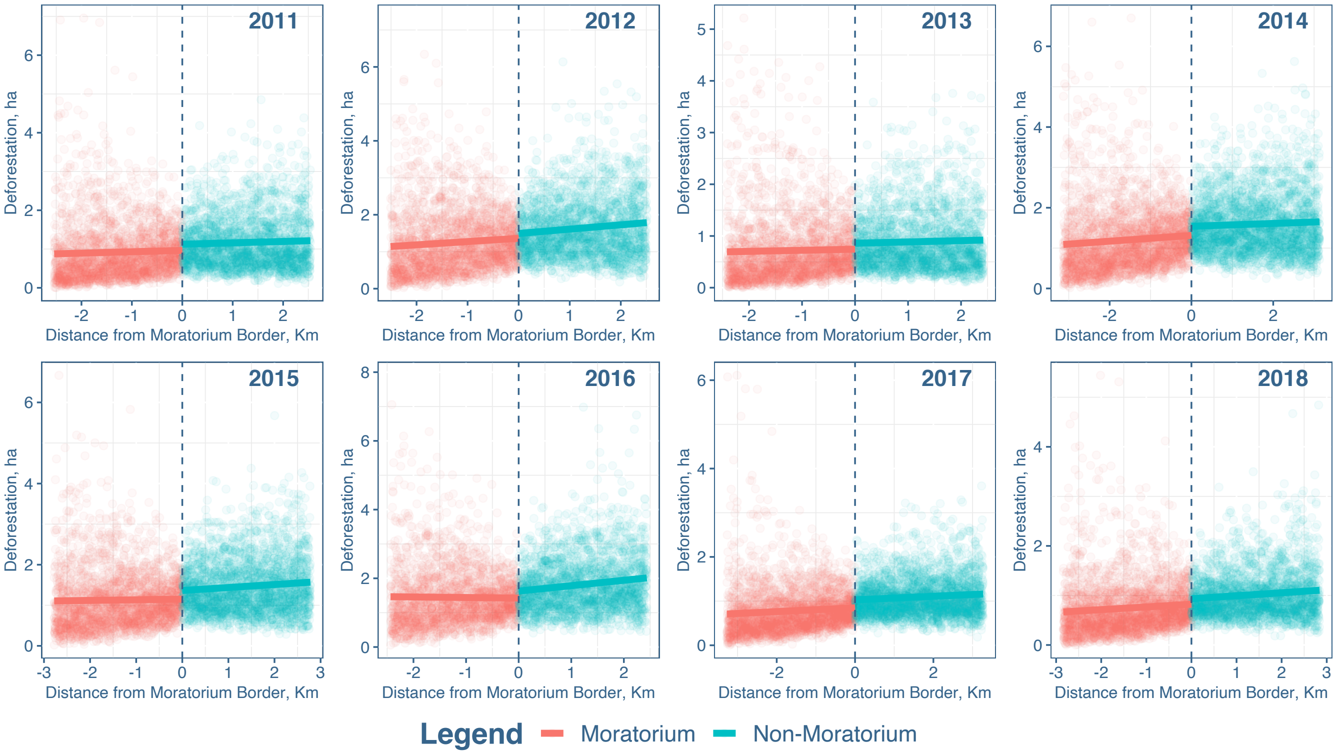

Fig. S12. Deforestation in the proximity of Moratorium boundaries, Imbens-Kalyanaraman (57) bandwidth, post-treatment years.

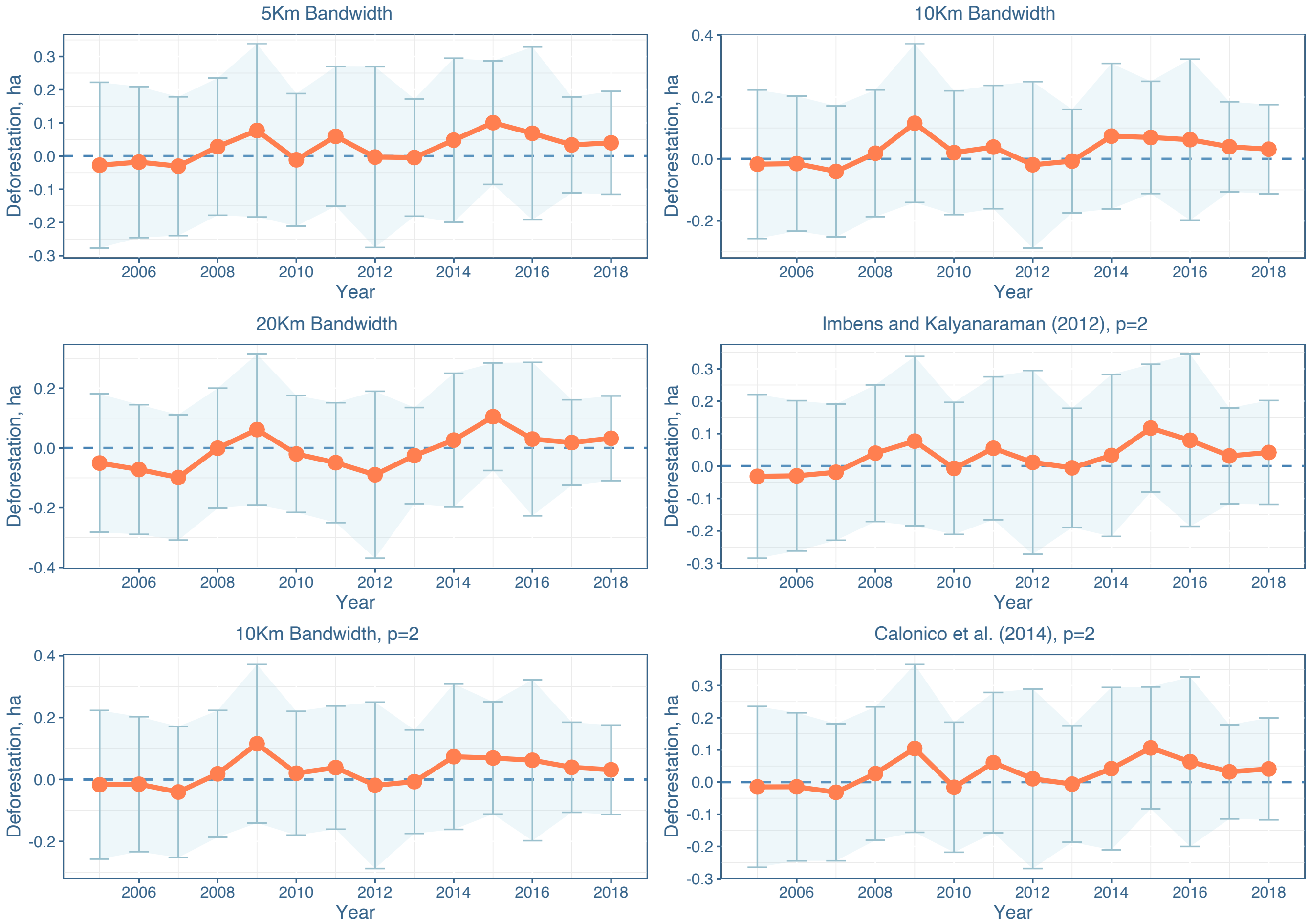

Fig. S13. Regression discontinuity robustness checks

References

1. Mongabay, Experts question integrity of indonesia's claim of avoided deforestation (2020).
2. L International, Third independent review of the indonesia-norway cooperation on reducing greenhouse gas emissions from redd+, (LTS International), Technical report (2018).
3. Mongabay, Indonesia terminates agreement with norway on $1b redd+ scheme (2021).
4. CH News, Indonesia ends forest protection deal with norway, raising deforestation fears (2021).
5. FW Indonesia, The state of forest report in indonesia, (Forest Watch Indonesia, Bogor, Indonesia), Brussels presentation, may 2015 (2015).
6. MC Hansen, et al., High-resolution global maps of 21st-century forest cover change. *Science* **342**, 850–853 (2013).
7. Ministry of Forestry of the Republic of Indonesia, Indicative map of new revised permit delays 5 (2011).
8. Mongabay, Indonesia forest-clearing ban is made permanent, but labeled "propaganda" (2019).
9. G Indrarto, et al., The context of redd+ in indonesia: drivers, agents and institutions, (Center for International Forestry Research (CIFOR), Bogor, Indonesia), Technical Report 92 (2012).
10. A Enrici, K Hubacek, Business as usual in indonesia: governance factors effecting the acceleration of the deforestation rate after the introduction of redd+. *Energy, Ecol. Environ.* **1**, 183–196 (2016).
11. BA Margono, et al., Mapping and monitoring deforestation and forest degradation in sumatra (indonesia) using landsat time series data sets from 1990 to 2010. *Environ. Res. Lett.* **7**, 34010 (2012).
12. FAO, Global forest resources assessment 2015 country report: Indonesia, (Food and Agriculture Organization of the United Nations, Rome.), Country report (2015).
13. SA Abood, et al., Relative contributions of the logging, fiber, oil palm, and mining industries to forest loss in Indonesia. *Conserv. Lett.* **8**, 58–67 (2015).
14. T Steinweg, B Kuepper, M Piotrowski, 28 percent of indonesia's palm oil landbank is stranded, (Chain Reaction Research, Washington D.C.), Working paper (2019).
15. KG Austin, A Schwantes, Y Gu, PS Kasibhatla, What causes deforestation in indonesia? *Environ. Res. Lett.* **14**, 024007 (2019).
16. AJ Glauber, S Moyer, M Adriani, I Gunawan, The cost of fire : An economic analysis of indonesia's 2015 fire crisis, (World Bank, Jakarta, Indonesia), Technical Report 1 (2016).
17. AS Alisjahbana, JM Busch, Forestry, forest fires, and climate change in indonesia. *Bull. Indonesian Econ. Stud.* **53**, 111–136 (2017).
18. MoEF, Emissions reduction report for the indonesia-norway partnership, (Directorate General of Climate Change, Ministry of Environment and Forestry, Republic of Indonesia), government report (2019).
19. RoI, Indonesia second biennial update report under the united nations framework convention on climate change, (Directorate General of Climate Change, Ministry of Environment and Forestry, Government of Indonesia), government report (2018).
20. RoI, National forest reference emission level for deforestation and forest degradation: Submission by indonesia, (Directorate General of Climate Change. The Ministry of Environment and Forestry, Government of Indonesia.), Post technical assessment by unfccc (2016).
21. A Abadie, GW Imbens, Large sample properties of matching estimators. *Econometrica* **74**, 235–267 (2006).
22. BA Margono, PV Potapov, S Turubanova, F Stolle, MC Hansen, Primary forest cover loss in indonesia over 2000–2012. *Nat. Clim. Chang.* **4**, 730–735 (2014).
23. S Turubanova, PV Popapov, A Tyukavina, MC Hansen, Ongoing primary forest loss in brazil, democratic republic of the congo, and indonesia. *Environ. Res. Lett.* **13**, 074028 (2018).
24. S Chabé-Ferret, J Subervie, How much green for the buck? estimating additional and windfall effects of french agro-environmental schemes by did-matching. *J. Environ. Econ. Manag.* **65**, 12–27 (2013).
25. S Chabé-Ferret, Should We Combine Difference In Differences with Conditioning on Pre-Treatment Outcomes?, (Toulouse School of Economics, INRA and IAST, Toulouse), Technical report (2017).
26. HJ Albers, P Ferraro, The economics of terrestrial biodiversity conservation in developing nations in *Economic Development and Environmental Sustainability*, eds. R Lòpez, M Toman. (Oxford University Press, Oxford), (2006).
27. KS Andam, PJ Ferraro, A Pfaff, GA Sanchez-Azofeifa, JA Robalino, Measuring the effectiveness of protected area networks in reducing deforestation. *Proc. Natl. Acad. Sci.* **105**, 16089–16094 (2008).
28. L Joppa, A Pfaff, Reassessing the forest impacts of protection. *Annals New York Acad. Sci.* **1185**, 135–149 (2010).
29. S Sloan, DP Edwards, WF Laurance, Does indonesia's redd+ moratorium on new concessions spare imminently threatened forests? *Conserv. Lett.* **5**, 222–231 (2012).
30. G Imbens, Matching methods in practice: Three examples, (National Bureau of Economic Research), Working Paper 19959 (2014).
31. JM Wooldridge, *Econometric Analysis of Cross Section and Panel Data.* (The MIT Press), (2010).
32. A Abadie, GW Imbens, Notes and comments on the failure of the bootstrap for matching estimators. *Econometrica* **76**, 1537–1557 (2008).
33. KG Austin, PS Kasibhatla, DL Urban, F Stolle, J Vincent, Reconciling oil palm expansion and climate change mitigation in kalimantan, indonesia. *PLOS ONE* **10**, 1–17 (2015).
34. H Krisnawati, M Kallio, M Kanninen, *Acacia Mangium* Willd.: ecology, silviculture and productivity, (CIFOR, Bogor, Indonesia), Technical report (2011).

35. Mj Lee, *Micro-Econometrics for Policy, Program and Treatment Effects*, Advanced Texts in Econometrics. (Oxford University Press, Oxford), (2005).
36. J Wooldridge, Two-way fixed effects, the two-way mundlak regression, and event study estimators (2021).
37. M Caliendo, S Kopeinig, Some practical guidance for the implementation of propensity score matching. *J. Econ. Surv.* **22**, 31–72 (2008).
38. DL Gaveau, et al., Evaluating whether protected areas reduce tropical deforestation in Sumatra. *J. Biogeogr.* **36**, 2165–2175 (2009).
39. A Nelson, KM Chomitz, Effectiveness of strict vs. multiple use protected areas in reducing tropical forest fires: A global analysis using matching methods. *PLoS ONE* **6** (2011).
40. JJ Heckman, H Ichimura, P Todd, Matching as an econometric evaluation estimator: Evidence from evaluating a job training programme. *Rev. Econ. Stud.* **64**, 605–654 (1997).
41. PC Austin, Some methods of propensity-score matching had superior performance to others: results of an empirical investigation and monte carlo simulations. *Biom. J.* **51**, 171–184 (2009).
42. A Olden, J Møen, The triple difference estimator, (Norwegian School of Economics, Department of Business and Management Science), Discussion Papers 2020/1 (2020).
43. JJ Heckman, H Ichimura, P Todd, Matching as an econometric evaluation estimator. *Rev. Econ. Stud.* **65**, 261–294 (1998).
44. AM Ryan, K Evangelos, L Ariel, JF Burgess, Now trending: Coping with non-parallel trends in difference-in-differences analysis. *Stat. Methods Med. Res.* **28**, 3697–3711 (2018).
45. G King, R Nielsen, Why propensity scores should not be used for matching. *Polit. Analysis* **27**, 435–454 (2019).
46. K Imai, IS Kim, On the use of two-way fixed effects regression models for causal inference with panel data. *Polit. analysis* **29**, 405–415 (2021).
47. JR Daw, LA Hatfield, Matching in difference-in-differences: between a rock and a hard place. *Heal. Serv. Res.* **53**, 4111–4117 (2018).
48. M Ravallion, E Galasso, T Lazo, E Philipp, What can ex-participants reveal about a program's impact? *J. Hum. Resour.* **40** (2005).
49. J Gruber, The incidence of mandated maternity benefits. *The Am. Econ. Rev.* **84**, 622–641 (1994).
50. G King, L Zeng, The dangers of extreme counterfactuals. *Polit. Analysis* **14**, 131–159 (2006).
51. SM Iacus, G King, G Porro, Causal inference without balance checking: Coarsened exact matching. *Polit. analysis* **20**, 1–24 (2012).
52. LN Joppa, A Pfaff, Global protected area impacts. *Proc. Royal Soc. B* **278**, 1633–1638 (2010).
53. DS Lee, T Lemieux, Regression discontinuity designs in economics. *J. Econ. Lit.* **48**, 281–355 (2010).
54. S Calonico, MD Cattaneo, R Titiunik, Robust nonparametric confidence intervals for regression-discontinuity designs. *Econometrica* **82**, 2295–2326 (2014).
55. R Burgess, F Costa, B Olken, The brazilian amazon's double reversal of fortune (2019) Working Paper.
56. A Gelman, G Imbens, Why high-order polynomials should not be used in regression discontinuity designs. *J. Bus. & Econ. Stat.* **37**, 447–456 (2019).
57. G Imbens, K Kalyanaraman, Optimal bandwidth choice for the regression discontinuity estimator. *The Rev. Econ. Stud.* **79**, 933–959 (2012).
